# Improved Photostability of a Cu^I^ Complex by Macrocyclization of the Phenanthroline Ligands

**DOI:** 10.1002/chem.201904754

**Published:** 2020-02-18

**Authors:** Thomas Brandl, Christoph Kerzig, Loïc Le Pleux, Alessandro Prescimone, Oliver S. Wenger, Marcel Mayor

**Affiliations:** ^1^ Department of Chemistry University of Basel St. Johanns-Ring 19 4056 Basel Switzerland; ^2^ Karlsruhe Institute of Technology (KIT) P.O. Box 3640 76021 Karlsruhe Germany; ^3^ Lehn Institute of Functional Materials School of Chemistry Sun Yat-Sen University Guangzhou 510275 China

**Keywords:** copper complexes, ligand design, macrocycles, photochemistry, photostability

## Abstract

The development of molecular materials for conversion of solar energy into electricity and fuels is one of the most active research areas, in which the light absorber plays a key role. While copper(I)‐bis(diimine) complexes [Cu^I^(L)_2_]^+^ are considered as potent substitutes for [Ru^II^(bpy)_3_]^2+^, they exhibit limited structural integrity as ligand loss by substitution can occur. In this article, we present a new concept to stabilize copper bis(phenanthroline) complexes by macrocyclization of the ligands which are preorganized around the Cu^I^ ion. Using oxidative Hay acetylene homocoupling conditions, several Cu^I^ complexes with varying bridge length were prepared and analyzed. Absorption and emission properties are assessed; rewardingly, the envisioned approach was successful since the flexible 1,4‐butadiyl‐bridged complex does show enhanced MLCT absorption and emission, as well as improved photostability upon irradiation with a blue LED compared to a reference complex.

## Graphical abstract

**Rescue Cu^I^ phenanthroline complexes**: Like a safety buoy the macrocyclized bisphenanthroline ligand surrounds the Cu^I^ ion and improves the complex's photostability, visible‐light absorptivity, and emission compared to an open analogue (see graphic).

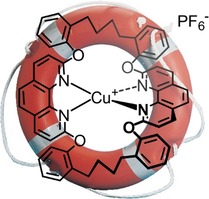

## Introduction

Increasing energy demand leads to the development of new strategies to convert solar energy into electricity. More efficient and less costly alternatives to silicon‐based photovoltaic devices are desirable and dye‐sensitized solar cells (DSCs) might have the potential to replace them in the future.

The model compounds used so far are based on Ru^II^ polypyridyl dyes displaying high efficiencies,^1^, ^2^, ^3^ but ruthenium as a less abundant element is handicapping any large scale device production and motivates the quest for alternatives. In their pioneering work, McMillin and co‐workers suggested Cu^I^ polypyridyl compounds as a potential replacement.^4^, ^5^, ^6^ Copper complexes are an inexpensive and an appealing alternative for the scarce ruthenium^7^, ^8^, ^9^, ^10^ as they absorb visible light due to the metal‐to‐ligand charge‐transfer band, and luminesce from their lowest excited state, at least as optimized structures in suitable solvents.^11^ The d^10^ electronic configuration of Cu^I^ gives the advantage of higher luminescence quantum efficiencies as deactivation processes via d–d transitions are avoided.^12^ Moreover, copper complexes show promising thermally activated delayed fluorescence (TADF) properties for light‐emitting devices.^12^, ^13^

In spite of these advantages of Cu^I^ complexes for DSCs, there are also challenging issues concerning their suitability for the purpose. Excitation of the MLCT band results in the transfer of one electron from the HOMO of the metal ion to the π* LUMO of the ligand yielding a Cu^II^ d^9^ electron configuration. The loss of one electron results in an uneven number of electrons in the d orbitals leading to a flattening distortion of the ligands due to pseudo‐Jahn–Teller effects.^14^, ^15^, ^16^ This flattening facilitates the exciplex formation with Lewis bases (e.g. solvent molecules and/or counterions) favoring non‐radiative relaxation processes. Another drawback of complexes with Cu^I^ as a first row d^10^ transition metal is their limited structural integrity due to ligand loss by substitution.^17^, ^18^ Consequently, not all interesting Ru^II^ complexes can be mimicked by Cu^I^ analogues. So far the most successful strategy to overcome these challenges is based on bulky substituents in the HETPHEN approach^19^, ^20^, ^21^ reported by Schmittel and co‐workers. The handicaps of bulky substituents are decreased MLCT band intensities and less pronounced distortions in the excited states.^22^

Cu^I^ complexes have been used extensively to spatially organize tailor‐made ligands forming mechanically interlocked superstructures (e.g. pseudorotaxanes, rotaxanes, catenanes, and knots) upon covalent interlinking of the ligands and subsequent release of the coordinating metal ion.^23^, ^24^, ^25^, ^26^, ^27^, ^28^, ^29^ In most of these studies, the metal complexes are mainly organizing tools, in a few cases even triggered by external stimuli, and their optical features are less in the focus of interest.

**Figure 1:**
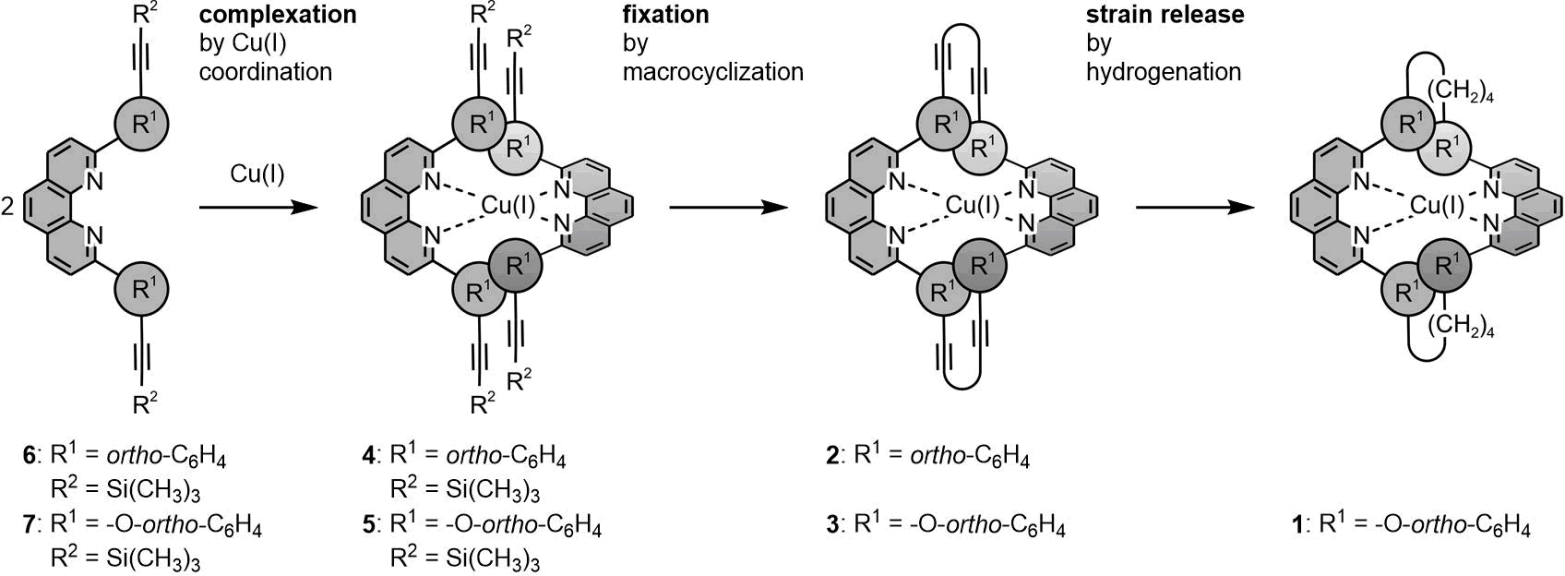
Concept to stabilize the ligand sphere by interlinking both phenanthroline ligands to a tetradentate macrocycle. To allow the macrocyclic ligand to adapt to the requirements imposed by the Cu^I^ coordination sphere, the rigid diacetylene bridges were hydrogenated to the corresponding saturated buta‐1,4‐diyl bridges in the case of target compound **1**.

**Scheme 1:**
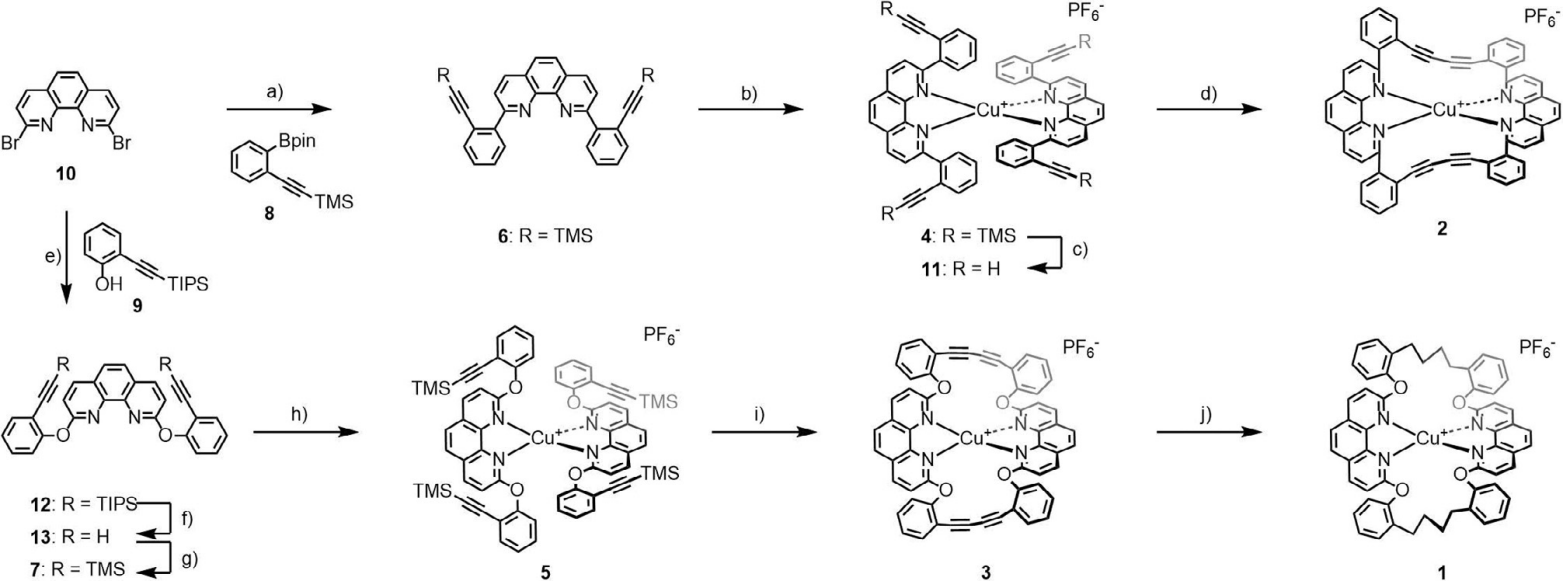
Synthesis of the copper complexes **1** and **2**: a) **8**, K_2_CO_3_, Pd(PPh_3_)_4_, toluene/H_2_O (6:1), reflux, 20 h, 52 %; b) [Cu(MeCN)_4_][PF_6_], DCM, RT, 30 min, quant.; c) K_2_CO_3_, DCM/MeOH (1:1), RT, 12 h, quant.; d) CuCl, TMEDA, DCM, RT, 5 h, quant.; e) **9**, *t*BuXPhos, K_3_PO_4_, Pd(OAc)_2_, toluene, reflux, 12 h, 72 %; f) TBAF, THF, RT, 10 min, 99 %; g) TMSCl, *n*BuLi, THF, −78 °C, 30 min, 68 %; h) [Cu(MeCN)_4_][PF_6_], DCM, RT, 30 min, quant.; i) CuCl, TMEDA, K_2_CO_3_, DCM/MeOH (1:1), RT, 1 h, 79 %; j) H_2_, Pd/C, MeOH, reflux, 20 h, 48 %.

Recently we reported an approach to interlink coordinated terpyridine (tpy) ligands to a macrocycle, not only to improve the stability of the complex,^30^ but also to provide a chiral ligand sphere.^31^ Herein, we report a similar strategy (Figure 1) to macrocyclize Cu^I^ phenantroline complexes in order to tightly fix their ligand sphere with the intention to increase their structural integrity. The macrocyclization is again based on Glaser–Hay‐type oxidative acetylene coupling,^32^ forming, similar to the approach of Heuft and Fallis,^33^ the complexes **2** and **3** with different strain upon macrocyclization. In the case of **3** the strain was further released by subsequent hydrogenation of the diacetylene bridge resulting in the buta‐1,4‐diyl interlinked bis phenanthroline Cu^I^ complex **1** (Scheme 1).

## Results and Discussion

### Molecular design

Both ligands **6** and **7** expose a trimethylsilyl (TMS)‐protected acetylene at their periphery with the intention sketched in Figure 1, namely, to enable macrocyclization of the deprotected acetylenes by oxidative acetylene coupling upon preorganization in a Cu^I^ complex. In both ligands, the 2‐ and 9‐positions of the parent phenanthroline are decorated with *ortho*‐trimethylsilylethinylphenyl substituents. While the substituents are directly coupled to the phenanthroline skeleton in **6**, an interlinking oxygen atom increases both, distance and flexibility of the substituents in **7**. The flexibility of the substituents bearing the interlinking groups is not only key for a successful macrocyclization in the complex, it is also expected to have an impact on the coordination geometry of the macrocyclized complex.

To further increase the adaptability of the ligand sphere of the macrocyclized complex, the rigid diacetylene linkers can be hydrogenated to more flexible 1,4‐butadiyl linkers.

The macrocyclized tetradentate ligand system should increase the structural integrity of the complex. In particular, the ligand dissociation in the excited state is expected to be suppressed by intramolecularly forcing all four coordination sites in proximity of the metal center. Even more interesting would be macrocyclized ligand spheres maintaining the optical properties of the parent bis‐phenanthroline Cu^I^ complex, but handicapping the planarization of the complex in the excited state by fixing the spatial arrangement of both phenanthroline subunits with respect to each other. Exciplex formation of the planarized complex is reported as a major pathway of nonradiative relaxation of the excited state, and thus, an ideal macrocyclic tetradentate ligand might even improve the emission features of the complex by avoiding this nonradiative relaxation pathway.

### Synthesis and characterization

Complexes **2** and **3** were both assembled by the same synthetic strategy: First the phenanthroline ligand exposing TMS‐protected acetylenes at both ends was synthesized and pairwise coordinated to Cu^I^. After fixation of the ligands in the complex, the exposed acetylenes were deprotected and interlinked by oxidative acetylene coupling to provide the complexes with the tetradentate macrocyclic ligand.

For the assembly of the ligands, the additionally functionalized and protected ethinylphenyl building blocks **8** and **9** were either purchased (**8**) or synthesized by following a reported procedure (**9**), which was a Sonogashira cross‐coupling reaction between triisopropylsilyl (TIPS)‐protected acetylene and 2‐iodophenol.^34^

The first ligand **6** was obtained by a twofold Suzuki reaction between two equivalents of the boronic acid derivative **8** and commercial 2,9‐dibromo‐1,10‐phenanthroline (**10**). Both reaction partners were refluxed in a toluene/water mixture (6:1) in the presence of tetrakis(triphenylphosphine)palladium [Pd(PPh_3_)_4_] as the catalyst and potassium carbonate (K_2_CO_3_) as the base. Even though K_2_CO_3_ in polar protic solvents is reported as deprotection conditions for TMS‐acetylenes, the desired ligand **6** could be isolated as white solid in 52 % yield by column chromatography.

Initial attempts to deprotect the acetylenes of **6** prior to complex formation with Cu^I^ failed, most likely due to interfering coordination of the acetylene with the copper ion. After inverting the reaction sequence, the complex **4** coordinating two ligands **6** around a Cu^I^ center was obtained by adding a degassed solution of **6** in DCM to tetrakis(acetonitrile)copper(I) hexafluorophosphate (Cu(MeCN)_4_ **⋅**PF_6_) under an argon atmosphere. The complex formation was indicated by an immediate color change to deep‐red and the desired complex **4** was isolated in quantitative yield as red solid by evaporation of the solvents.

Liberation of the alkynes was achieved quantitatively by treating **4** with K_2_CO_3_ in a 1:1 solvent mixture of DCM and methanol (MeOH) at room temperature for 12 hours, providing **11** as red solid after precipitation from acetonitrile (CH_3_CN) with aqueous ammoniumhexafluorophosphate (NH_4_PF_6_). Interlinking of both ligands by oxidative acetylene coupling to form the complex **2** with the metal center surrounded by a tetradentate macrocycle was achieved by classical Hay conditions.^35^ The complex **11** was dissolved together with CuCl and tetramethylethylendiamine (TMEDA) as the catalytic system in DCM and vigorously stirred at ambient air and temperature. Interestingly, exclusively the intramolecularly interlinked complex was observed by mass spectrometry and isolated. No dimer nor oligomer formation was observed. Thus, it seems that the increased reaction rate due to the pre‐organized proximity of the alkynes in the intramolecular coupling outperforms the competing intermolecular side reactions. The desired complex **2** was formed quantitatively but displayed limited stability features. The decomposition of the complex is accompanied by a color change from deep‐red to black, which was already faintly observed within the five hours of the macrocyclization reaction and complete decomposition of the dissolved complex was observed within a few hours. The limiting stability of **2** handicapped its characterization and not all expected ^13^C NMR signals of the complex could be detected.

**Figure 2:**
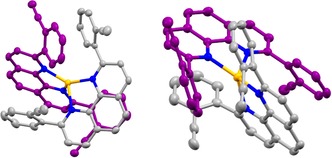
Solid‐state structure of **11** with rotation ellipsoids at 50 % probability. Hydrogen atoms and the PF_6_^−^ counterion were omitted for clarity. Color code: N: blue, Cu: yellow. The carbon atoms are colored purple and grey to differentiate both ligand systems optically. The angle between the phenanthrolines was measured to be 60.15° and Cu−N bond lengths were measured to be between 2.026 and 2.117 Å. CCDC https://www.ccdc.cam.ac.uk/services/structures?id=doi:10.1002/chem.201904754 contains the supplementary crystallographic data for this paper. These data are provided free of charge by http://www.ccdc.cam.ac.uk/.

Single crystals suitable for analyzing the solid‐state structure by X‐ray diffraction were obtained for the precursor complex (**11**) by vapor diffusion of diethylether into a solution of **11** in DCM. Interestingly a considerable strain induced distortion from the tetrahedral coordination sphere is already observed in the precursor complex, which is however still perfectly stable compared with the closed **2**. In the solid‐state structure (Figure 2), π‐stacking between one of the ethinylphenyl substituents and the phenanthroline π‐system of the other ligand are distorting the coordination sphere resulting in an angle of 60.15° between the planes defined by the π‐system of the phenanthrolines. Another eye‐catching feature of the solid‐state structure are the orientations of the peripheral ethinyl groups, which are pointing away from each other and are thus not ideally arranged to support a facile intramolecular diacetylene coupling.

The limited stability of **2** arises from the strain induced by the macrocycle destabilizing the complex. To improve the complex stability the strain has to be released. Our strategy was to introduce additional interlinking atoms between the ligand's subunits to increase both their degrees of freedom for the macrocyclization reaction and the flexibility of the macrocyclized tetradentate ligand system. As second generation ligand, the phenanthroline **7** was suggested with interlinking oxygen atoms between its TMS‐protected ethinylphenyl subunits and the phenanthroline.

To assemble **7**, commercial 2,9‐dibromo‐1,10‐phenanthroline (**10**) was reacted with 2‐[(triisopropylsilyl)ethynyl]phenol (**9**) in a Buchwald‐type reaction using a modified reported procedure,^36^ preparing in situ the catalyst with 2‐di‐*tert*‐butylphosphino‐2′,4′,6′‐triisopropylbiphenyl (*t*BuXPhos) as the ligand and palladium (II) acetate (Pd(OAc)_2_) as the metal source, using tripotassium phosphate (K_3_PO_4_) as the base in refluxing toluene for 12 hours. The TIPS‐protected ligand **12** was isolated as a yellowish oil in 72 % yield by column chromatography. Initial attempts to directly synthesize **7** by using TMS as an alkyne protection group instead of TIPS were challenging due to partial deprotection during the ligand synthesis. The lability of the Cu phenanthroline complex, however, disfavors TIPS protection groups requiring harsher reaction conditions for their removal. Thus **12** was treated with tetrabutylammonium fluoride (TBAF) in THF for 10 minutes at room temperature, providing the alkyne decorated ligand **13** as a white solid in 99 % yield after column chromatography. The TMS‐protected ligand **7** was obtained by treating **13** with trimethylsilyl chloride and n‐butyl lithium in THF at −78 °C. After warm up to room temperature and subsequent workup, the ligand **7** was isolated in 68 % yield as a yellow oil by column chromatography.

Complexation of **7** with copper was achieved by a similar protocol as already applied successfully for **6**. Using (Cu(MeCN)_4_ **⋅**PF_6_) as a copper source in DCM under inert conditions provided the red TMS‐protected complex **5** in quantitative yield.

Initial macrocyclization experiments with **5** revealed a limited stability of the liberated tetraalkyne complex and thus, an in situ deprotection–macrocyclization strategy was considered minimizing the appearance of the fully deprotected complex. CuCl and TMEDA were dissolved in a DCM/MeOH mixture (3:1) and K_2_CO_3_ was added to deprotect the alkyne groups. The reaction was vigorously stirred at room temperature while its course was monitored by direct injection electrospray ionization mass spectrometry (DI‐ESI‐MS). After 1 h, exclusively the mass expected for the closed complex **3** was recorded. The reaction was quenched and worked up to provide the macrocyclized red complex **3** in 79 % yield.

All attempts to grow single crystals from the complexes **5**, deprotected **5**, and **3** failed so far, probably reflecting the lack of stabilizing π‐stacking interactions between both ligands. However, the increased stability of **3** compared with **2** points at an improved ability of the tetradentate macrocyclic ligand of **3** to provide a Cu^I^ coordination sphere. To improve this feature further, the adaptability of the coordination sphere was enhanced by reducing the stiffness of the 1,3‐butadiyne‐1,4‐diyl bridges of **3** by converting them to the buta‐1,4‐diyl bridges of **1**. To complex **3** dissolved in MeOH, palladium on activated carbon powder was added as hydrogenation catalyst and the reaction was refluxed for 20 h under a hydrogen atmosphere. After workup, the red–orange complex **1** was isolated in 48 % yield by column chromatography. The same reaction conditions were applied to complex **2**; however, the hydrogenated complex could not be obtained.

The superior stability of **1** compared with the other Cu^I^ complexes reported in this publication became already obvious during its purification, as it was the only one not displaying degradation on silica gel. Therefore, **1** was the only complex that was fully characterized by ^1^H, ^13^C, COSY, NOESY, HMQC, and HMBC NMR spectroscopy as well as HR‐ESI‐MS.

**Scheme 2:**
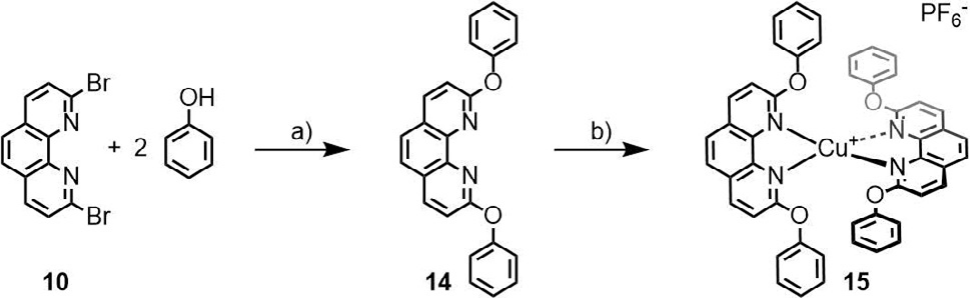
Synthesis of the reference Cu^I^ complex **15**: a) *t*BuXPhos, K_3_PO_4_, Pd(OAc)_2_, toluene, reflux, 12 h, 68 %; b) Cu(MeCN)_4_⋅PF_6_, DCM, RT, 30 min, quant.

To evaluate the effects of the macrocyclized tetradentate ligand of **1**, a structurally similar complex lacking the bridges between the phenanthrolines was required. As reference model compound the complex **15** was synthesized (Scheme 2). The 2,9‐diphenoxy‐1,10‐phenanthroline (**14**) ligand was obtained in 68 % yield after column chromatography using comparable reaction conditions as for the assembly of **12**. Coordination to Cu^I^ was achieved with similar conditions as for the synthesis of **5**, providing the complex **15** in quantitative yield.

### Spectroscopic investigations and DFT calculations

**Figure 3:**
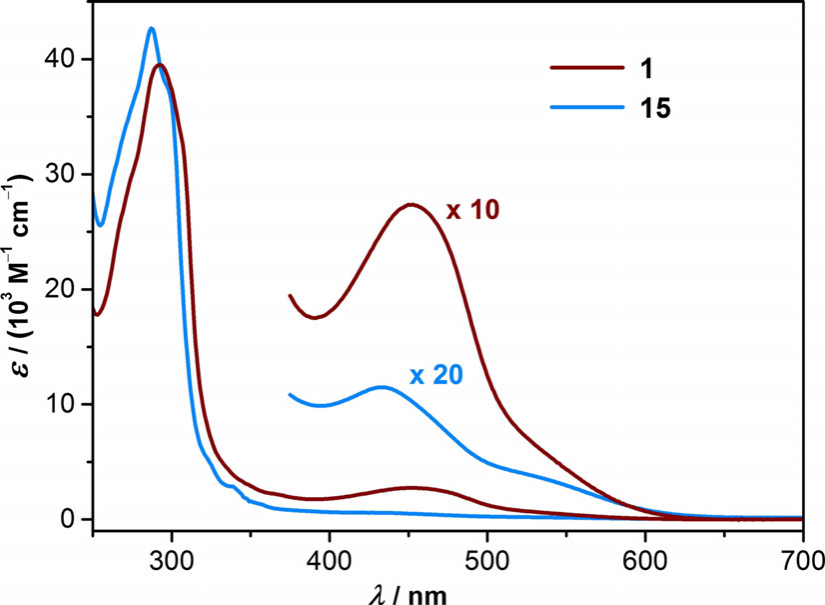
UV/Vis spectra of the macrocyclic complex **1** and the reference complex **15** measured in acetonitrile at room temperature. To improve their visibility, the MLCT absorption bands in the visible were increased by the indicated scaling factors.

Whereas the free ligands **6**, **7**, and **14** are white solids absorbing exclusively in the UV region, their Cu^I^ complexes display characteristic metal‐to‐ligand charge‐transfer (MLCT) bands around 450 nm, which are displayed in Figure 3 for the macrocyclic complex **1** and the open reference **15** recorded in CH_3_CN. Prominent absorption maxima are summarized in Table 1 with comparison to [Cu(2,9‐Me_2_phen)_2_][PF_6_], which was synthesized according to literature.^37^ Oxygen atoms directly attached to the phenanthroline unit seem to diminish the intensity of the MLCT band.^38^, ^39^ Furthermore, bulky substituents at the 2‐ and 9‐positions of phenanthroline are reported to reduce the intensities of the MLCT band in the visible region.^22^ Interestingly, the effect is much more pronounced for the open model complex **15** with a hardly visible MLCT band, while the one of the macrocyclized complex **1** is about five times more intense and clearly visible even without magnification of the spectral range.

**Table 1:** UV/Vis absorption maxima and extinction coefficients of **1**, **15**, and [Cu(Me_2_phen)_2_[PF_6_].

Compd.	*λ*_max1_ [nm] (*ϵ* [m ^−1^ cm^−1^])	*λ*_max2_ [nm] (*ϵ* [m ^−1^ cm^−1^])
**1**	292 (39 510)	452 (2740)
**15**	287 (42 690)	433 (580)
[Cu(Me_2_phen)_2_][PF_6_]	273 (45 000)	456 (5900)

**Figure 4:**
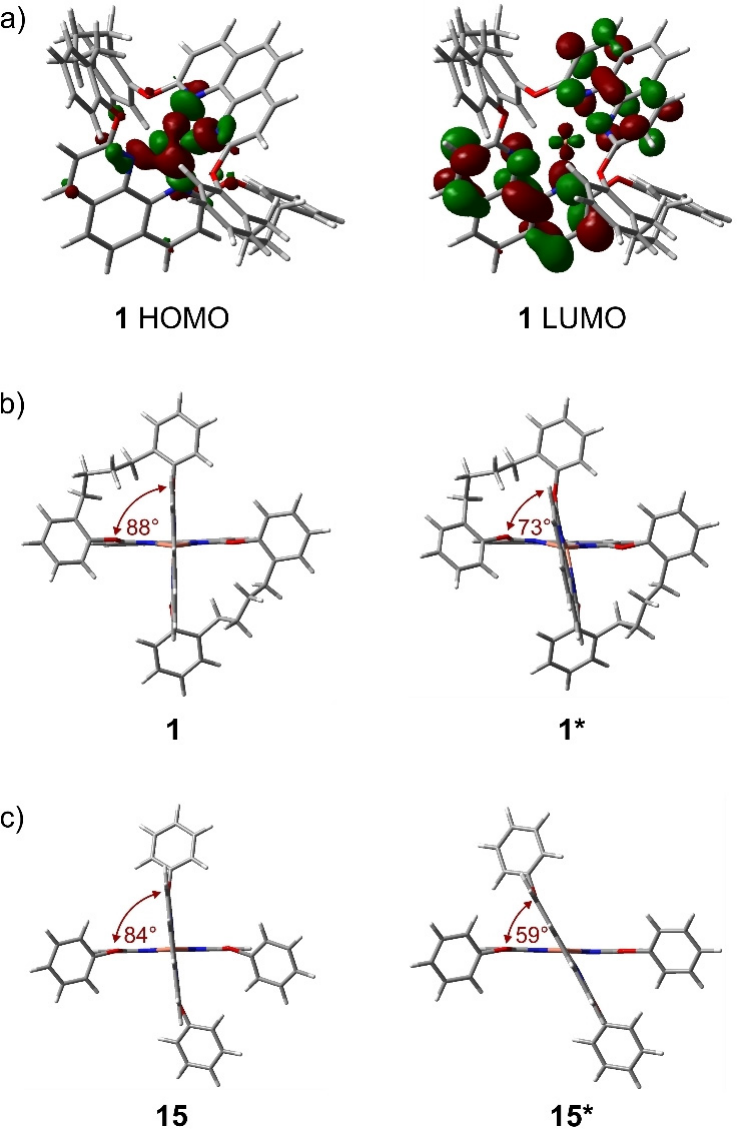
a) DFT‐optimized structure of **1** at the B3LYP/LANL2DZ level of theory displaying the computed HOMO (left) and LUMO (right) orbitals. Comparison of computed ground‐state (left) and excited‐state geometries (right) of b) the macrocyclic complex **1** and c) the reference complex **15** (bottom). The angles measured between both phenanthroline planes are given in ruby red.

The UV/Vis analysis was complemented by DFT calculations of the macrocyclized Cu^I^ complex **1**. Figure 4 a) displays the computed frontier orbitals in the DFT‐optimized structure. As reported for the parent Cu^I^ bis‐phenanthroline motive,^40^ also for **1** the HOMO is metal centered, whereas the LUMO is spread over both phenanthroline ligands. These findings are in line with the observed long‐wavelength MLCT transitions and the typical MLCT excited‐state behavior of Cu^I^ bisphenanthroline complexes.^40^ Similar calculations of the frontier orbitals of the reference complex **15** are provided in the Supporting Information.

For both Cu^I^ phenanthroline complexes, the spatially fixed **1** and the open **15**, all attempts to record room temperature emissions (steady state and time resolved) with argon saturated solutions in CH_3_CN failed. The lack of room temperature emission points at ultrashort excited‐state lifetimes, in analogy with the findings reported for sterically less shielded complexes in coordinating solvents.^40^, ^41^ The ultrashort lifetime of the excited state is likely due to the flattening process occurring by the conversion of Cu^I^ to Cu^II^ upon photoexcitation. It seems that the flexibility of the buta‐1,4‐diyl linkers not only increases the stability of the complex by releasing its strain, but also allows for considerable flattening in the excited state and is thus not able to retain the pseudotetrahedral geometry of the ground state.

Comparative DFT calculations of the singlet ground state and the triplet excited state geometries of both complexes **1** and **15** support this interpretation of the reduced lifetime of the excited state (Figure 4 b,c). While the linkers in **1** between both phenanthrolines decreases the extent of the flattening process, the structural change upon excitation is still pronounced opening an attack side for Lewis bases forming an exciplex accelerating nonradiative relaxation pathways. While the difference in the angle between both phenanthroline ligands is reduced by 15° for **1** during the transition from the ground‐ to the excited triplet state (Figure 4 b), the value calculated for the open reference complex **15** is 25° (Figure 4 c). A space‐filling sketch of **1** in the excited triplet state (Figure S2, Supporting Information) illustrates the open void around the copper ion giving access for Lewis bases.

**Figure 5:**
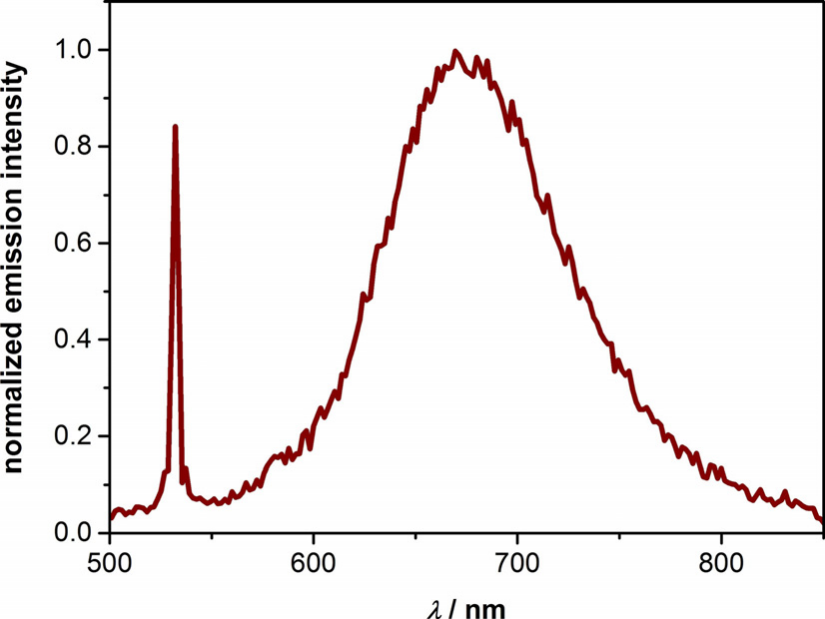
Emission spectrum of complex **1** in frozen CH_3_CN matrix at 77 K after excitation at 532 nm with laser pulses of ≈10 ns duration. The spectrum was time‐integrated over 100 ns starting with the laser pulse (pulse energy, 30 mJ). The sharp peak at 532 nm is due to stray excitation light.

While the reduced flexibility of both phenanthroline ligands in **1** compared with **15** was not enough to compete with the complex flattening at room temperature, it might be observable at lower temperature. And indeed, only the macrocyclized complex **1** displayed distinct red emission after excitation with laser pulses at 532 nm in frozen CH_3_CN at 77 K (Figure 5). Control experiments with **15** as well as the pure solvent did not show any detectable emission under these conditions.

**Figure 6:**
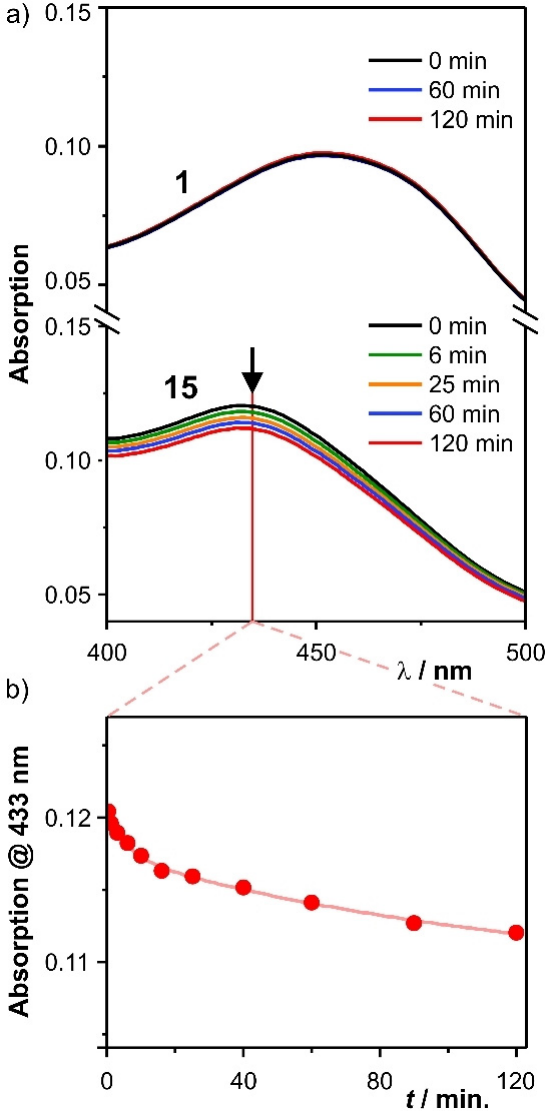
a) UV/Vis data of **1** (top) and **15** (bottom) recorded after different periods of LED illumination (455 nm, 500 mW optical output) to investigate photostability. b) Development of the absorption of **15** at 433 nm during the experiment.

After demonstrating improved emission features of the “closed” complex **1**, the key question whether the macrocyclization improves the compound's photostability remained. Both complexes **1** and **15** are stable in CH_3_CN stored in the dark. Their photostabilities were investigated by illuminating samples with a 455 nm LED source in the UV/Vis spectrometer (a detailed description is provided in the Supporting Information). The concentration of the complexes under investigation was adjusted such that both samples have similar absorption at 455 nm to guarantee comparable light absorption of both samples and to avoid heating. While the UV spectrum of the complex **1** with the macrocyclic tetradentate ligand did not alter within 2 h, the open model complex **15** displayed substantial degradation exposed to similar conditions. In Figure 6 a) UV spectra at different points in time of the MLCT band region of both complexes are displayed. The decrease of the MLCT band of **15** is visible by the bare eye and within the 2 h experiment, its intensity is decreased by about 7 %. Figure 6 b) displays the development of the band's intensity during the experiment. The obtained value (decrease of 7 % in 2 h), however, has to be handled carefully, as information concerning the UV/Vis absorption features of the complex's decomposition products are missing. It is noteworthy that the experimental setup to analyse the photostability has already been applied recently for the characterization of acridinium photocatalysts.^42^

The available data display the improved photostability of the macrocyclized ligand disfavouring ligand dissociation by arranging all four required coordination sites around the central copper ion.

Redox properties of the complexes **1**, **2**, **3**, **4**, **5**, and **15** were analyzed by cyclic voltammetry (CV) and square wave voltammetry (SWV) to complement the analytical data (see Supporting Information). While the expected reduction of the oxidation potential with increasing electron density on the phenanthroline ligand was observed for example, by the introduction of the oxygen atom in the comparison between **4** (1.00 V vs. Ag/AgCl/3 m KCl) and **5** (0.70 V vs. Ag/AgCl/3 m KCl), the effects of the ligand fixation by macrocyclization were less conclusive. The already above mentioned limited stability of the strained macrocycles **3** and **2** further handicapped the electrochemical analyses. Interestingly, very comparable oxidation potentials were recorded for the complex **1** (1.01 V vs. Ag/AgCl/3 m KCl) and the model compound **15** (1.00 V vs. Ag/AgCl/3 m KCl), suggesting comparable spatial arrangements and flexibilities of the phenanthroline ligands in both complexes.

## Conclusion

A new concept to stabilize Cu^I^ bis(phenanthroline) complexes is presented. By interlinking of the coordinating phenanthroline ligands a tetradentate macrocycle is obtained with the rationale to obtain complexes displaying superior stability due to increased chelate properties. The macrocyclization is achieved by oxidative acetylene coupling of suitably functionalized phenanthroline ligands. The obtained Cu^I^ complexes with macrocyclic ligands (**2** and **3**) display limited stability due to mismatch of the ligand fixation in the macrocycle and the geometrical requirements of the metal center. Hydrogenation of the interlinking diacetylenes provides a more flexible macrocycle with improved adaptability to the coordination requirements and consequently the perfectly stable complex **1**. The macrocylized complex displayed not only improved photostability, but also light emission; however, only at low temperature.

To enable room‐temperature emission the flattening of the complex should be avoided, requiring either a stiffer macrocycle satisfying the metal coordination sphere or additional linkers between the ligands resulting in a three‐dimensional network as coordination cage surrounding the central metal ion. Both approaches are appealing and in the focus of our current developments.

## Experimental Section

### General procedures

All commercially available chemicals were used without further purification. Dry solvents were used as crown cap and purchased from Acros Organics and Sigma–Aldrich. NMR solvents were obtained from CIL Cambridge Isotope Laboratories, Inc. (Andover, MA, USA) or Sigma–Aldrich. All NMR experiments were performed on Bruker Avance III or III HD, two or four‐channel NMR spectrometer operating at 400.13 or 500.13 MHz proton frequency. The instruments were equipped with direct observe BBFO, indirect BBI or cryogenic four‐channel QCI (H/C/N/F) 5 mm probes all with self‐shielded *z*‐gradient. The experiments were performed at 298 or 295 K. All chemical shifts (*δ*) are reported in parts per million (ppm) relative to the used solvent and coupling constants, (*J*) are given in Hertz (Hz). The multiplicities are written as: s=singlet, d=doublet, dd=doublet of doublet, td=triplet of doublet, ddd=doublet of doublet of doublet m=multiplet. Column chromatography was performed with SiliaFlash® P60 from SILICYCLE with a particle size of 40–63 μm (230–400 mesh) and for TLC Silica gel 60 F_254_ glass plates with a thickness of 0.25 mm from Merck were used. The detection was observed with a UV‐lamp at 254 or 366 nm. Gel permeation chromatography (GPC) was performed on a Shimadzu Prominence System with PSS SDV preparative columns from PSS (2 columns in series: 600 mm×20.0 mm, 5 μm particles, linear porosity “S”, operating ranges: 100–100 000 g mol^−1^) using chloroform as solvent. HRMS were measured as HR‐ESI‐ToF‐MS with a Maxis 4G instrument from Bruker.

### Optical spectroscopy

Steady‐state absorption and emission spectra were recorded using a Cary 5000 spectrophotometer (Varian) and a Fluorolog‐3–22 instrument (Horiba Jobin–Yvon), respectively. Emission spectra and kinetic emission traces with pulsed laser excitation (excitation wavelengths, 450 nm or 532 nm) were recorded with an LP920‐KS spectrometer from Edinburgh Instruments. A detailed description of that setup has been given previously.^43^ All solutions prepared for optical spectroscopy were deoxygenated with argon (4.8, PanGas). Details concerning the photostability measurements are given in the Supporting Information.

### DFT calculations

The DFT calculations on **1** and **15** were carried out using the Gaussian 09 software package.^44^ Both structures were optimized with the B3LYP functional and the LANL2DZ basis set, which is well‐suited for computations of copper complexes^12^, ^45^ Triplet‐state calculations were carried out with the starting geometry of the corresponding optimized singlet ground state structure. Additional frequency analyses of all optimized structures did not produce negative frequencies, indicating convergence on minimum structures.

### 2,9‐Bis{2‐[(trimethylsilyl)ethynyl]phenyl}‐1,10‐phenanthroline (6)

A 100 mL round‐bottomed flask was set under an argon atmosphere and was charged with 2,9‐dibromo‐1,10‐phenanthroline (500 g, 1.48 mmol, 1.0 equiv), trimethyl((2‐(4,4,5,5‐tetramethyl‐1,3,2‐dioxaborolan‐2‐yl)phenyl)ethynyl)silane (1.37 mg, 4.44 mmol, 3.0 equiv), K_2_CO_3_ (413 mg, 2.96 mmol, 2.0 equiv), tetrakis(triphenylphosphine)palladium(0) (85.5 mg, 74.0 μmol, 5 mol %) and the solvents toluene/water (60 mL, 6:1). The reaction mixture was degassed for 20 minutes with an argon stream before it was heated to reflux for 20 hours. The reaction was stopped, plugged over Silica (ethyl acetate), concentrated under reduced pressure and purified by column chromatography (cyclohexane to cyclohexane/ethyl acetate=9:1) to obtain the product as white solid (407 mg, 776 μmol, 52 %).

### Analytical data for 6

^1^H NMR (400 MHz, CDCl_3_, 22 °C): *δ*=8.46 (d, *J*=8.4 Hz, 2 H), 8.26 (d, *J*=8.4 Hz, 2 H), 8.24 (dd, *J*=7.8, 1.3 Hz, 2 H), 7.87 (s, 2 H), 7.61 (dd, *J*=7.9, 1.3 Hz, 2 H), 7.52 (td, *J*=7.6, 1.4 Hz, 2 H), 7.38 (td, *J*=7.5, 1.4 Hz, 2 H), 0.13 ppm (s, 18 H); ^13^C NMR (126 MHz, CDCl_3_, 25 °C): *δ*=157.68, 146.22, 143.06, 135.23, 133.46, 131.08, 129.21, 128.47, 127.90, 126.45, 124.63, 121.63, 105.10, 98.23, −0.16 ppm; HRMS (ESI‐ToF): calcd for [C_34_H_33_N_2_Si_2_]^+^ 525.2177; found: 525.2181.

### Copper(I) complex of 2,9‐bis{2‐[(trimethylsilyl)ethynyl]phenyl}‐1,10‐phenanthroline (4)

A 10 mL nitrogen‐flushed round‐bottomed flask was charged with [Cu(MeCN)_4_][PF_6_] (8.11 mg, 21.1 μmol, 1.0 equiv). To the copper salt was added 2,9‐bis{2‐[(trimethylsilyl)ethynyl]phenyl}1,10‐phenanthroline (**6**, 22.1 mg, 42.2 μmol, 2.0 equiv) dissolved in a nitrogen saturated solution of DCM (10 mL). The solution immediately turned dark‐red and the reaction mixture was stirred at room temperature for 30 min. The solution was concentrated under reduced pressure to obtain the product as red solid (23.6 mg, 21.0 μmol, 100 %).

### Analytical data for 4

^1^H NMR (400 MHz, CD_2_Cl_2_, 22 °C): *δ*=8.50 (d, *J=*8.3 Hz, 4 H), 8.16 (d, *J=*8.4 Hz, 4 H), 8.08 (s, 4 H), 7.11 (dd, *J=*7.8, 0.8 Hz, 4 H), 7.06 (dd, *J=*7.7, 0.8 Hz, 4 H), 6.72 (td, *J=*7.7, 1.3 Hz, 4 H), 6.23 (td, *J=*7.6, 1.3 Hz, 4 H), 0.05 ppm (s, 36 H); ^13^C NMR (101 MHz, CD_2_Cl_2_, 22 °C): *δ*=156.91, 143.88, 141.66, 136.46, 132.95, 129.25, 129.08, 128.98, 127.85, 127.72, 127.35, 121.74, 103.54, 100.26, −0.23 ppm; HRMS (ESI‐ToF): calcd for [C_68_H_64_CuN_4_Si_4_]^+^ 1111.3499; found: 1111.3490.

### Copper(I) complex of 2,9‐bis(2‐ethynylphenyl)‐1,10‐phenanthroline (11)

A 100 mL nitrogen‐flushed round‐bottomed flask was charged with the copper(I) complex of 2,9‐bis{2‐[(trimethylsilyl)ethynyl]phenyl}‐1,10‐phenanthroline (**4**, 46.0 mg, 36.6 μmol, 1.0 equiv) and the solvents DCM/MeOH (10 mL, 1:1). The reaction mixture was degassed with an argon stream for 20 min. To the copper complex was added K_2_CO_3_ (20.2 mg, 146 μmol, 4.0 equiv) and the reaction mixture was stirred at room temperature for 12 h. The solution was concentrated under reduced pressure. The residue was dissolved in a minimum of MeCN and the complex was precipitated by addition of a saturated aqueous NH_4_PF_6_ solution. The precipitate was filtered over millipore and concentrated under reduced pressure to obtain the product as red solid (35.4 mg, 36.6 μmol, 100 %).

### Analytical data for 11

^1^H NMR (400 MHz, CD_2_Cl_2_, 22 °C): *δ*=8.53 (d, *J=*8.3 Hz, 4 H), 8.10 (d, *J=*8.4 Hz, 4 H), 8.08 (s, 4 H), 7.14 (td, *J=*8.2, 1.3 Hz, 8 H), 6.79 (td, *J=*7.6, 1.4 Hz, 4 H), 6.32 (td, *J=*7.6, 1.3 Hz, 4 H), 3.05 ppm (s, 4 H); ^13^C NMR (101 MHz, CD_2_Cl_2_, 22 °C) *δ*=156.66, 143.94, 141.82, 136.99, 133.34, 129.32, 129.05, 129.04, 127.92, 127.66, 127.44, 120.62, 82.72, 82.29 ppm; HRMS (ESI‐ToF): calcd for [C_56_H_32_CuN_4_]^+^: 823.1917; found: 823.1905.

### Homocoupled copper(I) complex of 2,9‐bis(2‐ethynylphenyl)‐1,10‐phenanthroline (2)

A 100 mL round‐bottomed flask was charged with DCM (20 mL), CuCl (61.0 mg, 616 μmol, 85 equiv) and TMEDA (93.9 μL, 616 μmol, 85 equiv). The reaction mixture was stirred at ambient atmosphere. The previously formed copper complex (**11**, 6.00 mg, 7.28 μmol, 1.0 equiv) was dissolved in DCM (10 mL) and added slowly to the reaction mixture. The solution was stirred for 5 h at ambient atmosphere. The solution was concentrated under reduced pressure. The residue was dissolved in a minimum of MeCN and the complex was precipitated by addition of a saturated aqueous NH_4_PF_6_ solution. The precipitate was filtered over millipore and concentrated under reduced pressure to obtain the product as red solid (35.4 mg, 36.6 μmol, 100 %).

### Analytical data for 2

^1^H NMR (400 MHz, CD_2_Cl_2_, 22 °C): *δ*=8.42 (d, *J=*8.4 Hz, 4 H), 7.99 (d, *J=*8.6 Hz, 6 H), 7.95 (d, *J=*6.7 Hz, 6 H), 7.66 (dd, *J=*7.7, 1.5 Hz, 4 H), 7.59 (td, *J=*7.7, 1.5 Hz, 4 H), 7.49 ppm (td, *J=*7.5, 1.2 Hz, 4 H); ^13^C NMR (126 MHz, CD_2_Cl_2_, 25 °C): *δ*=157.29, 146.10, 142.13, 137.08, 131.80, 130.92, 129.23, 128.39, 126.99, 124.42, 120.87, 92.43 ppm. Two carbon atoms could not be assigned due to low concentrations and relatively fast decomposition. The other carbon atoms were assigned by ^13^C‐ and HMBC‐NMR. HRMS (ESI‐ToF): calcd for [C_56_H_28_CuN_4_]^+^ 819.1604; found: 819.1599.

### 2,9‐Bis{2‐[(triisopropylsilyl)ethynyl]phenoxy}‐1,10‐phenanthroline (12)

A 100 mL round‐bottomed flask was charged with 2,9‐dibromo‐1,10‐phenanthroline (800 mg, 2.25 mmol, 1.0 equiv), 2‐[(triisopropylsilyl)ethynyl]phenol (1.37 g, 4.97 mmol, 2.2 equiv), *t*BuXPhos (96.0 mg, 226 μmol, 10 mol %), Pd(OAc)_2_ (25.4 mg, 113 μmol, 5 mol %), and K_3_PO_4_ (1.92 g, 9.04 mmol, 4.0 equiv). The reaction flask was evacuated and back filled with argon for three times, before dry degassed toluene (50 mL) was added. The reaction mixture was heated to reflux and was stirred at this temperature for 12 hours. After complete conversion to the product, the reaction was stopped and filtered over a pad of silica. The crude product was purified by column chromatography (cyclohexane/ethyl acetate=9:1) and GPC (chloroform) to obtain the product as slightly yellow oil (1.17 g, 1.62 mmol, 72 %).

### Analytical data for 12

^1^H NMR (400 MHz, CD_2_Cl_2_, 22 °C): *δ*=8.19 (d, *J=*8.6 Hz, 2 H), 7.68 (s, 2 H), 7.52 (dd, *J=*7.7, 1.7 Hz, 2 H), 7.41 (dd, *J=*8.2, 1.2 Hz, 2 H), 7.31 (ddd, *J=*8.2, 7.4, 1.8 Hz, 2 H), 7.24 (d, *J=*8.6 Hz, 2 H), 7.19 (td, *J=*7.5, 1.2 Hz, 2 H), 0.91 ppm (s, 42 H); ^13^C NMR (101 MHz, CD_2_Cl_2_, 22 °C): *δ*=161.96, 155.20, 144.02, 140.16, 134.29, 130.34, 126.77, 125.02, 124.53, 122.84, 117.57, 114.03, 103.10, 96.23, 18.82, 11.72 ppm; HRMS (ESI‐ToF): calcd for [C_46_H_57_N_2_O_2_Si_2_]^+^: 725.3953; found: 725.3948.

### 2,9‐Bis(2‐ethynylphenoxy)‐1,10‐phenanthroline (13)

A 25 mL round‐bottomed flask was charged with 2,9‐bis{2‐[(triisopropylsilyl)ethynyl]phenoxy}‐1,10‐phenanthroline (**12**, 200 mg, 276 μmol, 1.0 equiv) and dry THF (10 mL). The solution was degassed with an argon stream for 10 min before TBAF (1 m in THF, 180 μL, 607 μmol, 2.2 equiv) was added. After 10 min, complete deprotection was indicated by DI‐ESI‐MS, the reaction was stopped, plugged over Silica (ethyl acetate) and concentrated under reduced pressure. The crude was further purified by biobeads SX‐3 (DCM) and washing with small amounts of Et_2_O to obtain the product as a white solid (113 mg, 274 μmol, 99 %).

### Analytical data for 13

^1^H NMR (400 MHz, CD_2_Cl_2_, 22 °C) *δ*=8.24 (d, *J=*8.6 Hz, 2 H), 7.72 (s, 2 H), 7.58–7.54 (m, 2 H), 7.39–7.31 (m, 4 H), 7.27 (d, *J=*8.6 Hz, 2 H), 7.22 (ddd, *J=*7.7, 6.4, 2.2 Hz, 2 H), 3.14 ppm (s, 2 H); ^13^C NMR (101 MHz, CD_2_Cl_2_, 22 °C) *δ*=162.17, 155.72, 143.98, 140.46, 134.41, 130.80, 126.85, 125.26, 124.79, 122.72, 116.18, 113.93, 82.25, 79.82 ppm; HRMS (ESI‐ToF): calcd for [C_28_H_17_N_2_O_2_]^+^: 413.1285; found: 413.1285.

### 2,9‐Bis{2‐[(trimethylsilyl)ethynyl]phenoxy}‐1,10‐phenanthroline (7)

A 100 mL round‐bottomed flask was charged with 2,9‐bis(2‐ethynylphenoxy)‐1,10‐phenanthroline (**13**, 100 mg, 242 μmol, 1.0 equiv) and dry THF (50 mL). The solution was cooled to −78 °C. Then TMSCl (1.21 mL, 1.21 mmol, 5.0 equiv) was added before *n*BuLi (1.6 m in hexane, 333 μL, 532 μmol, 2.2 equiv) was added dropwise. The reaction mixture was stirred for 30 minutes at −78 °C. Then the reaction mixture was allowed to warm up to room temperature, was quenched with water and the aqueous phase was extracted with DCM. The combined organic phases were dried over MgSO_4_, filtered, and concentrated under reduced pressure. The crude product was purified by column chromatography (cyclohexane/ethyl acetate=20:1) to obtain the product as slightly yellow oil (92.0 mg, 165 μmol, 68 %).

### Analytical data for 7

^1^H NMR (500 MHz, CD_2_Cl_2_, 25 °C): *δ*=8.23 (d, *J=*8.6 Hz, 2 H), 7.71 (s, 2 H), 7.47 (ddd, *J=*7.7, 1.7, 0.6 Hz, 2 H), 7.37–7.29 (m, 4 H), 7.27 (d, *J=*8.6 Hz, 2 H), 7.19 (ddd, *J=*7.7, 7.1, 1.6 Hz, 2 H), −0.04 ppm (s, 18 H); ^13^C NMR (126 MHz, CD_2_Cl_2_, 25 °C): *δ*=162.29, 155.67, 143.97, 140.22, 133.88, 130.54, 126.75, 125.20, 124.63, 122.93, 117.21, 114.12, 101.02, 100.10, −0.13 ppm; HRMS (ESI‐ToF): calcd for [C_34_H_33_N_2_O_2_Si_2_]^+^: 557.2075; found: 557.2074.

### Copper(I) complex of 2,9‐bis{2‐[(trimethylsilyl)ethynyl]phenoxy}‐1,10‐phenanthroline (5)

A 25 mL argon flushed round‐bottomed flask was charged with [Cu(MeCN)_4_][PF_6_] (26.9 mg, 70.0 μmol, 1.0 equiv). To the copper salt was added 2,9‐bis{2‐[(trimethylsilyl)ethynyl]phenoxy}‐1,10‐phenanthroline (**7**, 78.0 mg, 140 μmol, 2.0 equiv) dissolved in an argon saturated solution of DCM (10 mL). The solution immediately turned dark red and the reaction mixture was stirred at room temperature for 30 min. The solution was concentrated under reduced pressure to obtain the product as red solid (92.5 mg, 7.00 μmol, 100 %).

### Analytical data for 5

^1^H NMR (400 MHz, CD_2_Cl_2_, 22 °C): *δ*=8.40 (d, *J=*8.7 Hz, 4 H), 7.90 (s, 4 H), 7.30 (d, *J=*8.7 Hz, 4 H), 7.03 (dd, *J=*7.7, 1.7 Hz, 4 H), 6.76 (ddd, *J=*8.3, 7.4, 1.7 Hz, 4 H), 6.65 (dd, *J=*8.3, 1.2 Hz, 4 H), 6.55 (td, *J=*7.6, 1.2 Hz, 4 H), −0.10 ppm (s, 36 H); ^13^C NMR (101 MHz, CD_2_Cl_2_, 22 °C): *δ*=160.52, 154.46, 142.11, 140.84, 134.66, 130.12, 126.84, 125.46, 124.81, 120.13, 116.86, 115.71, 101.17, 100.18, −0.14 ppm; HRMS (ESI‐ToF): calcd for [C_68_H_64_CuN_4_O_4_Si_4_]^+^: 1175.3295; found: 1175.3286.

### Homocoupled copper(I) complex of 2,9‐bis(2‐ethynylphenoxy)‐1,10‐phenanthroline (3)

Due to the low stability of the interlinked complexes, the deprotection and homocoupling was performed in one pot. Therefore, a 50 mL round‐bottomed flask was charged with CuCl (159 mg, 1.60 mmol, 85 equiv), TMEDA (244 μL, 1.60 mmol, 85 equiv), and DCM (10 mL). The reaction mixture was stirred vigorously at ambient atmosphere, to saturate the solution with oxygen. Then, the copper(I) complex of 2,9‐bis{2‐[(trimethylsilyl)ethynyl]phenoxy}‐1,10‐phenanthroline (**5**, 25 mg, 18.9 μmol, 1.0 equiv) dissolved in DCM (5 mL), K_2_CO_3_ (500 mg, 3.62 mmol, 192 equiv) and MeOH (5 mL) were added. The reaction mixture was stirred at room temperature for 1 h. After DI‐ESI‐MS indicated full conversion to the closed complex, the reaction was stopped, and water was added. The organic phase was washed with water (3×), was dried over MgSO_4_, filtered and concentrated to yield the product as red solid (15.4 mg, 15.0 μmol, 79 %).

### Analytical data for 3

^1^H NMR (500 MHz, CD_2_Cl_2_, 25 °C): *δ*=8.38 (d, *J=*8.9 Hz, 4 H), 7.84 (s, 4 H), 7.60 (ddd, *J=*8.2, 7.5, 1.7 Hz, 4 H), 7.44 (ddd, *J=*7.6, 1.7, 0.5 Hz, 4 H), 7.35–7.30 (m, 8 H), 6.96 ppm (d, *J=*8.9 Hz, 4 H); ^13^C NMR (126 MHz, CD_2_Cl_2_, 25 °C): *δ*=162.34, 156.59, 143.05, 141.18, 133.27, 132.19, 127.45, 126.62, 125.07, 123.23, 116.68, 111.62, 80.54, 79.05 ppm; HRMS (ESI‐ToF): calcd for [C_56_H_28_CuN_4_O_4_]^+^: 883.1401; found: 883.1414.

### Copper(I) complex of 2,4,11,13‐tetraoxa‐3,12(2,9)‐diphenanthrolina‐1,5,10,14(1,2)‐tetrabenzenacyclooctadecaphane (1)

A dry, argon flushed 50 mL two necked flask was charged with the copper complex (**3**, 10.0 mg, 9.71 μmol, 1.0 equiv) and a spatula tips of Pd/C (5 % on activated carbon powder, standard, unreduced, nominally 50 %, water wet), before dry MeOH (10 mL) was added. The reaction mixture was degassed with argon and afterwards with hydrogen, keeping hydrogen atmosphere. The reaction mixture was heated to reflux for 20 hours. The crude product was purified by column chromatography (DCM + 2 % MeOH) and precipitation from Et_2_O to yield the product as red solid (8.10 mg, 8.00 μmol, 48 %).

### Analytical data for 1

^1^H NMR (500 MHz, CD_2_Cl_2_, 25 °C): *δ*=8.37 (d, *J=*8.9 Hz, 4 H), 7.84 (s, 4 H), 7.28 (ddd, *J=*7.9, 5.7, 3.4 Hz, 4 H), 7.24–7.16 (m, 8 H), 7.11 (d, *J=*7.8 Hz, 3 H), 7.03 (d, *J=*8.8 Hz, 4 H), 2.24 (ddd, *J=*14.4, 10.3, 4.7 Hz, 4 H), 2.11–2.01 (m, 4 H), 1.54–1.44 (m, 4 H), 1.16 ppm (ddd, *J=*9.4, 6.6, 4.3 Hz, 4 H); ^13^C NMR (126 MHz, CD_2_Cl_2_, 25 °C): *δ*=162.37, 151.43, 142.94, 141.12, 135.75, 131.55, 128.48, 127.41, 126.33, 124.91, 122.62, 112.12, 29.92, 29.16 ppm; HRMS (ESI‐ToF): calc. for [C_56_H_44_CuN_4_O_4_]^+^ 899.2653; found 899.2653.

### 2,9‐Diphenoxy‐1,10‐phenanthroline (14)

A 25 mL round‐bottomed flask was charged with 2,9‐dibromo‐1,10‐phenanthroline (150 mg, 422 μmol, 1.0 equiv), phenol (99.5 mg, 1.06 mmol, 2.5 equiv), *t*BuXPhos (18.0 mg, 42.3 μmol, 10 mol %), Pd(OAc)_2_ (4.75 mg, 21.2 μmol, 5 mol %), and K_3_PO_4_ (359 mg, 1.69 mmol, 4.0 equiv). The reaction flask was evacuated and back filled with argon for three times, before dry degassed toluene (10 mL) was added. The reaction mixture was heated to reflux and was stirred at this temperature for 12 h. After complete conversion to the product, the reaction was stopped and filtered over a pad of silica. The crude product was purified by column chromatography (cyclohexane/ethyl acetate=9:1) to obtain the product as white solid (105 mg, 288 μmol, 68 %).

### Analytical data for 14

^1^H NMR (400 MHz, CDCl_3_, 22 °C) *δ*=8.19 (d, *J=*8.6 Hz, 2 H), 7.68 (s, 2 H), 7.40–7.29 (m, 8 H), 7.24–7.17 ppm (m, 4 H); ^13^C NMR (101 MHz, CD_2_Cl_2_, 22 °C): *δ*=162.06, 154.13, 143.69, 139.83, 129.64, 126.19, 124.55, 124.23, 121.17, 113.88 ppm; HRMS (ESI‐ToF): calcd for [C_24_H_17_N_2_O_2_]^+^: 365.1285; found: 365.1289.

### Copper(I) complex of 2,9‐diphenoxy‐1,10‐phenanthroline (15)

A 25 mL argon flushed round‐bottomed flask was charged with [Cu(MeCN)_4_][PF_6_] (2.64 mg, 6.86 μmol, 1.0 equiv). To the copper salt was added 2,9‐diphenoxy‐1,10‐phenanthroline (**14**, 5.00 mg, 13.7 μmol, 2.0 equiv) dissolved in an argon saturated solution of DCM (10 mL). The solution immediately turned dark red and the reaction mixture was stirred at room temperature for 30 min. The solution was concentrated under reduced pressure to obtain the product as red solid (6.43 mg, 6.86 μmol, 100 %).

### Analytical data for 15

^1^H NMR (400 MHz, CD_2_Cl_2_, 22 °C): *δ*=8.43 (d, *J=*8.7 Hz, 4 H), 7.91 (s, 4 H), 7.29 (d, *J=*8.7 Hz, 4 H), 6.94–6.87 (m, 8 H), 6.77–6.69 (m, 4 H), 6.66–6.59 ppm (m, 8 H); ^13^C NMR (101 MHz, CD_2_Cl_2_, 22 °C): *δ*=160.31, 153.69, 141.95, 140.47, 129.47, 126.20, 124.78, 124.55, 118.90, 115.06 ppm; HRMS (ESI‐ToF): calcd for [C_48_H_32_CuN_4_O_4_]^+^: 791.1714; found: 791.1724.

## Conflict of interest

The authors declare no conflict of interest.

## Supporting information

As a service to our authors and readers, this journal provides supporting information supplied by the authors. Such materials are peer reviewed and may be re‐organized for online delivery, but are not copy‐edited or typeset. Technical support issues arising from supporting information (other than missing files) should be addressed to the authors.

Supplementary
