# Supplementary material for: Improved Photostability of a CuI Complex by Macrocyclization of the Phenanthroline Ligands
Source: Chemistry. 2020 Feb 18;26(14):3119–28. doi: 10.1002/chem.201904754 (PMC7079024; doi:10.1002/chem.201904754)
Supplement: Supplementary file 1 — Supplementary [file CHEM-26-3119-s001.pdf]

CHEMISTRY
A European Journal

Supporting Information

Improved Photostability of a Cu^{I} Complex by Macrocyclization of the Phenanthroline Ligands

Thomas Brandl,[a] Christoph Kerzig,[a] Loïc Le Pleux,[a] Alessandro Prescimone,[a]
Oliver S. Wenger,[a] and Marcel Mayor*[a, b, c]

chem_201904754_sm_miscellaneous_information.pdf

Contents

^{1}H-, ^{13}C-NMR ($CDCl_3$, 400/101 MHz, 22 °C) and HR-ESI-MS spectra of compound (**6**) 2

^{1}H-, ^{13}C-NMR (CD_2Cl_2, 400/101 MHz, 22 °C) and HR-ESI-MS spectra of compound (**4**)..... 6

^{1}H-, ^{13}C-NMR (CD_2Cl_2, 400/101 MHz, 22 °C) and HR-ESI-MS spectra of compound (**11**) 10

^{1}H-, ^{13}C-, HMBC-NMR (CD_2Cl_2, 400/126 MHz, 22/25 °C) and HR-ESI-MS spectra of compound (**2**) 14

^{1}H-, ^{13}C-NMR (CD_2Cl_2, 400/101 MHz, 22 °C) and HR-ESI-MS spectra of compound (**12**) 19

^{1}H-, ^{13}C-NMR (CD_2Cl_2, 400/101 MHz, 22 °C) and HR-ESI-MS spectra of compound (**13**) 23

^{1}H-, ^{13}C-NMR (CD_2Cl_2, 500/126 MHz, 25 °C) and HR-ESI-MS spectra of compound (**7**)... 27

^{1}H-, ^{13}C-NMR (CD_2Cl_2, 400/101 MHz, 22 °C) and HR-ESI-MS spectra of compound (**5**)... 31

^{1}H-, ^{13}C-NMR (CD_2Cl_2, 500/126 MHz, 25 °C) and HR-ESI-MS spectra of compound (**3**) .. 35

^{1}H-, ^{13}C-NMR ($CDCl_3$, 400/101 MHz, 22 °C) and HR-ESI-MS spectra of compound (**14**) . 39

^{1}H-, ^{13}C-NMR (CD_2Cl_2, 400/101 MHz, 22 °C) and HR-ESI-MS spectra of compound (**15**) 43

^{1}H-, ^{13}C-, COSY-, NOESY-, HMQC-, HMBC-NMR (CD_2Cl_2, 500/126 MHz, 22 °C) and HR-ESI-MS spectra and full assignment of compound (**1**) 47

Computational investigations 54

Crystal data for **11** 55

Photostability investigations 56

Electrochemical investigations 82

^{1}H-, ^{13}C-NMR ($CDCl_3$, 400/101 MHz, 22 °C) and HR-ESI-MS spectra of compound (6)

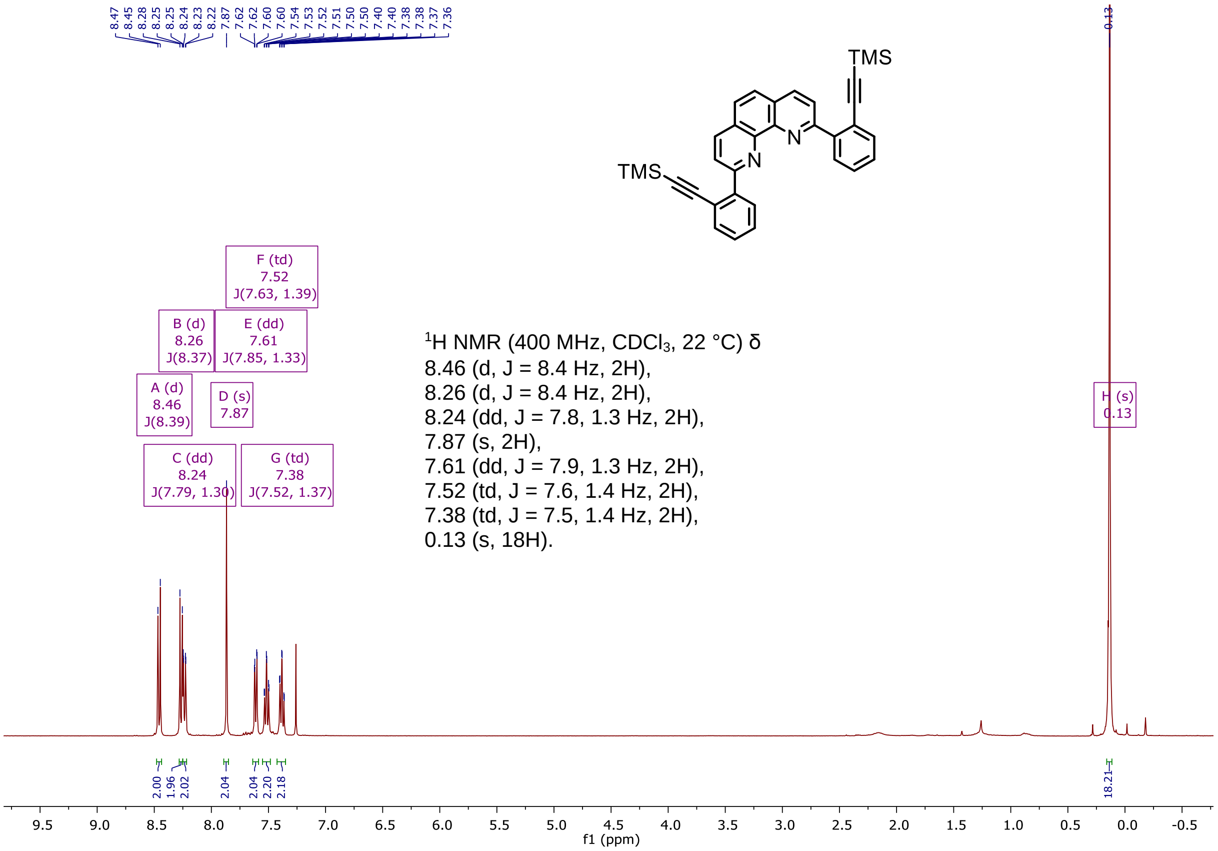

^{1}H NMR (400 MHz, $CDCl_3$, 22 °C) δ
8.46 (d, J = 8.4 Hz, 2H),
8.26 (d, J = 8.4 Hz, 2H),
8.24 (dd, J = 7.8, 1.3 Hz, 2H),
7.87 (s, 2H),
7.61 (dd, J = 7.9, 1.3 Hz, 2H),
7.52 (td, J = 7.6, 1.4 Hz, 2H),
7.38 (td, J = 7.5, 1.4 Hz, 2H),
0.13 (s, 18H).

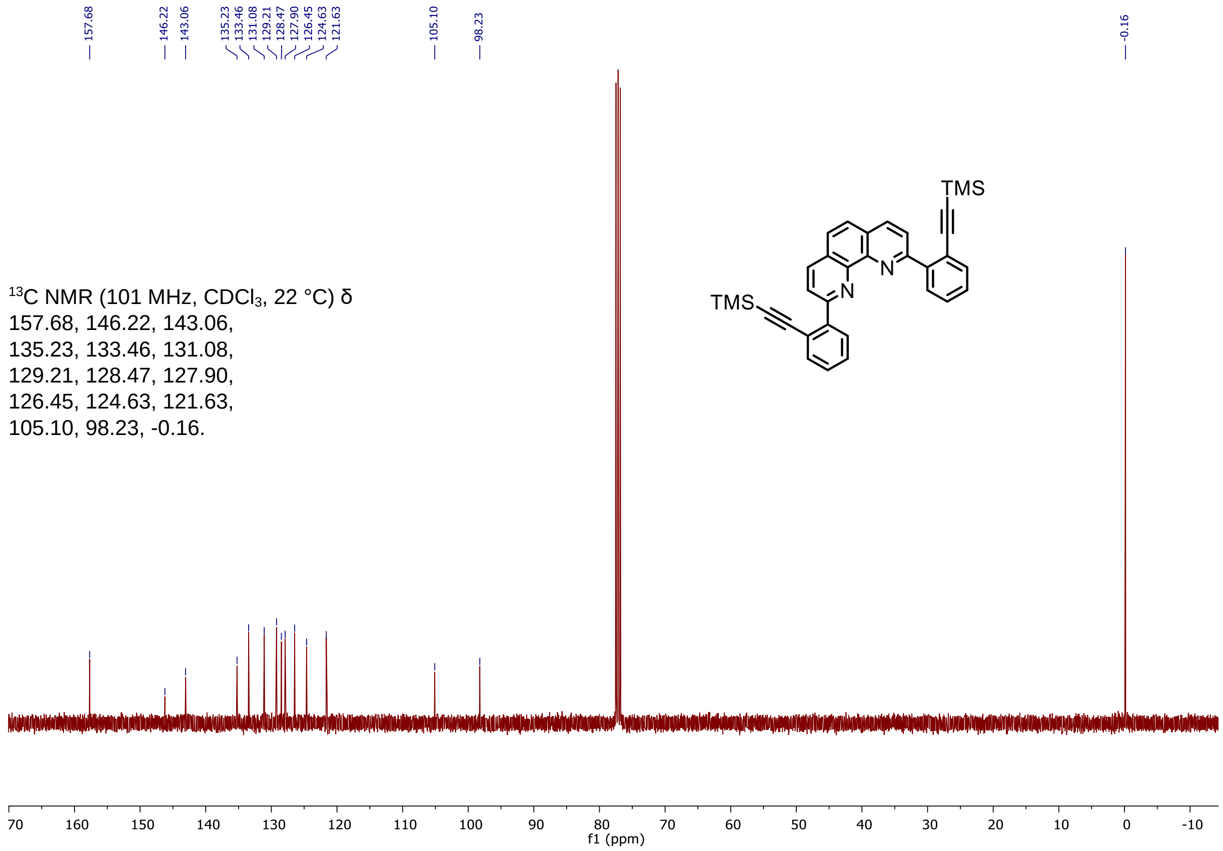

^{13}C NMR (101 MHz, $CDCl_3$, 22 °C) δ
157.68, 146.22, 143.06,
135.23, 133.46, 131.08,
129.21, 128.47, 127.90,
126.45, 124.63, 121.63,
105.10, 98.23, -0.16.

High Resolution Mass Spectrometry Report

Sample Name	**Thomas Brandl / BRT533**	Instrument	maXis 4G
Comment	10 ug/mL in MeCN, analyzed in MeCN	Method	22 Direct_pos_mid.m

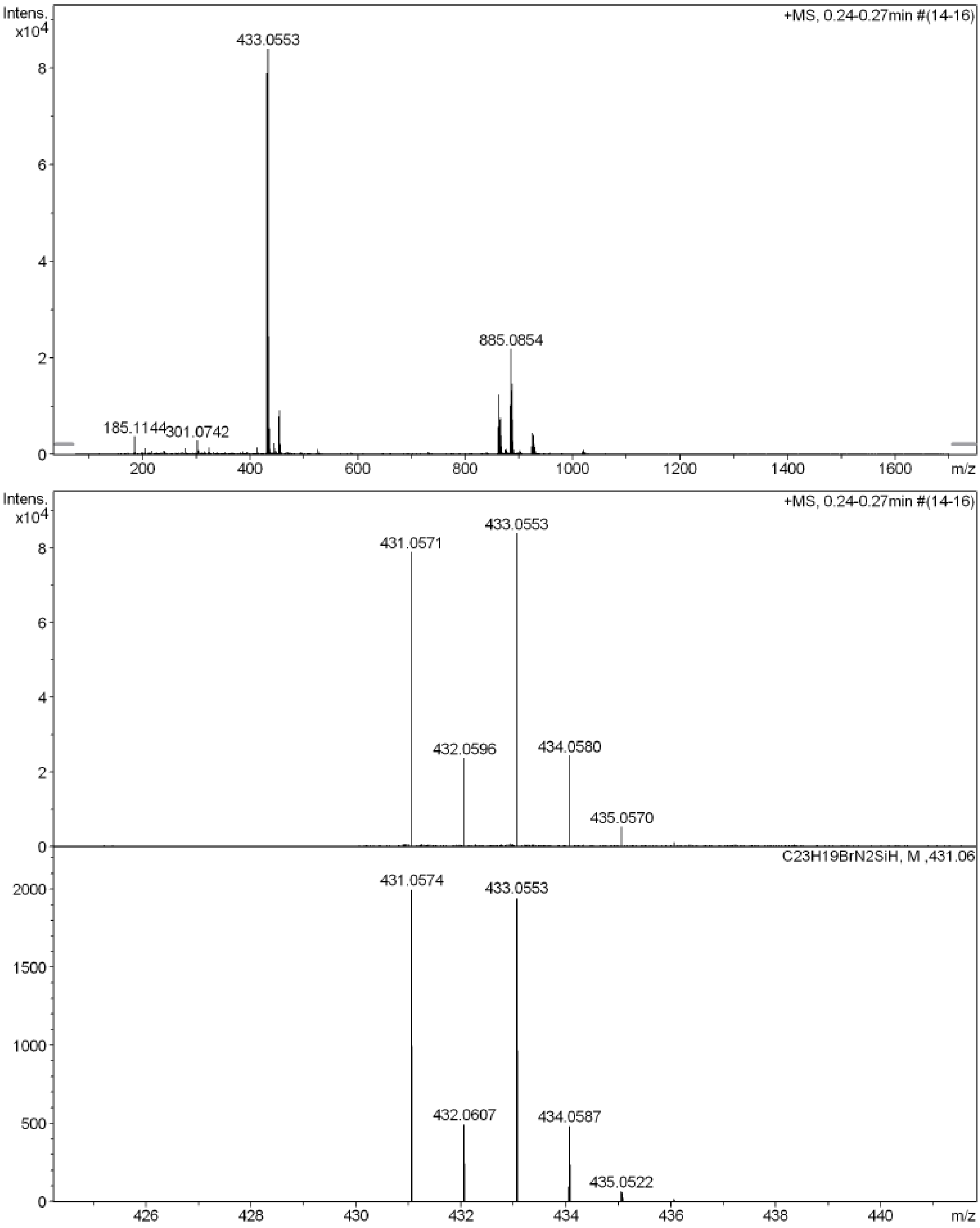

High Resolution Mass Spectrometry Report

Measured m/z vs. theoretical m/z

Meas. m/z	#	Formula	Score	m/z	err [mDa]	err [ppm]	mSigma	rdb	e⁻ Conf	z
431.0571	1	C 23 H 20 Br N 2 Si	100.00	431.0574	0.2	0.6	7.6	15.5	even	1+
453.0395	1	C 23 H 19 Br N 2 Na Si	100.00	453.0393	-0.2	-0.4	51.5	15.5	even	

Mass list

#	m/z	I %	I
1	185.1144	4.4	3727
2	186.1179	0.5	417
3	197.0787	0.5	395
4	201.1025	0.5	401
5	205.0592	1.5	1234
6	217.1043	0.8	634
7	239.0869	0.8	684
8	241.0825	0.7	621
9	273.1685	0.5	448
10	279.0923	1.5	1279
11	280.0959	0.5	430
12	301.0742	3.4	2863
13	301.1396	0.9	727
14	302.0781	0.9	792
15	304.2615	0.9	772
16	315.1913	0.7	578
17	322.1654	0.5	449
18	323.1606	1.7	1434
19	331.2068	0.6	493
20	387.1072	0.7	548
21	393.2980	0.5	451
22	413.2656	1.7	1422
23	430.8919	0.6	491
24	430.9182	0.6	525
25	430.9428	0.6	480
26	430.9695	0.8	633
27	431.0017	0.5	444
28	431.0571	93.9	78815
29	431.2372	0.6	495
30	431.2574	0.6	462
31	431.2911	0.5	400
32	431.9708	0.5	418
33	432.0596	28.3	23763
34	432.2830	0.5	446
35	432.7568	0.6	480
36	432.9057	0.6	486
37	432.9322	0.8	697
38	432.9578	0.6	519
39	432.9897	0.8	674
40	433.0553	100.0	83898
41	433.2897	0.5	432
42	433.3500	0.5	426
43	433.3737	0.5	398
44	434.0580	29.0	24339
45	435.0570	6.4	5348
46	436.0587	1.3	1073
47	444.1894	2.8	2358
48	445.1917	1.5	1277
49	447.3438	0.8	664
50	453.0395	9.3	7761
51	453.1746	0.6	464
52	454.0412	3.0	2514
53	455.0375	10.9	9184
54	456.0389	2.5	2139
55	457.0385	0.6	478
56	469.0118	0.6	476
57	494.9770	0.5	409
58	525.2173	1.3	1084
59	526.2188	0.7	587
60	731.1234	0.6	475
61	861.1044	6.8	5685

High Resolution Mass Spectrometry Report

#	m/z	I %	I
62	862.1095	4.3	3584
63	863.1033	14.8	12428
64	864.1059	8.5	7132
65	865.1037	9.0	7514
66	866.1052	4.7	3916
67	867.1018	2.0	1686
68	868.1071	0.6	469
69	874.2357	1.2	990
70	875.2382	1.0	815
71	876.2367	1.3	1119
72	877.2371	0.8	673
73	878.2382	0.5	436
74	883.0863	12.1	10145
75	884.0903	7.4	6209
76	884.9927	0.5	422
77	885.0854	26.0	21811
78	885.8562	0.6	462
79	886.0873	15.7	13136
80	887.0849	17.4	14595
81	888.0865	8.5	7172
82	889.0847	3.3	2764
83	890.0882	1.1	927
84	899.0593	0.5	385
85	901.0569	0.9	733
86	902.0610	0.6	463
87	903.0569	0.6	464
88	923.0279	2.0	1659
89	924.0287	1.3	1118
90	925.0266	5.3	4462
91	926.0261	3.2	2680
92	927.0239	4.7	3961
93	928.0244	2.6	2149
94	929.0226	1.9	1563
95	930.0275	0.8	654
96	931.0224	0.5	385
97	1017.1866	0.7	603
98	1019.1882	1.2	983
99	1020.1871	0.7	556
100	1021.1833	0.5	417

Acquisition Parameter

Source Type	ESI	Ion Polarity	Positive	Set Nebulizer	0.4 Bar
Focus	Not active	Set Capillary	3600 V	Set Dry Heater	180 °C
Scan Begin	75 m/z	Set End Plate Offset	-500 V	Set Dry Gas	4.0 l/min
Scan End	1700 m/z	Set Collision Cell RF	350.0 Vpp	Set Ion Energy (MS only)	4.0 eV

^1H-, ^{13}C-NMR (CD_2Cl_2, 400/101 MHz, 22 °C) and HR-ESI-MS spectra of compound (4)

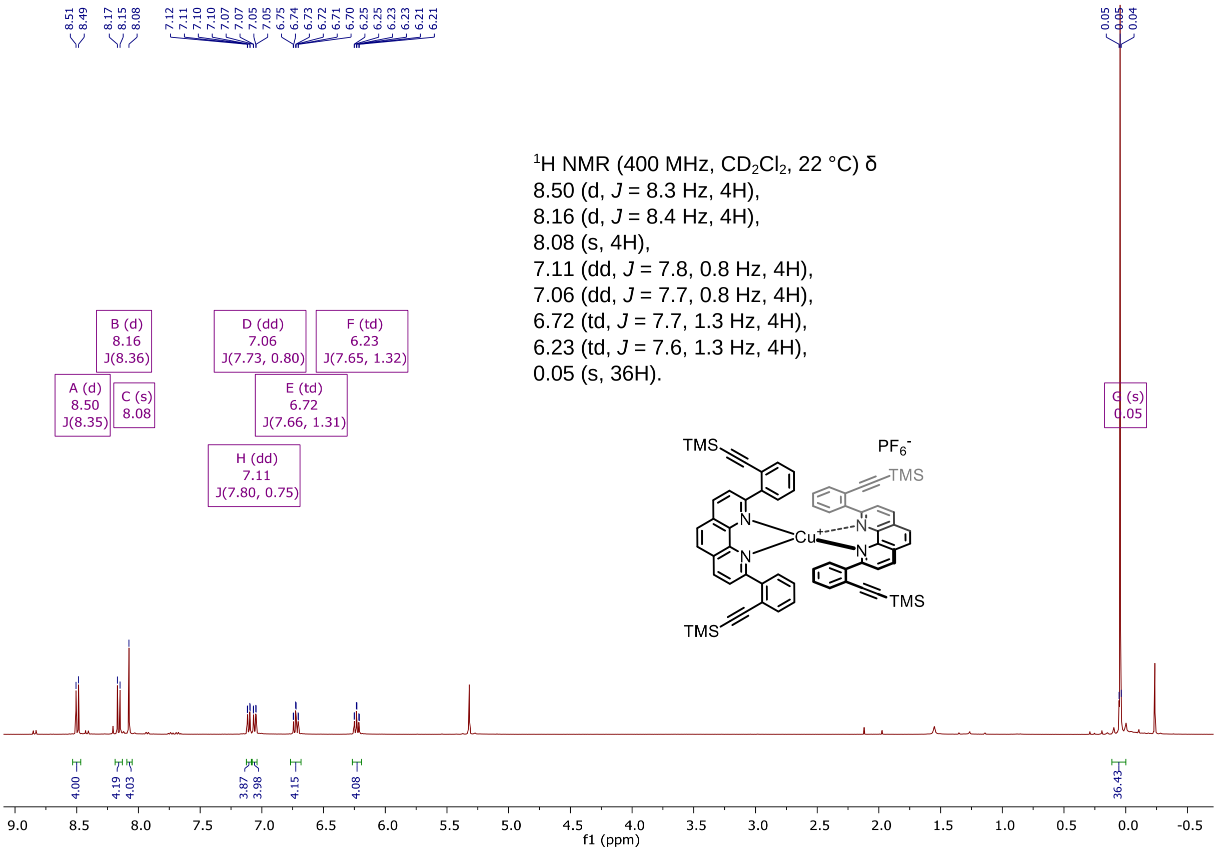

^1H NMR (400 MHz, CD_2Cl_2, 22 °C) δ
8.50 (d, *J* = 8.3 Hz, 4H),
8.16 (d, *J* = 8.4 Hz, 4H),
8.08 (s, 4H),
7.11 (dd, *J* = 7.8, 0.8 Hz, 4H),
7.06 (dd, *J* = 7.7, 0.8 Hz, 4H),
6.72 (td, *J* = 7.7, 1.3 Hz, 4H),
6.23 (td, *J* = 7.6, 1.3 Hz, 4H),
0.05 (s, 36H).

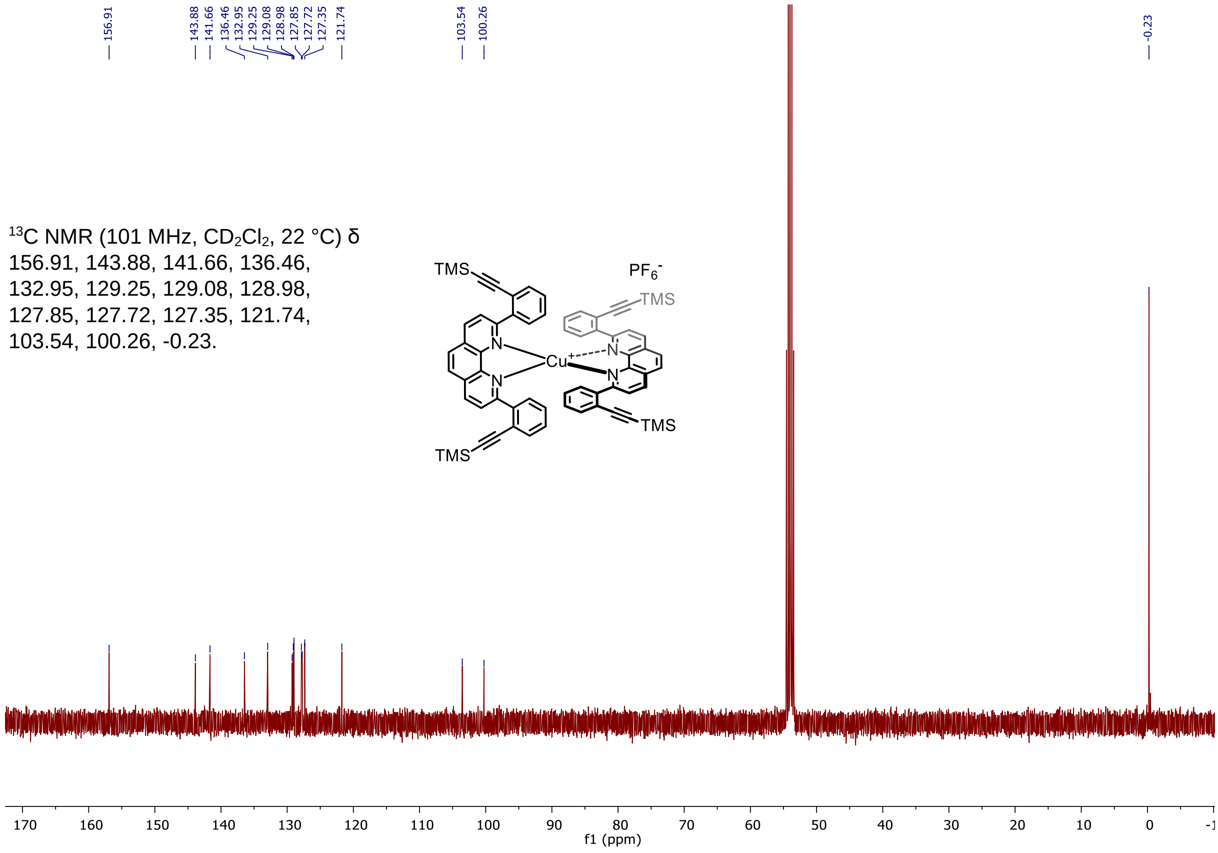

^{13}C NMR (101 MHz, CD_2Cl_2, 22 °C) δ
156.91, 143.88, 141.66, 136.46,
132.95, 129.25, 129.08, 128.98,
127.85, 127.72, 127.35, 121.74,
103.54, 100.26, -0.23.

High Resolution Mass Spectrometry Report

Sample Name	**Thomas Brandl / LL1357**	Instrument	maXis 4G
Comment	10 ug/mL in MeCN, analyzed in MeCN	Method	23 Direct_pos_higher.m

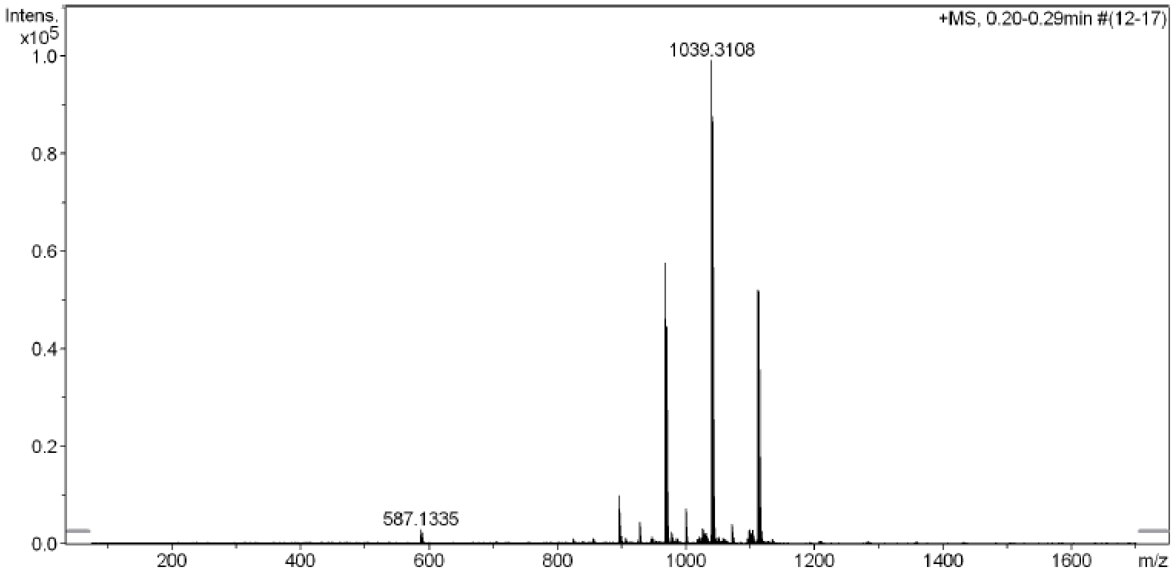

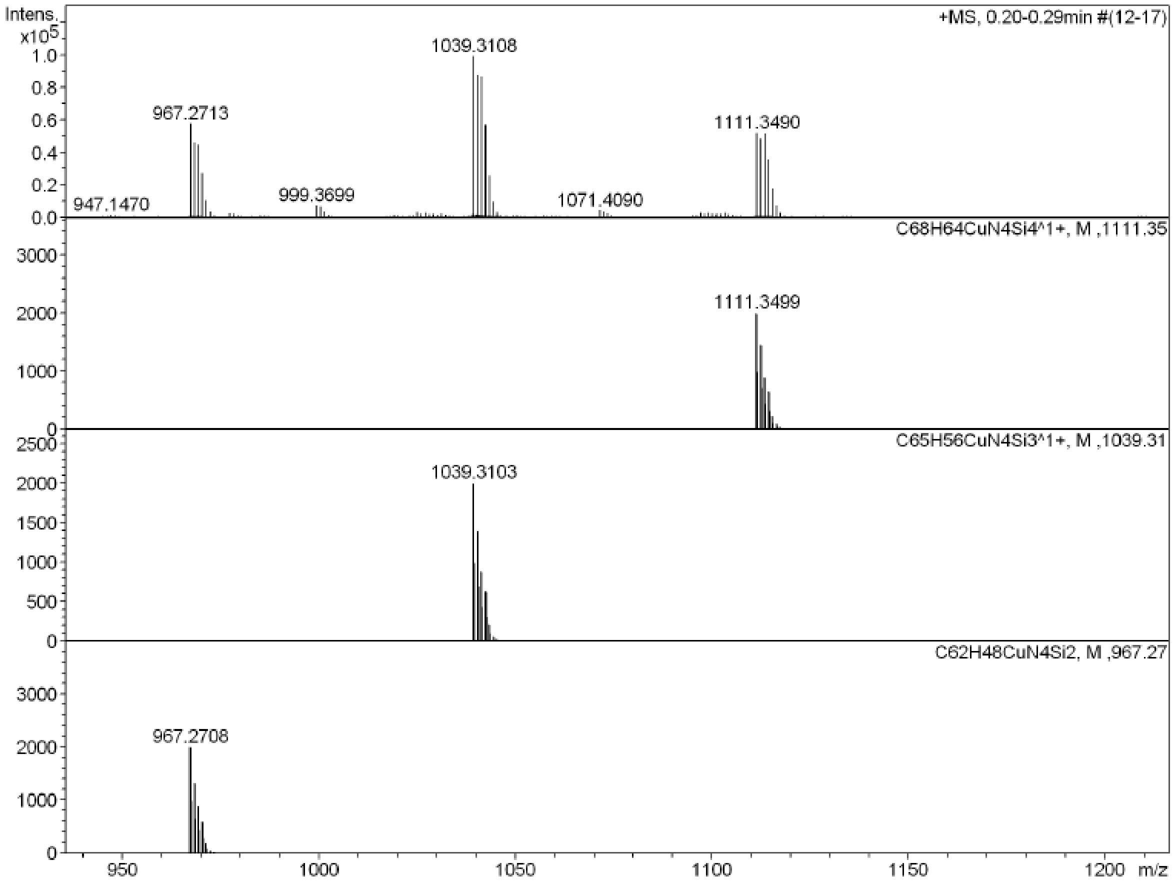

High Resolution Mass Spectrometry Report

Measured m/z vs. theoretical m/z

Meas. m/z	#	Formula	Score	m/z	err [mDa]	err [ppm]	mSigma	rdb	e⁻ Conf	z
967.2713	1	C 62 H 48 Cu N 4 Si 2	100.00	967.2708	-0.5	-0.5	18.6	42.5	even	1+
1039.3108	1	C 65 H 56 Cu N 4 Si 3	100.00	1039.3103	-0.4	-0.4	20.2	42.5	even	
1111.3490	1	C 68 H 64 Cu N 4 Si 4	100.00	1111.3499	0.8	0.8	13.4	42.5	even	

Mass list

#	m/z	I %	I
1	587.1335	3.0	2930
2	588.1360	1.7	1691
3	589.1306	2.2	2220
4	590.1348	1.2	1164
5	823.1909	0.9	917
6	855.2908	1.0	968
7	895.2316	10.0	9860
8	896.2352	7.1	7059
9	897.2318	6.4	6340
10	898.2333	3.7	3704
11	899.2367	1.5	1469
12	905.3495	1.0	1036
13	906.3529	1.0	966
14	927.3315	4.5	4419
15	928.3331	3.5	3507
16	929.3346	1.7	1672
17	947.1470	1.4	1371
18	948.1497	0.9	886
19	967.2713	58.1	57519
20	968.2740	46.5	46022
21	969.1371	1.1	1046
22	969.2718	45.0	44543
23	970.2724	27.5	27270
24	971.2739	10.6	10486
25	972.2731	3.6	3582
26	973.2713	1.0	1005
27	977.3862	2.3	2311
28	978.3919	2.0	2008
29	979.3907	1.2	1165
30	985.1215	1.0	1027
31	999.3699	7.2	7169
32	1000.3736	6.6	6511
33	1001.3743	3.3	3305
34	1002.3739	1.4	1424
35	1019.1877	1.4	1340
36	1020.1911	1.0	961
37	1023.0602	1.2	1199
38	1024.0620	0.9	893
39	1025.0589	3.1	3112
40	1026.0610	2.0	1944
41	1027.0584	2.9	2921
42	1028.0586	1.7	1689
43	1029.0602	1.3	1271
44	1029.1915	2.0	1948
45	1030.1961	1.3	1298
46	1031.1925	2.1	2103
47	1032.1946	1.5	1510
48	1033.1922	1.1	1094
49	1039.0177	1.0	950
50	1039.1036	1.7	1700
51	1039.3108	100.0	99036
52	1039.8161	1.1	1089
53	1039.8837	1.5	1524
54	1040.0740	1.3	1247
55	1040.1331	1.7	1704
56	1040.2009	1.1	1132
57	1040.3132	88.4	87572
58	1040.4046	0.9	863
59	1040.6529	1.5	1462
60	1040.7958	0.9	879

#	m/z	I %	I
61	1040.9051	1.0	1019
62	1041.0291	1.1	1044
63	1041.0861	1.0	1006
64	1041.1575	1.0	1015
65	1041.3113	87.2	86394
66	1041.6098	1.1	1079
67	1042.1291	1.0	1036
68	1042.3122	57.1	56593
69	1043.1471	1.0	961
70	1043.3123	25.7	25428
71	1044.3127	9.7	9572
72	1045.3134	3.3	3279
73	1046.3158	0.9	882
74	1049.4257	1.1	1138
75	1050.4299	1.3	1285
76	1057.1586	1.0	1002
77	1071.4090	4.1	4020
78	1072.4119	3.5	3440
79	1073.4130	2.3	2311
80	1074.4132	1.1	1116
81	1095.0983	1.0	956
82	1096.0990	1.0	954
83	1097.0972	2.6	2603
84	1098.1014	2.0	1965
85	1099.0961	2.9	2851
86	1100.0978	2.1	2049
87	1101.0987	1.4	1418
88	1101.2295	2.0	1984
89	1102.2327	1.9	1907
90	1103.2306	2.8	2742
91	1104.2321	1.8	1755
92	1105.2304	1.3	1330
93	1111.3490	52.5	51998
94	1112.3513	48.8	48359
95	1113.3496	52.1	51615
96	1114.3508	36.0	35669
97	1115.3503	17.9	17771
98	1116.3507	7.5	7416
99	1117.3504	2.6	2598
100	1118.3505	0.9	936

Acquisition Parameter

Source Type	ESI	Ion Polarity	Positive	Set Nebulizer	0.4 Bar
Focus	Not active	Set Capillary	3600 V	Set Dry Heater	180 °C
Scan Begin	75 m/z	Set End Plate Offset	-500 V	Set Dry Gas	4.0 l/min
Scan End	1700 m/z	Set Collision Cell RF	500.0 Vpp	Set Ion Energy (MS only)	4.0 eV

^{1}H-, ^{13}C-NMR (CD_2Cl_2, 400/101 MHz, 22 °C) and HR-ESI-MS spectra of compound (11)

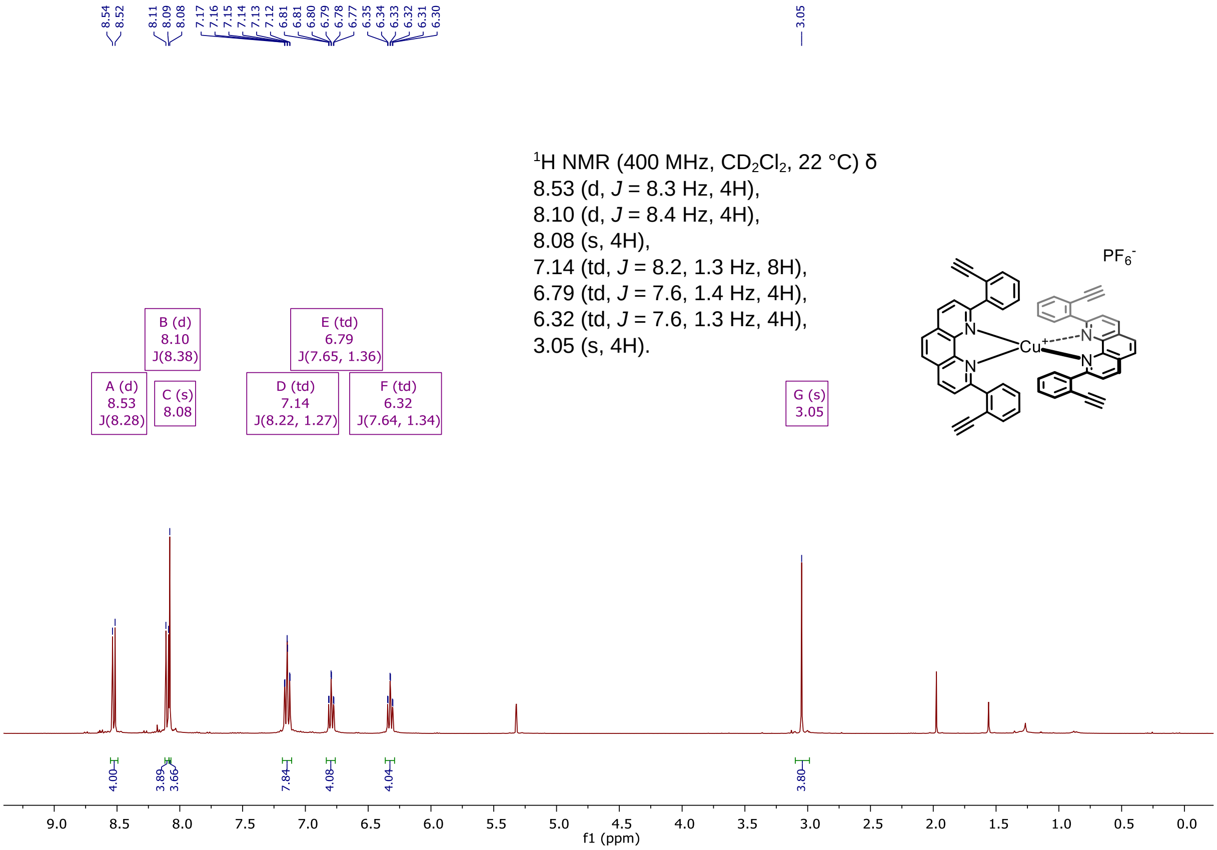

^{1}H NMR (400 MHz, CD_2Cl_2, 22 °C) δ
8.53 (d, J = 8.3 Hz, 4H),
8.10 (d, J = 8.4 Hz, 4H),
8.08 (s, 4H),
7.14 (td, J = 8.2, 1.3 Hz, 8H),
6.79 (td, J = 7.6, 1.4 Hz, 4H),
6.32 (td, J = 7.6, 1.3 Hz, 4H),
3.05 (s, 4H).

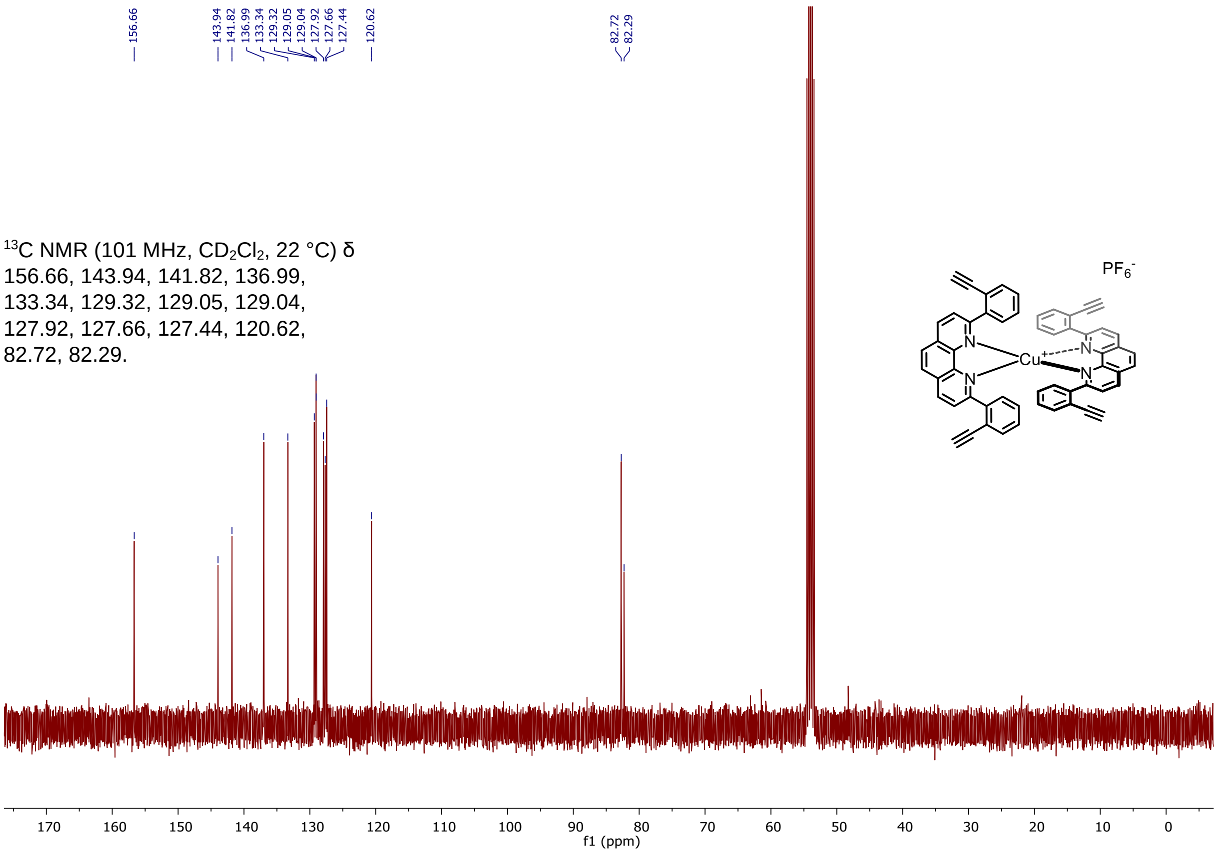

^{13}C NMR (101 MHz, CD_2Cl_2, 22 °C) δ
156.66, 143.94, 141.82, 136.99,
133.34, 129.32, 129.05, 129.04,
127.92, 127.66, 127.44, 120.62,
82.72, 82.29.

High Resolution Mass Spectrometry Report

Sample Name	**Thomas Brandl / LL1358**	Instrument	maXis 4G
Comment	10 ug/mL in MeCN, analyzed in MeCN	Method	23 Direct_pos_higher.m

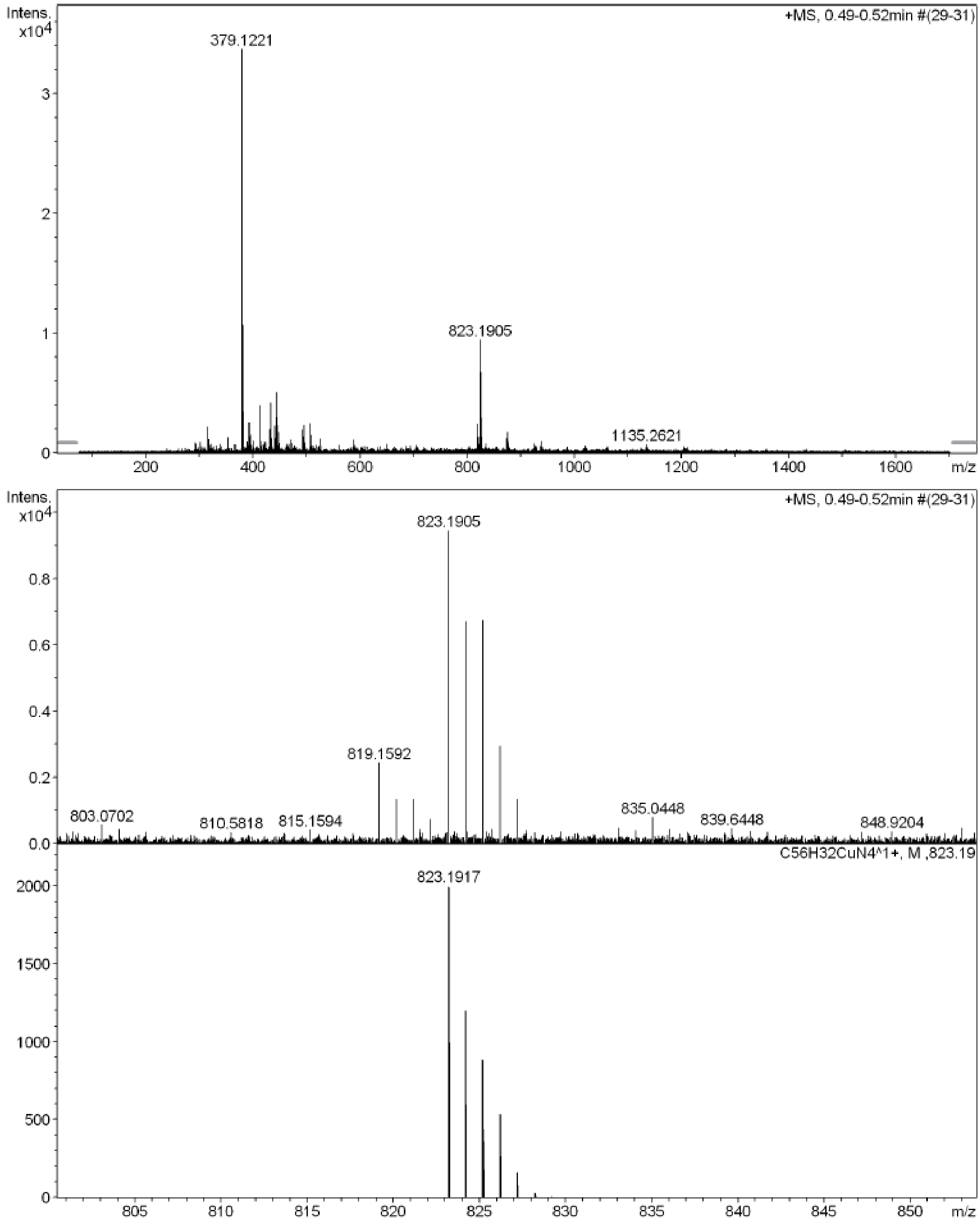

High Resolution Mass Spectrometry Report

Measured m/z vs. theoretical m/z

Meas. m/z	#	Formula	Score	m/z	err [mDa]	err [ppm]	mSigma	rdb	e⁻ Conf	z
823.1905	1	C 56 H 32 Cu N 4	100.00	823.1917	1.3	1.5	51.9	42.5	even	1+

Mass list

#	m/z	I %	I
1	292.0836	2.5	851
2	292.5854	1.9	632
3	293.2429	2.2	742
4	301.1398	2.6	891
5	315.1603	2.6	892
6	315.1913	6.6	2210
7	316.1666	2.0	669
8	317.1721	3.4	1133
9	319.1857	2.0	665
10	321.2391	2.2	725
11	321.2744	1.8	615
12	331.1061	1.8	613
13	339.1768	2.3	760
14	353.2648	3.8	1277
15	365.2670	2.1	716
16	367.2079	2.1	713
17	379.1221	100.0	33678
18	380.1256	29.4	9917
19	381.1375	31.8	10711
20	381.2971	3.8	1277
21	382.1410	10.2	3442
22	389.0283	2.8	943
23	391.0266	1.9	651
24	391.2845	2.6	872
25	392.0096	7.5	2532
26	393.0069	4.1	1382
27	393.2971	3.8	1284
28	394.0078	7.5	2539
29	395.0047	4.6	1541
30	397.1334	2.0	669
31	400.0929	3.1	1037
32	413.1275	2.0	667
33	413.2654	11.8	3960
34	414.2695	2.9	975
35	419.0894	2.0	686
36	420.9384	1.8	607
37	421.3285	2.7	920
38	422.9380	2.7	904
39	431.0568	5.8	1942
40	432.0586	1.9	638
41	433.0544	5.9	1974
42	433.1026	12.5	4198
43	434.1071	3.6	1199
44	441.0443	6.8	2283
45	441.2967	3.6	1219
46	442.0477	2.5	829
47	443.0582	15.0	5041
48	444.0632	5.4	1833
49	444.1891	8.9	2981
50	445.0579	6.8	2301
51	445.1914	3.2	1090
52	446.0592	1.8	613
53	447.3438	5.2	1744
54	449.3595	2.5	841
55	463.3757	2.2	740
56	467.3113	2.2	753
57	469.3287	3.2	1093
58	473.3170	2.4	822
59	473.3410	1.9	642
60	477.0643	1.9	633
61	492.1177	5.7	1915
62	492.9778	4.0	1361

#	m/z	I %	I
63	493.1222	1.9	645
64	493.9806	1.9	641
65	494.9759	6.9	2317
66	495.9797	2.5	828
67	496.9757	2.5	852
68	506.1113	7.4	2482
69	507.1136	3.1	1033
70	508.1093	4.4	1484
71	509.1138	1.9	627
72	511.6423	1.8	611
73	517.3698	2.0	672
74	525.2172	3.5	1179
75	561.3955	1.9	656
76	587.1381	3.3	1101
77	589.1368	2.0	677
78	649.4486	2.2	745
79	685.4365	1.8	601
80	693.4647	1.9	633
81	705.5813	2.0	672
82	819.1592	7.2	2433
83	820.1625	4.0	1342
84	821.1574	4.0	1346
85	822.1644	2.2	738
86	823.1905	28.0	9436
87	824.1935	19.9	6694
88	825.1910	20.0	6737
89	826.1918	8.7	2941
90	827.1940	4.0	1341
91	835.0448	2.3	790
92	873.1089	3.6	1215
93	874.1108	2.0	672
94	875.1096	5.2	1766
95	876.1131	2.9	964
96	877.1067	2.2	758
97	925.0251	2.3	773
98	938.1560	3.0	994
99	1019.1860	1.9	625
100	1135.2621	2.1	700

Acquisition Parameter

Source Type	ESI	Ion Polarity	Positive	Set Nebulizer	0.4 Bar
Focus	Not active	Set Capillary	3600 V	Set Dry Heater	180 °C
Scan Begin	75 m/z	Set End Plate Offset	-500 V	Set Dry Gas	4.0 l/min
Scan End	1700 m/z	Set Collision Cell RF	500.0 Vpp	Set Ion Energy (MS only)	4.0 eV

^{1}H-, ^{13}C-, HMBC-NMR (CD_2Cl_2, 400/126 MHz, 22/25 °C) and HR-ESI-MS spectra of compound (2)

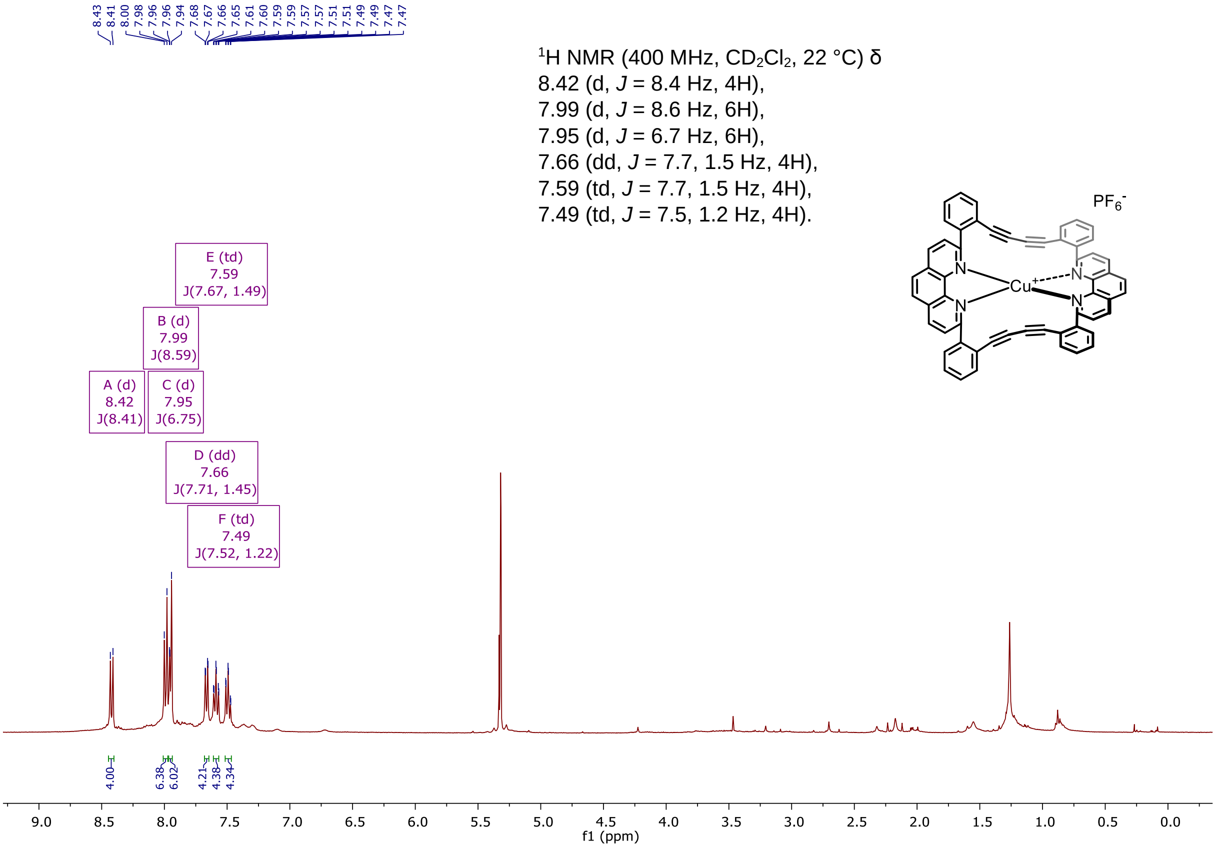

^{1}H NMR (400 MHz, CD_2Cl_2, 22 °C) δ
8.42 (d, *J* = 8.4 Hz, 4H),
7.99 (d, *J* = 8.6 Hz, 6H),
7.95 (d, *J* = 6.7 Hz, 6H),
7.66 (dd, *J* = 7.7, 1.5 Hz, 4H),
7.59 (td, *J* = 7.7, 1.5 Hz, 4H),
7.49 (td, *J* = 7.5, 1.2 Hz, 4H).

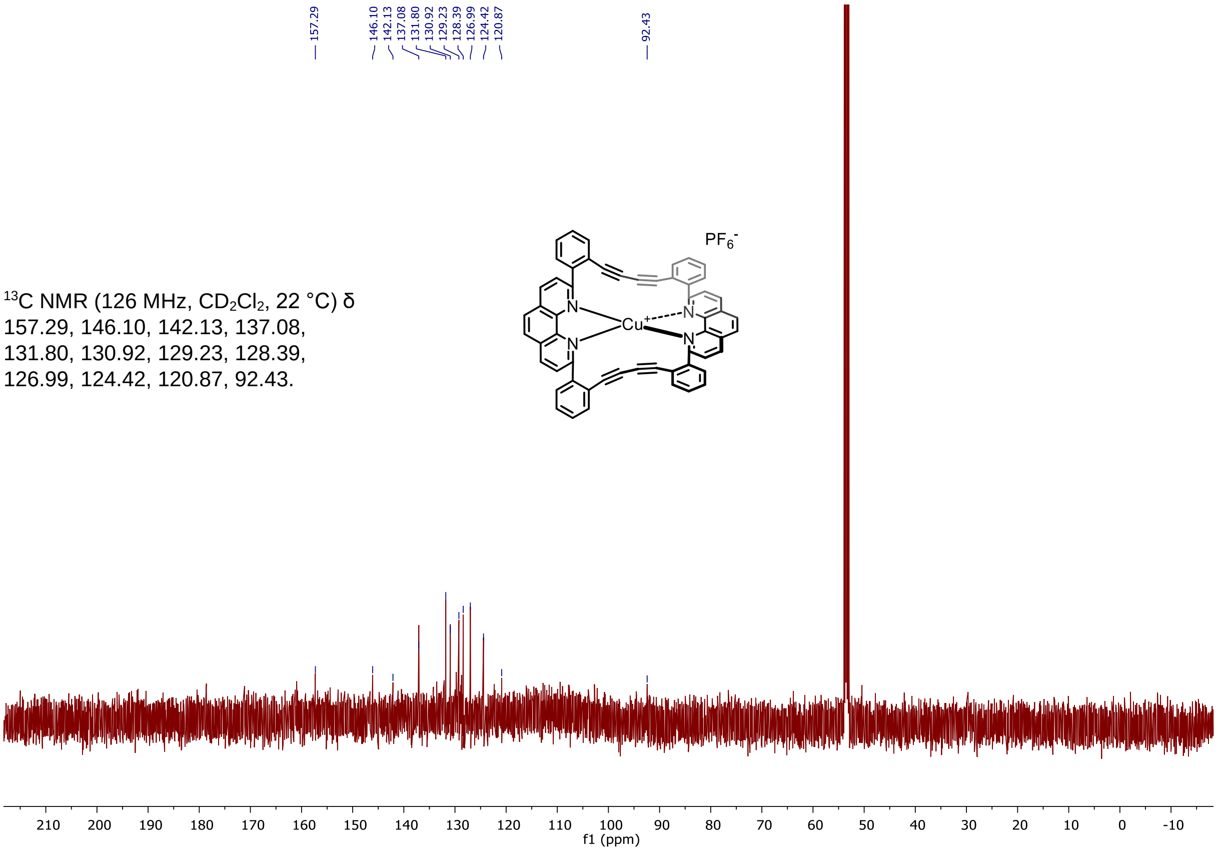

^{13}C NMR (126 MHz, CD_2Cl_2, 22 °C) δ
157.29, 146.10, 142.13, 137.08,
131.80, 130.92, 129.23, 128.39,
126.99, 124.42, 120.87, 92.43.

HMBC

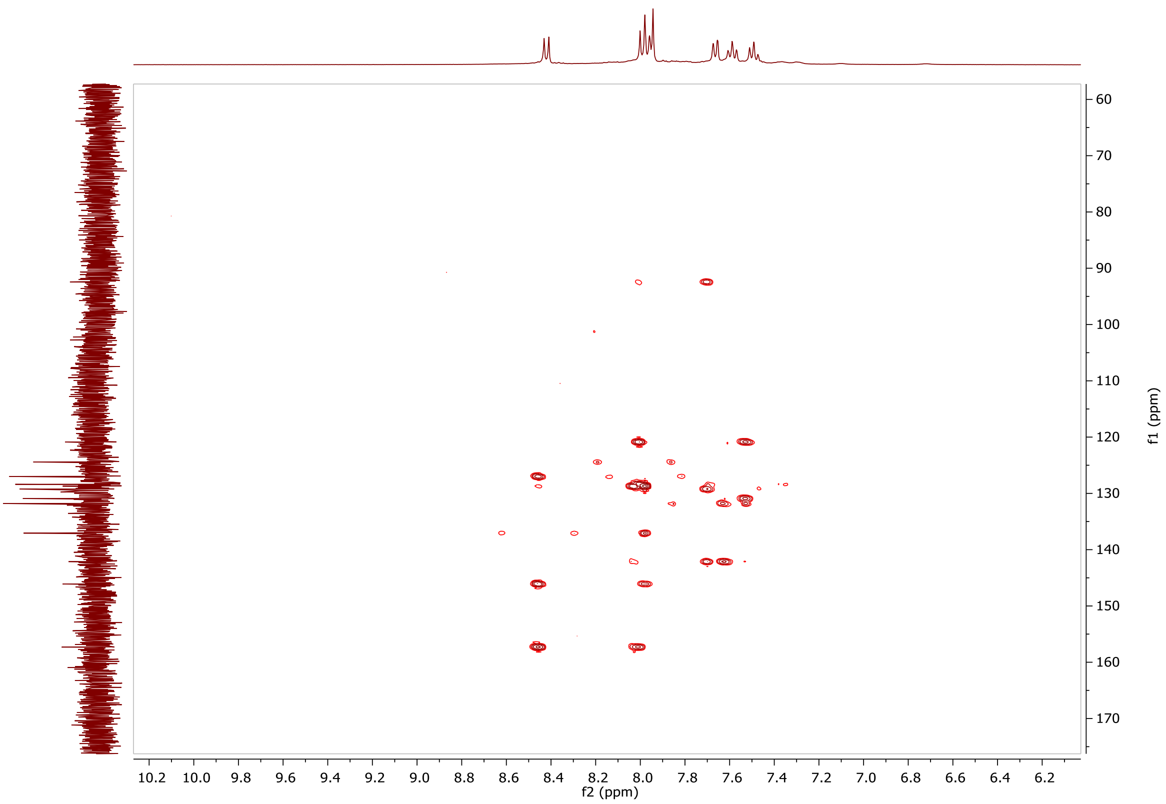

f1 (ppm)
60
70
80
90
100
110
120
130
140
150
160
170
10.2 10.0 9.8 9.6 9.4 9.2 9.0 8.8 8.6 8.4 8.2 8.0 7.8 7.6 7.4 7.2 7.0 6.8 6.6 6.4 6.2
f2 (ppm)

High Resolution Mass Spectrometry Report

Sample Name	**Thomas Brandl / LL1359**	Instrument	maXis 4G
Comment	10 ug/mL in MeCN, analyzed in MeCN	Method	23 Direct_pos_higher.m

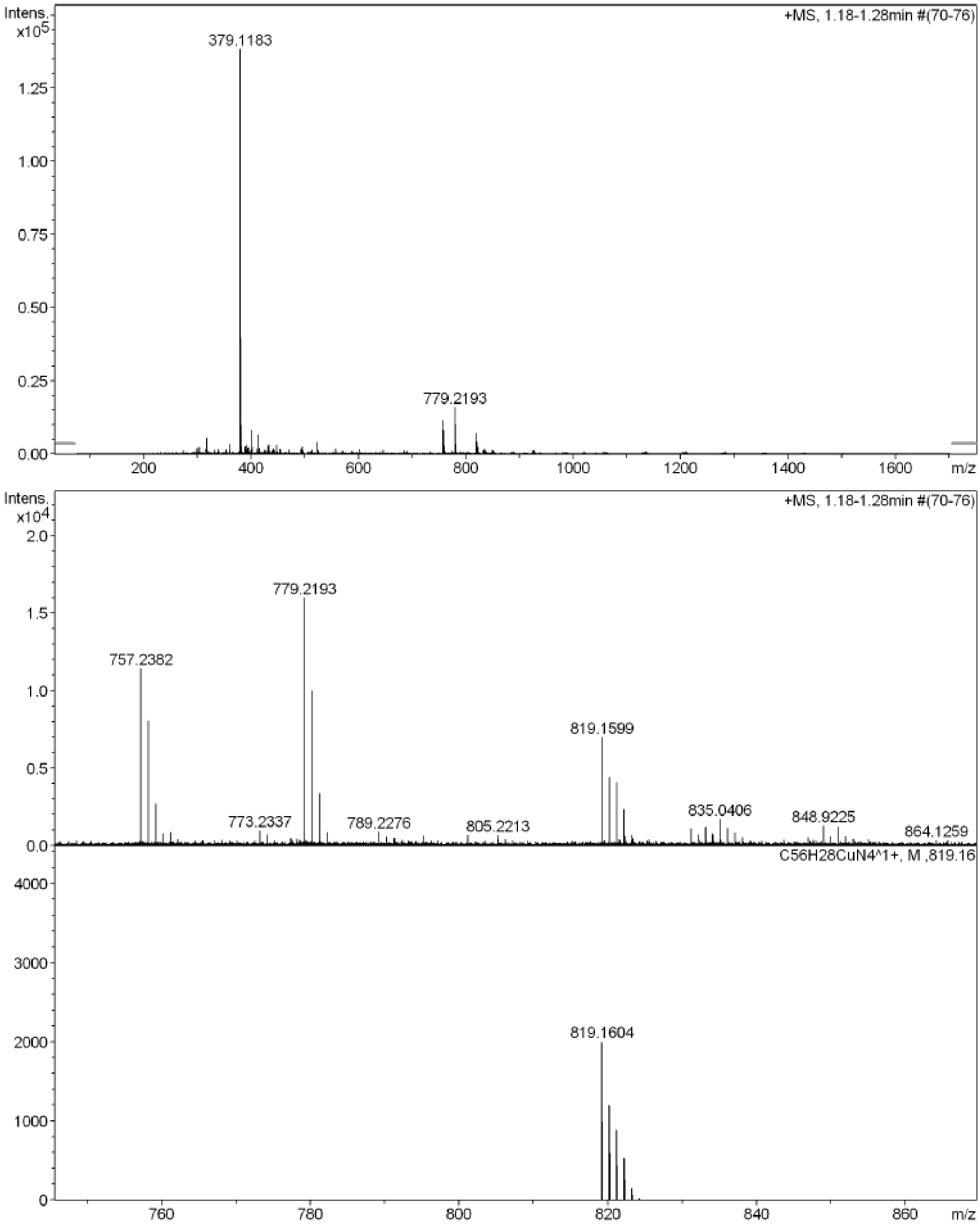

High Resolution Mass Spectrometry Report

Measured m/z vs. theoretical m/z

Meas. m/z	#	Formula	Score	m/z	err [mDa]	err [ppm]	mSigma	rdb	e⁻ Conf	z
819.1599	1	C 56 H 28 Cu N 4	100.00	819.1604	0.5	0.6	25.1	44.5	even	1+

Mass list

#	m/z	I %	I
1	273.1544	0.8	1139
2	299.1512	1.2	1705
3	301.0650	0.6	805
4	301.1311	1.6	2257
5	304.2509	1.7	2356
6	315.1839	1.2	1685
7	317.1631	3.9	5428
8	318.1668	0.8	1136
9	331.1795	0.6	778
10	331.2008	1.1	1525
11	339.1470	1.1	1548
12	339.1705	0.6	801
13	353.2599	1.0	1440
14	360.3173	2.3	3208
15	361.3211	0.7	1029
16	379.0349	0.6	845
17	379.1183	100.0	138278
18	380.1213	28.6	39614
19	381.1293	7.1	9870
20	381.2924	0.8	1134
21	382.1356	1.9	2641
22	389.0239	1.9	2634
23	391.0220	2.1	2948
24	392.0065	0.8	1127
25	393.0017	1.6	2154
26	393.2937	0.8	1167
27	394.0041	1.2	1628
28	394.9997	1.6	2254
29	395.1139	0.9	1201
30	399.1089	1.1	1489
31	401.1009	5.8	8064
32	402.1042	1.8	2514
33	411.1093	1.2	1692
34	412.1141	0.6	852
35	413.1244	1.8	2532
36	413.2631	4.6	6351
37	414.1281	0.6	783
38	414.2657	1.3	1807
39	415.1035	1.4	1888
40	424.1051	1.1	1460
41	425.2844	0.6	830
42	427.0882	0.8	1112
43	431.0540	2.0	2792
44	432.0578	0.7	932
45	433.0528	2.2	3000
46	433.0994	1.4	1964
47	434.0554	0.8	1104
48	438.9238	0.6	897
49	441.0412	0.9	1265
50	441.2950	1.2	1720
51	444.1864	0.9	1293
52	447.3421	2.0	2832
53	448.3448	0.6	793
54	453.0375	1.2	1652
55	455.0357	1.2	1663
56	469.3171	1.0	1388
57	492.1171	0.9	1209
58	492.9770	1.3	1840
59	494.9760	1.9	2626
60	495.9786	0.6	804
61	496.9725	0.7	924
62	506.1103	0.6	875

#	m/z	I %	I
63	511.6415	0.6	890
64	513.3391	1.0	1383
65	523.3231	2.9	4002
66	524.3264	0.9	1282
67	525.2176	0.8	1037
68	557.3652	1.2	1713
69	569.0607	0.7	937
70	571.0590	0.7	911
71	587.1392	0.7	947
72	601.3916	1.1	1562
73	645.4173	1.0	1322
74	685.4338	0.8	1122
75	689.4446	0.7	970
76	757.2382	8.3	11416
77	758.2409	5.8	8035
78	759.2450	2.0	2708
79	761.2329	0.6	852
80	773.2337	0.7	977
81	779.2193	11.5	15958
82	780.2234	7.2	9991
83	781.2257	2.4	3359
84	782.2302	0.6	848
85	789.2276	0.6	877
86	819.1599	5.1	6994
87	820.1625	3.2	4389
88	821.1602	2.9	4059
89	822.1617	1.7	2332
90	831.1523	0.8	1107
91	833.0415	0.9	1188
92	833.1529	0.9	1205
93	834.0465	0.6	770
94	835.0406	1.2	1690
95	836.0419	0.8	1137
96	837.0419	0.6	821
97	848.9225	0.9	1272
98	850.9208	0.9	1202
99	925.0271	0.8	1135
100	927.0250	0.8	1104

Acquisition Parameter

Source Type	ESI	Ion Polarity	Positive	Set Nebulizer	0.4 Bar
Focus	Not active	Set Capillary	3600 V	Set Dry Heater	180 °C
Scan Begin	75 m/z	Set End Plate Offset	-500 V	Set Dry Gas	4.0 l/min
Scan End	1700 m/z	Set Collision Cell RF	500.0 Vpp	Set Ion Energy (MS only)	4.0 eV

^{1}H-, ^{13}C-NMR (CD_2Cl_2, 400/101 MHz, 22 °C) and HR-ESI-MS spectra of compound (12)

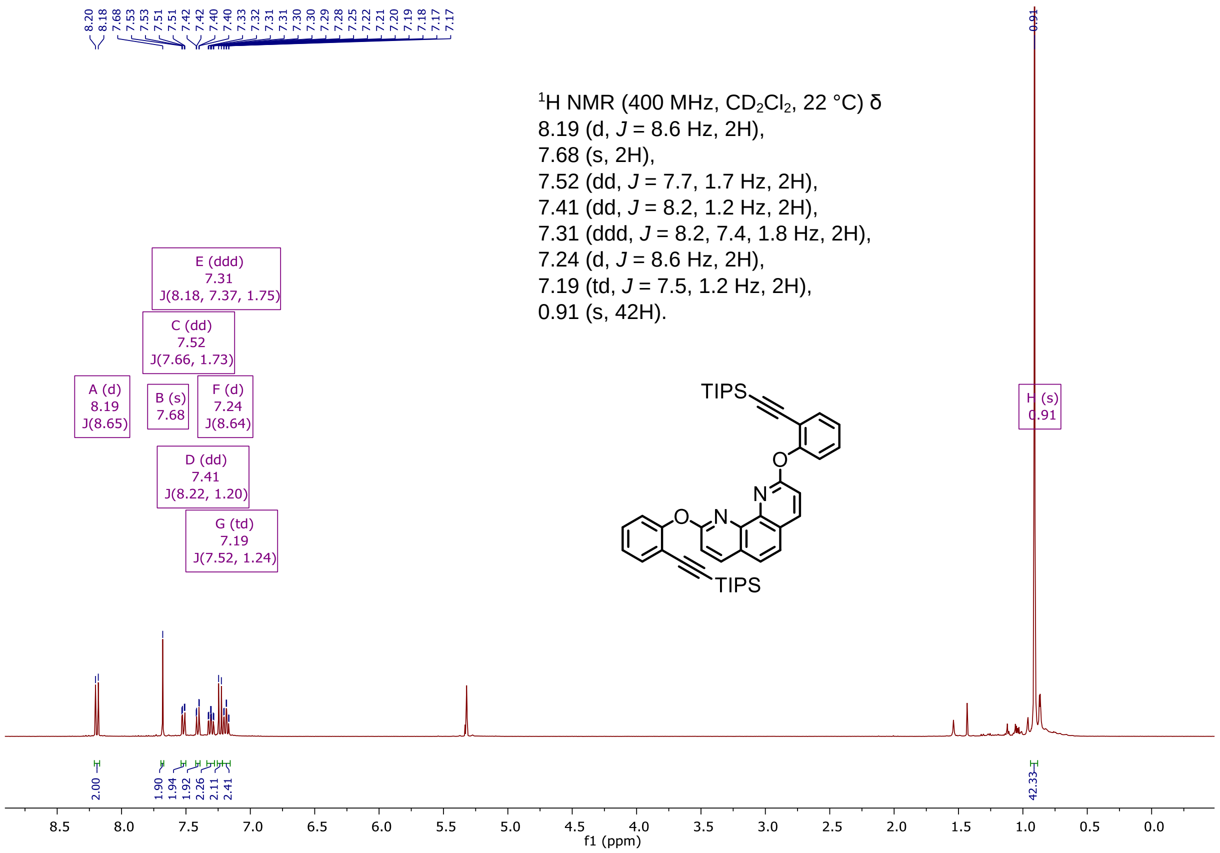

^{1}H NMR (400 MHz, CD_2Cl_2, 22 °C) δ
8.19 (d, J = 8.6 Hz, 2H),
7.68 (s, 2H),
7.52 (dd, J = 7.7, 1.7 Hz, 2H),
7.41 (dd, J = 8.2, 1.2 Hz, 2H),
7.31 (ddd, J = 8.2, 7.4, 1.8 Hz, 2H),
7.24 (d, J = 8.6 Hz, 2H),
7.19 (td, J = 7.5, 1.2 Hz, 2H),
0.91 (s, 42H).

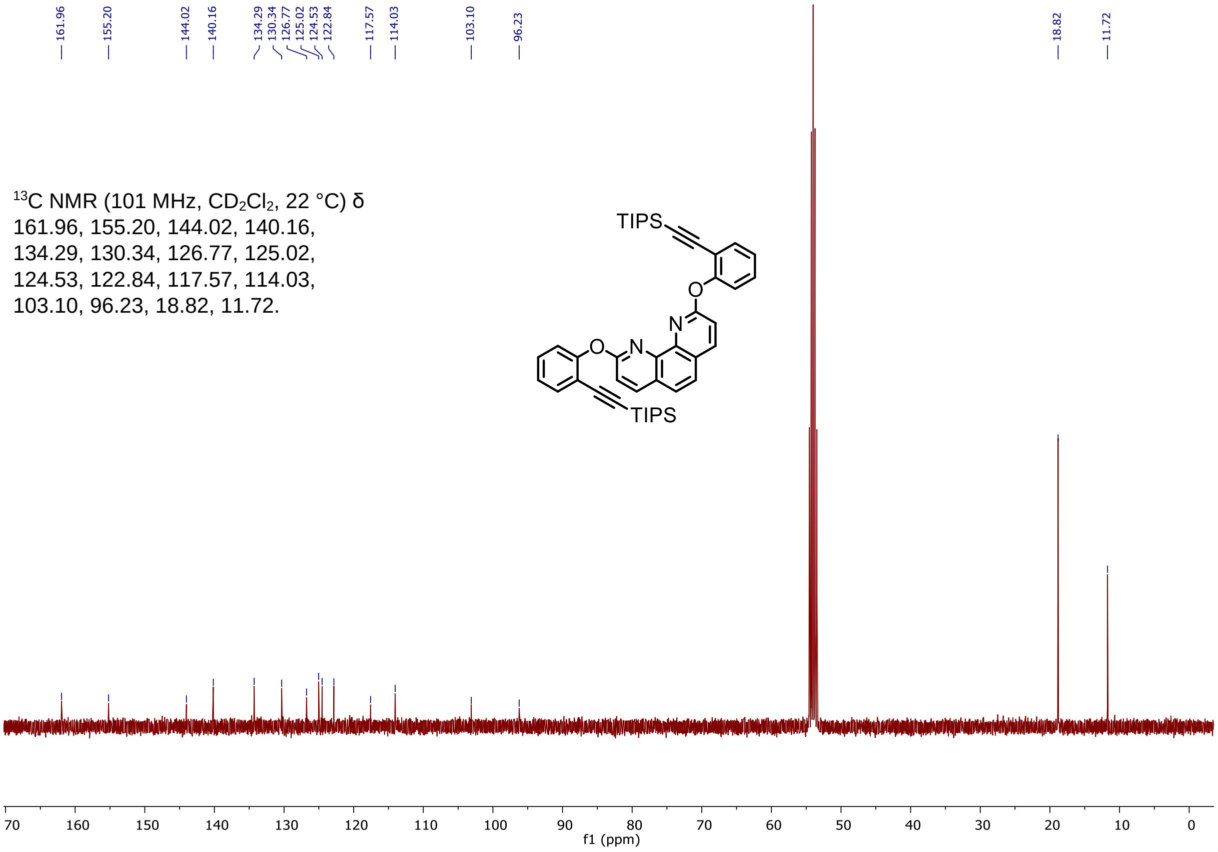

^{13}C NMR (101 MHz, CD_2Cl_2, 22 °C) δ
161.96, 155.20, 144.02, 140.16,
134.29, 130.34, 126.77, 125.02,
124.53, 122.84, 117.57, 114.03,
103.10, 96.23, 18.82, 11.72.

High Resolution Mass Spectrometry Report

Sample Name	**Thomas Brandl / LL1369**	Instrument	maXis 4G
Comment	10 ug/mL in MeCN, analyzed in MeCN	Method	22 Direct_pos_mid.m

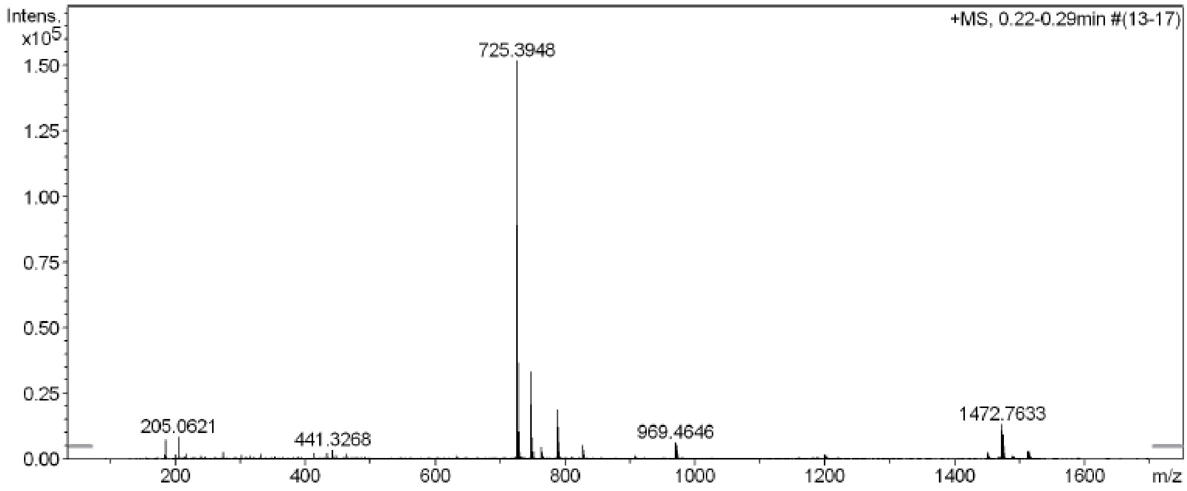

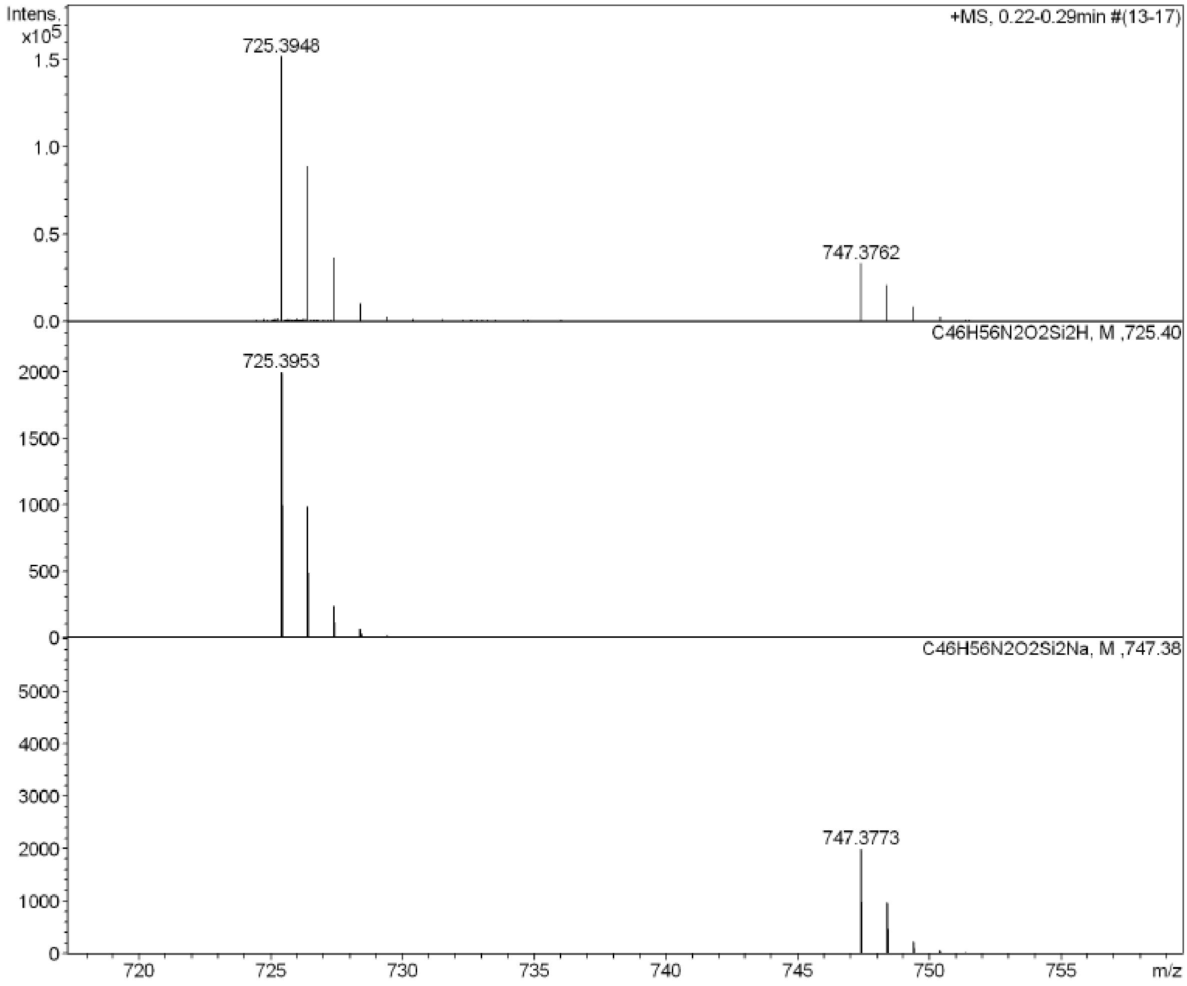

High Resolution Mass Spectrometry Report

Measured m/z vs. theoretical m/z

Meas. m/z	#	Formula	Score	m/z	err [mDa]	err [ppm]	mSigma	rdb	e⁻ Conf	z
725.3948	1	C 46 H 57 N 2 O 2 Si 2	100.00	725.3953	0.5	0.7	13.4	21.5	even	1+
747.3762	1	C 46 H 56 N 2 Na O 2 Si 2	100.00	747.3773	1.0	1.4	8.3	21.5	even	

Mass list

#	m/z	I %	I
1	173.0819	0.5	746
2	183.0808	1.0	1551
3	185.1177	4.7	7056
4	201.1046	1.1	1625
5	205.0621	5.5	8271
6	206.0657	0.5	717
7	215.1278	0.6	843
8	217.1069	1.2	1827
9	239.0903	0.7	1119
10	245.0800	0.6	844
11	261.1313	0.5	782
12	273.1683	1.5	2303
13	301.1406	0.8	1245
14	315.1921	0.8	1162
15	331.2087	1.1	1656
16	353.2661	0.6	841
17	381.2964	0.5	727
18	393.2962	0.5	703
19	413.2657	1.2	1895
20	433.1021	1.3	1968
21	441.3268	2.1	3191
22	442.3305	0.7	1013
23	447.3437	0.8	1248
24	463.3095	1.1	1684
25	464.3120	0.5	713
26	492.1175	0.6	843
27	633.3675	0.8	1191
28	634.3710	0.5	738
29	724.7456	0.6	865
30	725.1371	0.6	952
31	725.1740	0.6	856
32	725.2030	0.7	1061
33	725.2605	1.1	1614
34	725.3948	100.0	151630
35	725.6000	0.6	929
36	725.7152	0.5	829
37	725.7727	0.5	701
38	725.8332	0.5	803
39	725.9701	0.6	897
40	726.0008	0.8	1194
41	726.1554	0.5	712
42	726.2137	0.6	979
43	726.2630	0.6	852
44	726.3096	0.7	1018
45	726.3974	58.7	88953
46	727.0201	0.5	744
47	727.3977	24.1	36547
48	728.3974	6.7	10202
49	729.4002	1.6	2457
50	730.4005	0.5	832
51	731.5263	0.6	899
52	733.5301	0.5	726
53	747.3762	21.8	33109
54	748.3793	13.6	20647
55	749.3790	5.3	8023
56	750.3789	1.7	2541
57	763.3495	2.8	4246
58	764.3522	1.6	2398
59	765.3511	0.7	1081
60	787.3157	12.4	18761
61	788.3189	7.8	11812

#	m/z	I %	I
62	789.3155	8.0	12077
63	790.3167	4.2	6321
64	791.3178	1.7	2546
65	810.4834	0.6	842
66	826.5141	3.5	5284
67	827.5178	2.2	3269
68	828.5180	1.0	1529
69	907.5461	0.8	1174
70	908.5451	0.5	791
71	969.4646	4.0	6113
72	970.4669	3.3	4937
73	971.4647	3.4	5219
74	972.4657	2.1	3140
75	973.4659	1.1	1661
76	1199.4332	1.0	1458
77	1200.4396	0.8	1154
78	1201.4347	0.8	1144
79	1202.4365	0.7	986
80	1449.7796	1.2	1747
81	1450.7801	1.5	2305
82	1451.7818	1.1	1686
83	1452.7842	0.6	892
84	1466.8035	0.5	740
85	1467.8049	0.6	881
86	1471.7604	7.0	10628
87	1472.7633	8.6	13071
88	1473.7642	5.9	9020
89	1474.7670	3.1	4730
90	1475.7648	1.3	2009
91	1476.7703	0.5	732
92	1487.7342	0.5	785
93	1488.7365	0.7	999
94	1489.7403	0.6	949
95	1490.7417	0.5	774
96	1511.7025	1.7	2578
97	1512.6991	1.8	2719
98	1513.7007	1.8	2743
99	1514.7021	1.4	2137
100	1515.7046	0.9	1303

Acquisition Parameter

Source Type	ESI	Ion Polarity	Positive	Set Nebulizer	0.4 Bar
Focus	Not active	Set Capillary	3600 V	Set Dry Heater	180 °C
Scan Begin	75 m/z	Set End Plate Offset	-500 V	Set Dry Gas	4.0 l/min
Scan End	1700 m/z	Set Collision Cell RF	350.0 Vpp	Set Ion Energy (MS only)	4.0 eV

^{1}H-, ^{13}C-NMR (CD_2Cl_2, 400/101 MHz, 22 °C) and HR-ESI-MS spectra of compound (13)

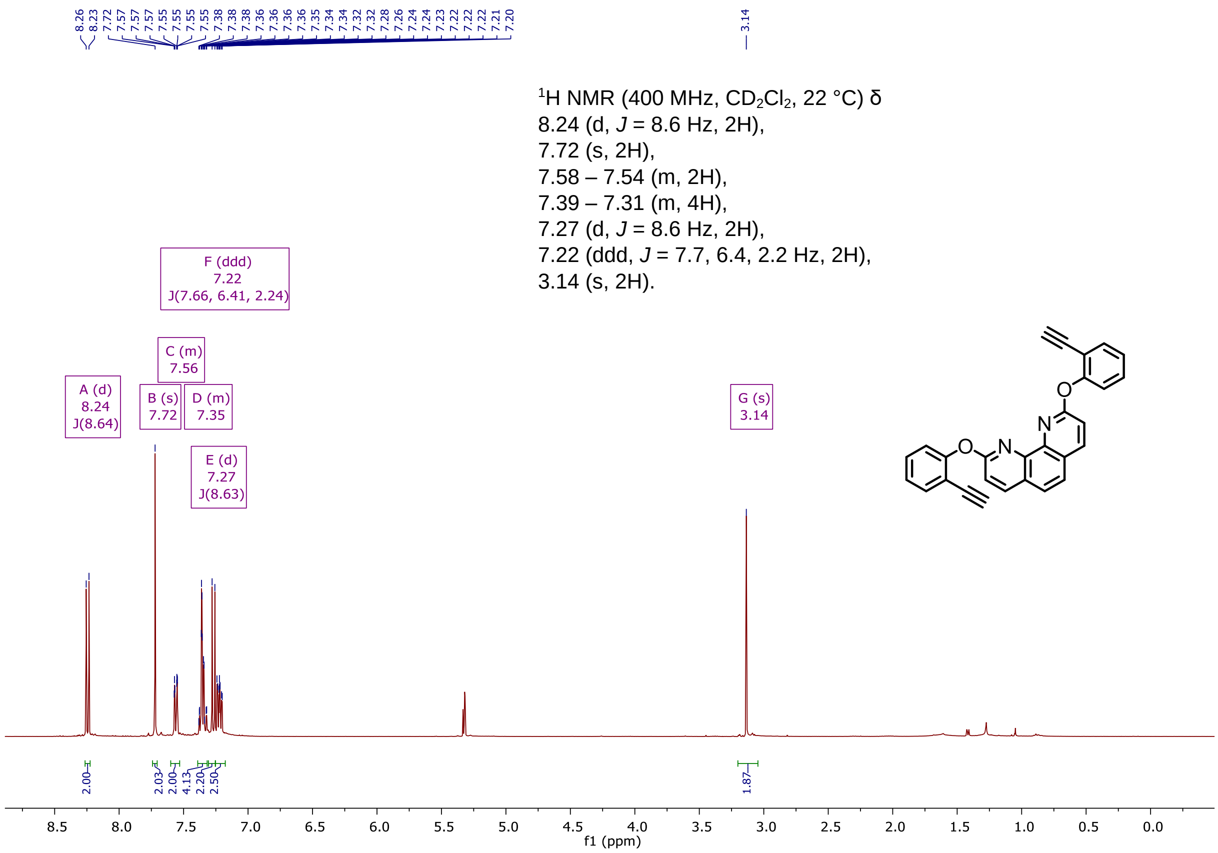

^{1}H NMR (400 MHz, CD_2Cl_2, 22 °C) δ
8.24 (d, *J* = 8.6 Hz, 2H),
7.72 (s, 2H),
7.58 – 7.54 (m, 2H),
7.39 – 7.31 (m, 4H),
7.27 (d, *J* = 8.6 Hz, 2H),
7.22 (ddd, *J* = 7.7, 6.4, 2.2 Hz, 2H),
3.14 (s, 2H).

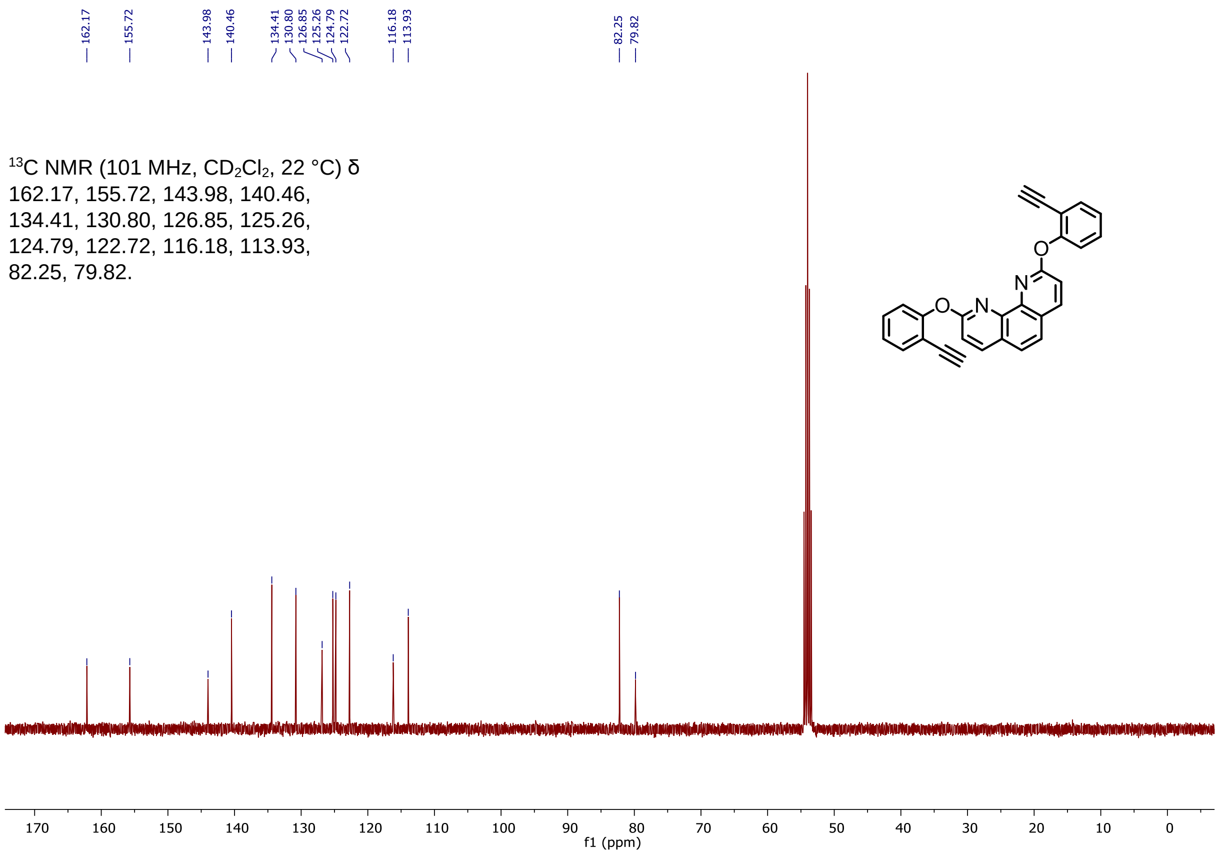

^{13}C NMR (101 MHz, CD_2Cl_2, 22 °C) δ
162.17, 155.72, 143.98, 140.46,
134.41, 130.80, 126.85, 125.26,
124.79, 122.72, 116.18, 113.93,
82.25, 79.82.

High Resolution Mass Spectrometry Report

Sample Name	**Thomas Brandl / BRT557**	Instrument	maXis 4G
Comment	10 ug/mL in MeCN, analyzed in MeCN	Method	22 Direct_pos_mid.m

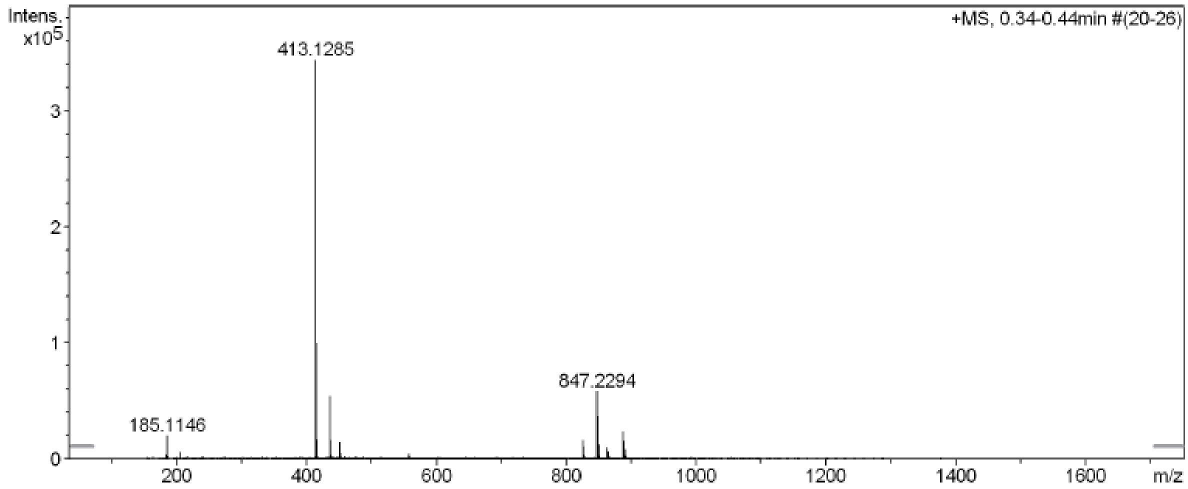

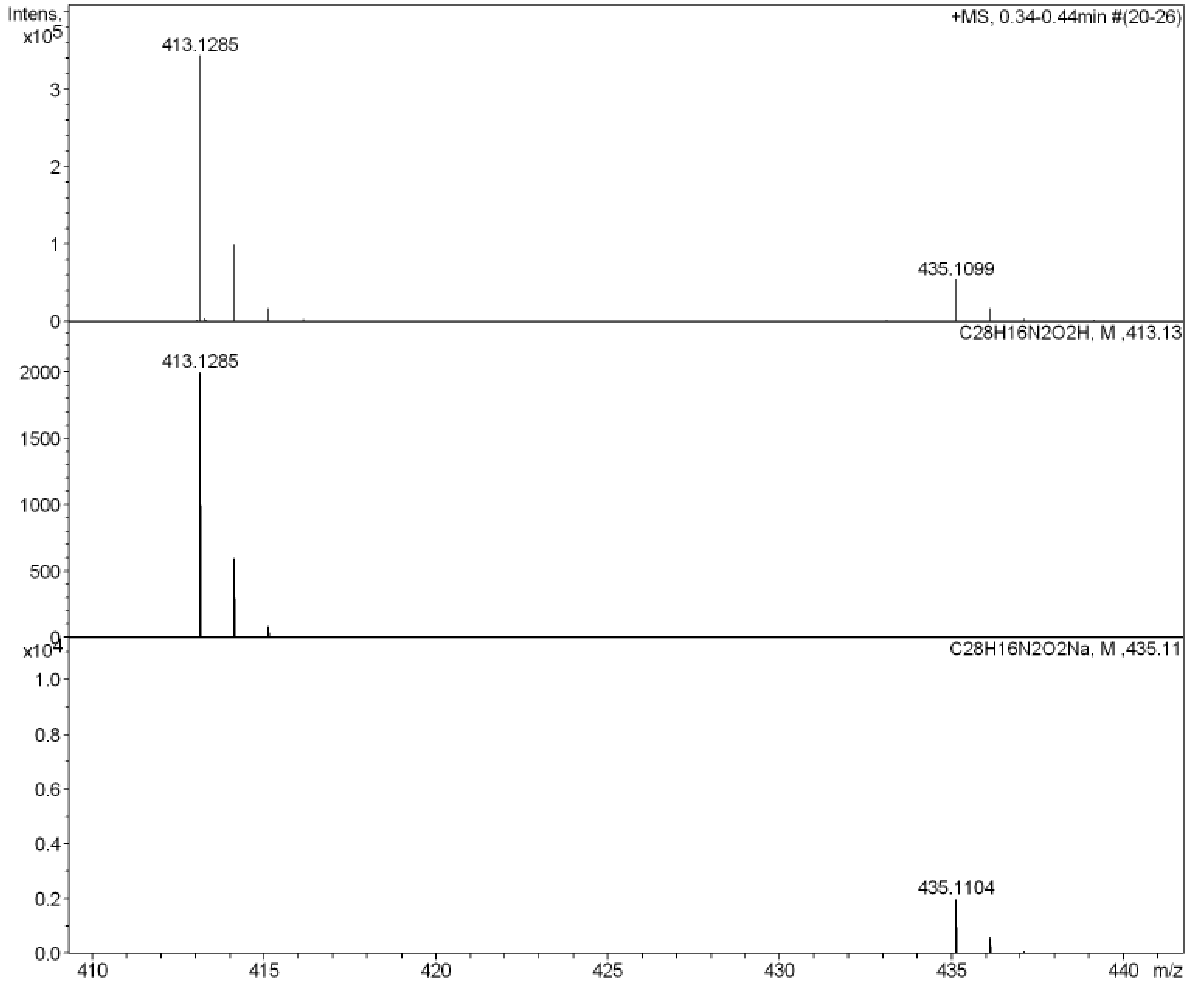

High Resolution Mass Spectrometry Report

Measured m/z vs. theoretical m/z

Meas. m/z	#	Formula	Score	m/z	err [mDa]	err [ppm]	mSigma	rdb	e⁻ Conf	z
413.1285	1	C 28 H 17 N 2 O 2	100.00	413.1285	-0.0	-0.0	10.4	21.5	even	1+
435.1099	1	C 28 H 16 N 2 Na O 2	100.00	435.1104	0.5	1.1	6.6	21.5	even	

Mass list

#	m/z	I %	I
1	155.0465	0.3	869
2	163.1327	0.4	1499
3	183.0778	1.0	3306
4	185.1146	5.8	19814
5	186.1176	0.6	2144
6	201.0879	0.3	934
7	201.1014	0.2	744
8	205.0596	1.5	5269
9	215.1244	0.3	1060
10	217.1049	0.5	1559
11	239.0884	0.4	1395
12	241.0681	0.3	877
13	273.1666	0.5	1667
14	301.1398	0.3	1102
15	315.1920	0.2	766
16	331.2085	0.5	1620
17	339.1772	0.2	750
18	353.2654	0.3	978
19	367.2085	0.2	685
20	381.2971	0.2	732
21	383.1409	0.2	809
22	389.2503	0.3	918
23	391.2837	0.3	868
24	393.2981	0.3	1000
25	405.1223	0.3	940
26	412.9777	0.2	712
27	412.9968	0.3	981
28	413.0072	0.2	710
29	413.0385	0.4	1415
30	413.0633	0.3	1081
31	413.1285	100.0	343404
32	413.2658	1.0	3517
33	413.3165	0.4	1261
34	413.3459	0.3	1094
35	413.4646	0.2	851
36	413.5640	0.2	745
37	414.0509	0.2	739
38	414.1314	29.0	99473
39	414.2691	0.3	1084
40	415.1344	4.7	16157
41	416.1368	0.6	1944
42	429.2401	0.3	1010
43	429.3182	0.2	753
44	433.1026	0.4	1362
45	435.1099	15.6	53462
46	436.1131	4.6	15910
47	437.1169	0.8	2768
48	439.1432	0.4	1449
49	441.2962	0.2	794
50	447.3436	0.5	1730
51	451.0837	4.0	13661
52	452.0863	1.3	4394
53	453.0832	0.5	1617
54	458.1859	0.4	1430
55	465.3690	0.2	739
56	475.0490	0.4	1405
57	477.0489	0.3	1130
58	486.1822	0.4	1259
59	514.2481	0.4	1447
60	556.4413	0.2	783
61	557.2066	1.1	3883

High Resolution Mass Spectrometry Report

#	m/z	I %	I
62	558.2099	0.6	2041
63	579.1878	0.2	730
64	601.4641	0.2	778
65	605.4145	0.3	996
66	621.4035	0.2	719
67	644.4918	0.2	775
68	659.5051	0.2	799
69	689.5128	0.2	730
70	691.4856	0.2	739
71	693.4632	0.2	769
72	717.5466	0.2	774
73	732.5433	0.2	688
74	733.5335	0.2	794
75	737.4938	0.2	693
76	749.5236	0.2	697
77	820.6010	0.2	700
78	825.2479	4.5	15602
79	826.2504	3.1	10508
80	827.2540	1.0	3317
81	828.2564	0.2	804
82	842.2762	0.2	763
83	847.2294	16.9	57975
84	848.2326	10.8	36952
85	849.2358	3.5	12080
86	849.6186	0.2	686
87	850.2393	0.7	2507
88	863.2027	2.8	9477
89	864.2058	1.7	5762
90	865.2073	0.7	2401
91	865.6126	0.2	684
92	866.2067	0.3	874
93	887.1685	6.7	23005
94	888.1717	4.4	15096
95	889.1692	4.2	14530
96	890.1712	2.2	7664
97	891.1729	0.7	2437
98	991.3062	0.3	890
99	992.3078	0.2	790
100	1053.7647	0.2	744

Acquisition Parameter

Source Type	ESI	Ion Polarity	Positive	Set Nebulizer	0.4 Bar
Focus	Not active	Set Capillary	3600 V	Set Dry Heater	180 °C
Scan Begin	75 m/z	Set End Plate Offset	-500 V	Set Dry Gas	4.0 l/min
Scan End	1700 m/z	Set Collision Cell RF	350.0 Vpp	Set Ion Energy (MS only)	4.0 eV

^{1}H-, ^{13}C-NMR (CD_2Cl_2, 500/126 MHz, 25 °C) and HR-ESI-MS spectra of compound (7)

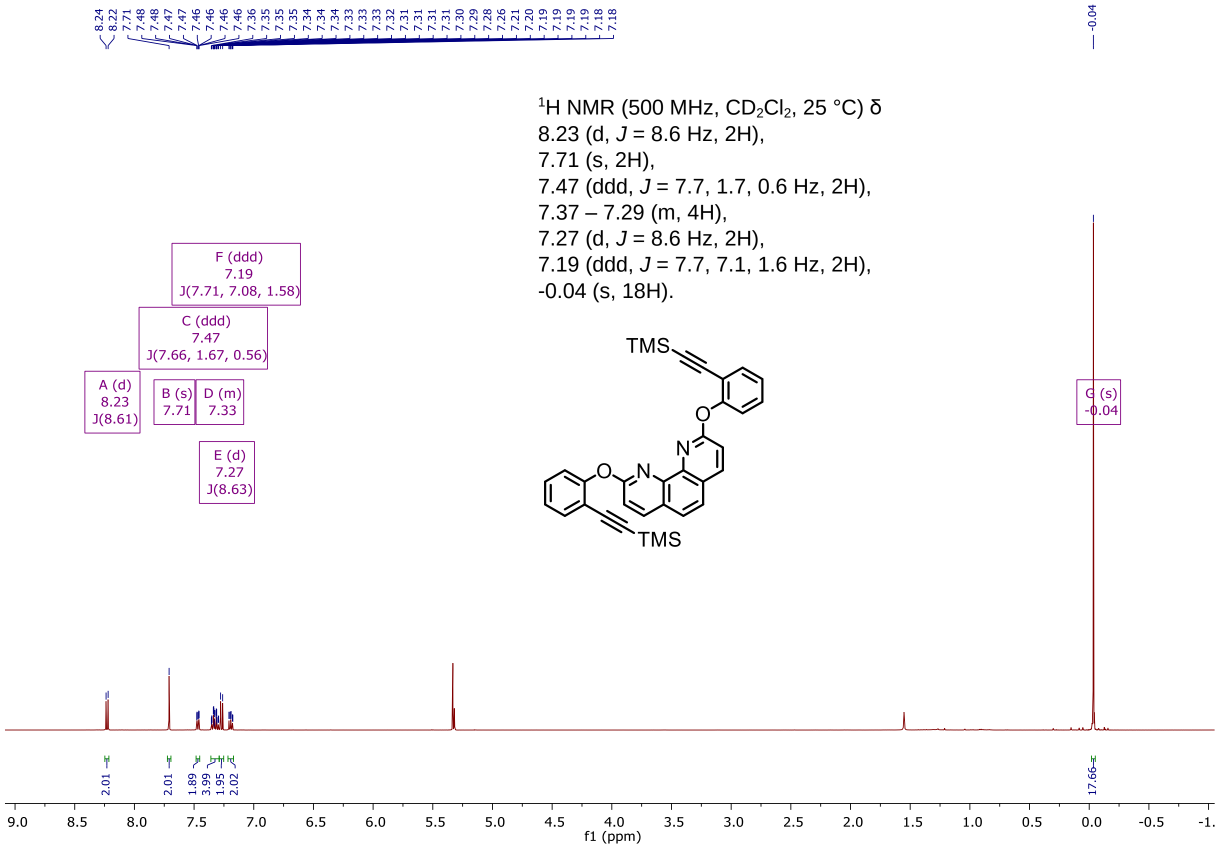

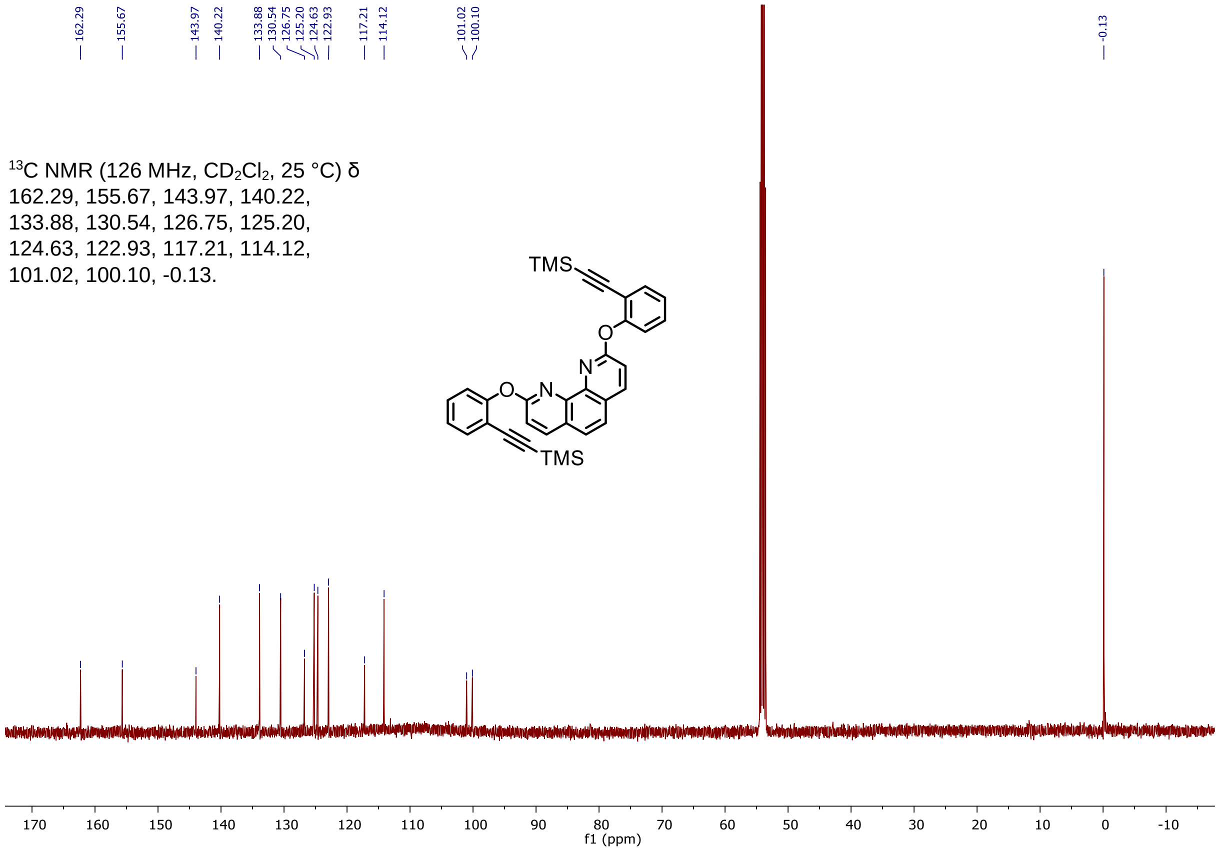

High Resolution Mass Spectrometry Report

Sample Name	**Thomas Brandl / BRT562**	Instrument	maXis 4G
Comment	10 ug/mL in MeCN, analyzed in MeCN	Method	22 Direct_pos_mid.m

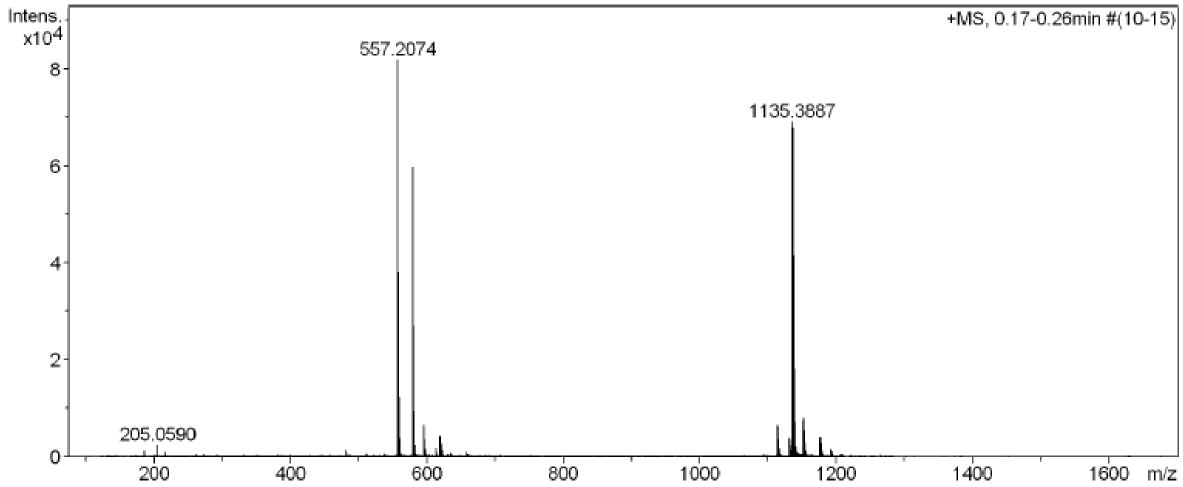

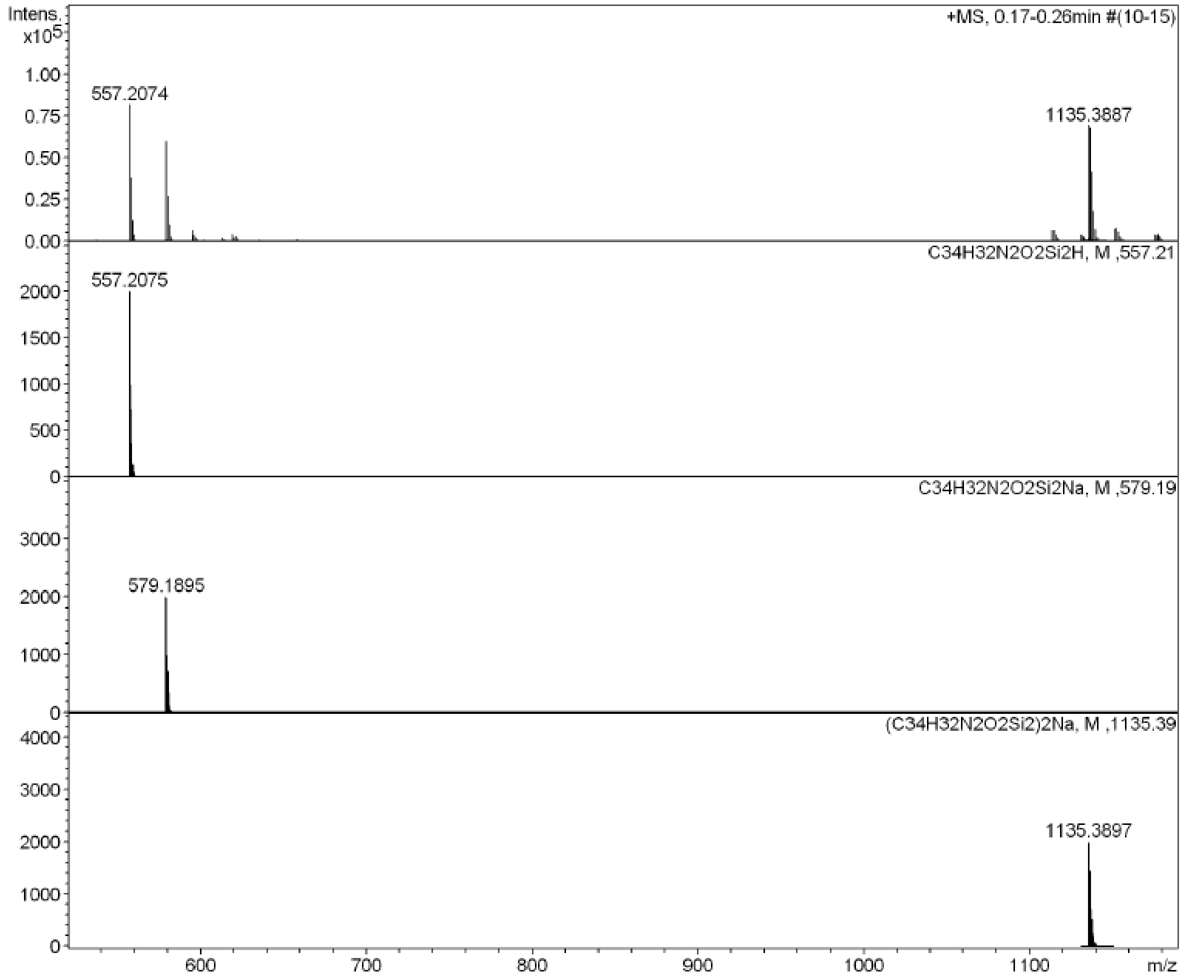

High Resolution Mass Spectrometry Report

Measured m/z vs. theoretical m/z

Meas. m/z	#	Formula	Score	m/z	err [mDa]	err [ppm]	mSigma	rdb	e⁻ Conf	z
557.2074	1	C 34 H 33 N 2 O 2 Si 2	100.00	557.2075	0.2	0.3	15.7	21.5	even	1+
579.1892	1	C 34 H 32 N 2 Na O 2 Si 2	100.00	579.1895	0.2	0.4	17.3	21.5	even	
1135.3887	1	C 68 H 64 N 4 Na O 4 Si 4	100.00	1135.3897	1.0	0.8	9.4	42.5	even	

Mass list

#	m/z	I %	I
1	185.1141	1.4	1145
2	205.0590	2.9	2385
3	217.1033	1.2	974
4	481.2651	1.6	1301
5	482.2685	0.9	767
6	511.1828	0.7	561
7	537.2347	0.7	590
8	557.0243	0.8	658
9	557.0511	0.8	642
10	557.2074	100.0	81731
11	558.2097	46.5	38035
12	559.2090	15.0	12286
13	560.2095	4.5	3719
14	561.2099	0.9	764
15	579.0813	1.0	837
16	579.1892	73.0	59633
17	580.1917	32.9	26925
18	581.1913	11.4	9342
19	582.1919	2.9	2362
20	583.1912	0.7	563
21	595.1632	7.9	6473
22	596.1655	4.3	3481
23	597.1645	1.9	1560
24	613.2700	2.2	1797
25	614.2725	1.1	894
26	619.1280	5.1	4152
27	620.1312	2.4	1997
28	621.1279	3.1	2557
29	622.1300	1.6	1267
30	635.2516	0.9	706
31	658.3281	1.1	911
32	1113.4061	7.9	6450
33	1114.4085	7.8	6344
34	1115.4099	4.4	3561
35	1116.4113	2.0	1670
36	1117.4132	0.8	633
37	1130.4335	4.6	3774
38	1131.4345	4.3	3509
39	1132.4357	2.8	2283
40	1133.4365	1.6	1338
41	1134.4389	0.8	614
42	1134.8237	0.8	682
43	1134.9893	0.8	628
44	1135.0372	1.3	1029
45	1135.1171	0.8	620
46	1135.1559	0.7	602
47	1135.2305	0.9	707
48	1135.3887	84.5	69061
49	1135.6702	1.0	856
50	1135.7597	0.7	560
51	1135.8048	0.9	752
52	1135.8518	1.0	823
53	1135.9065	0.7	578
54	1135.9484	1.5	1189
55	1135.9990	1.1	873
56	1136.0898	1.3	1045
57	1136.1395	1.0	803
58	1136.2080	0.9	708
59	1136.2642	1.0	808
60	1136.3909	82.9	67756

High Resolution Mass Spectrometry Report

#	m/z	I %	I
61	1136.5646	0.8	663
62	1136.7120	0.7	609
63	1136.7926	0.7	589
64	1136.8513	0.8	653
65	1136.9538	0.8	665
66	1136.9963	0.9	746
67	1137.0405	0.9	705
68	1137.1048	1.2	982
69	1137.1539	0.7	566
70	1137.1879	0.9	723
71	1137.3919	50.8	41483
72	1137.7360	0.8	645
73	1137.7918	0.9	722
74	1137.9280	0.8	640
75	1137.9921	0.8	627
76	1138.0886	1.0	785
77	1138.3923	22.1	18047
78	1138.9565	0.8	672
79	1139.3923	8.5	6954
80	1140.3922	2.5	2005
81	1141.4116	1.5	1209
82	1142.3358	0.8	650
83	1143.4555	0.9	771
84	1144.7480	0.7	579
85	1145.6986	0.7	584
86	1151.3617	9.0	7333
87	1152.3645	9.7	7952
88	1153.3636	6.6	5361
89	1154.3637	3.4	2813
90	1155.3683	1.5	1204
91	1156.3669	0.7	552
92	1175.3271	4.7	3856
93	1176.3321	4.3	3548
94	1177.3282	4.9	4027
95	1178.3306	3.3	2697
96	1179.3305	1.6	1341
97	1180.3260	0.7	563
98	1191.4500	1.6	1328
99	1192.4530	1.9	1519
100	1193.4540	1.3	1026

Acquisition Parameter

Source Type	ESI	Ion Polarity	Positive	Set Nebulizer	0.4 Bar
Focus	Not active	Set Capillary	3600 V	Set Dry Heater	180 °C
Scan Begin	75 m/z	Set End Plate Offset	-500 V	Set Dry Gas	4.0 l/min
Scan End	1700 m/z	Set Collision Cell RF	350.0 Vpp	Set Ion Energy (MS only)	4.0 eV

^{1}H-, ^{13}C-NMR (CD_2Cl_2, 400/101 MHz, 22 °C) and HR-ESI-MS spectra of compound (5)

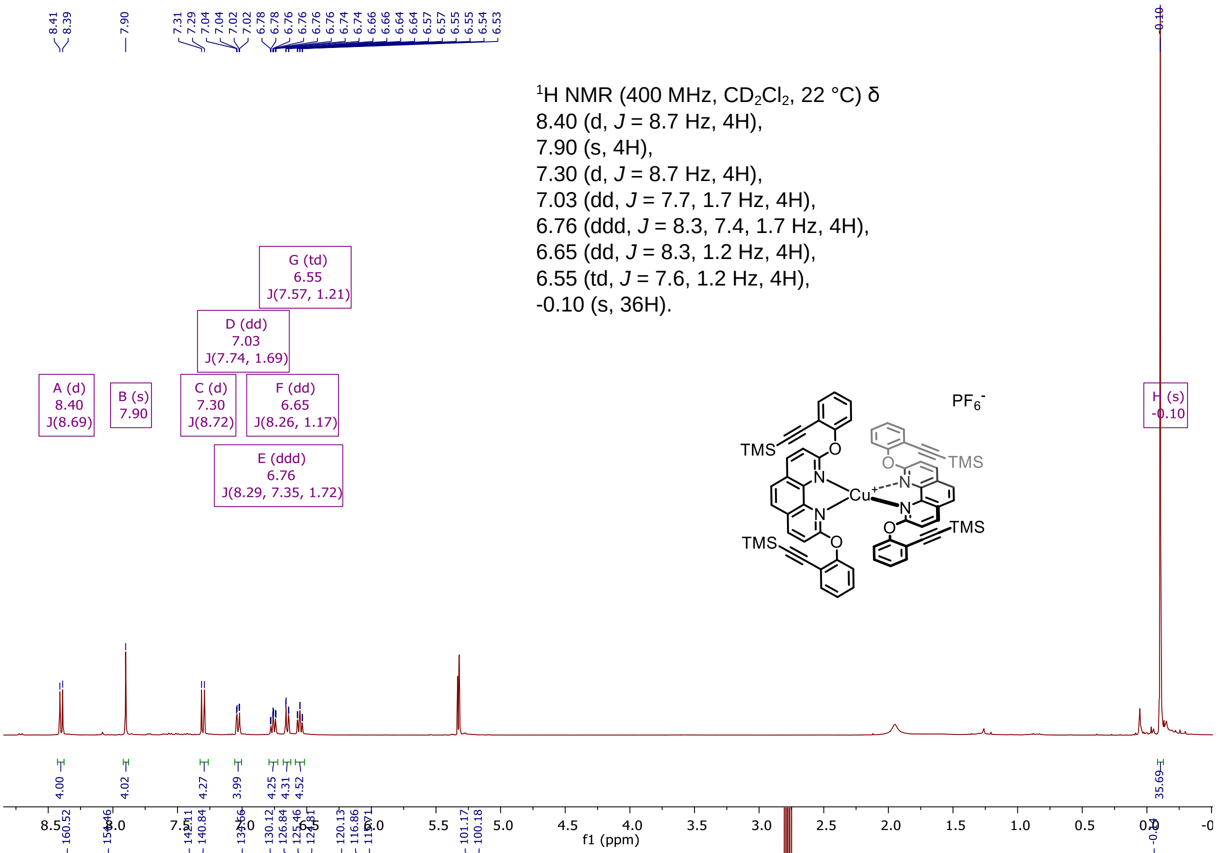

^{1}H NMR (400 MHz, CD_2Cl_2, 22 °C) δ 8.40 (d, J = 8.7 Hz, 4H), 7.90 (s, 4H), 7.30 (d, J = 8.7 Hz, 4H), 7.03 (dd, J = 7.7, 1.7 Hz, 4H), 6.76 (ddd, J = 8.3, 7.4, 1.7 Hz, 4H), 6.65 (dd, J = 8.3, 1.2 Hz, 4H), 6.55 (td, J = 7.6, 1.2 Hz, 4H), -0.10 (s, 36H).

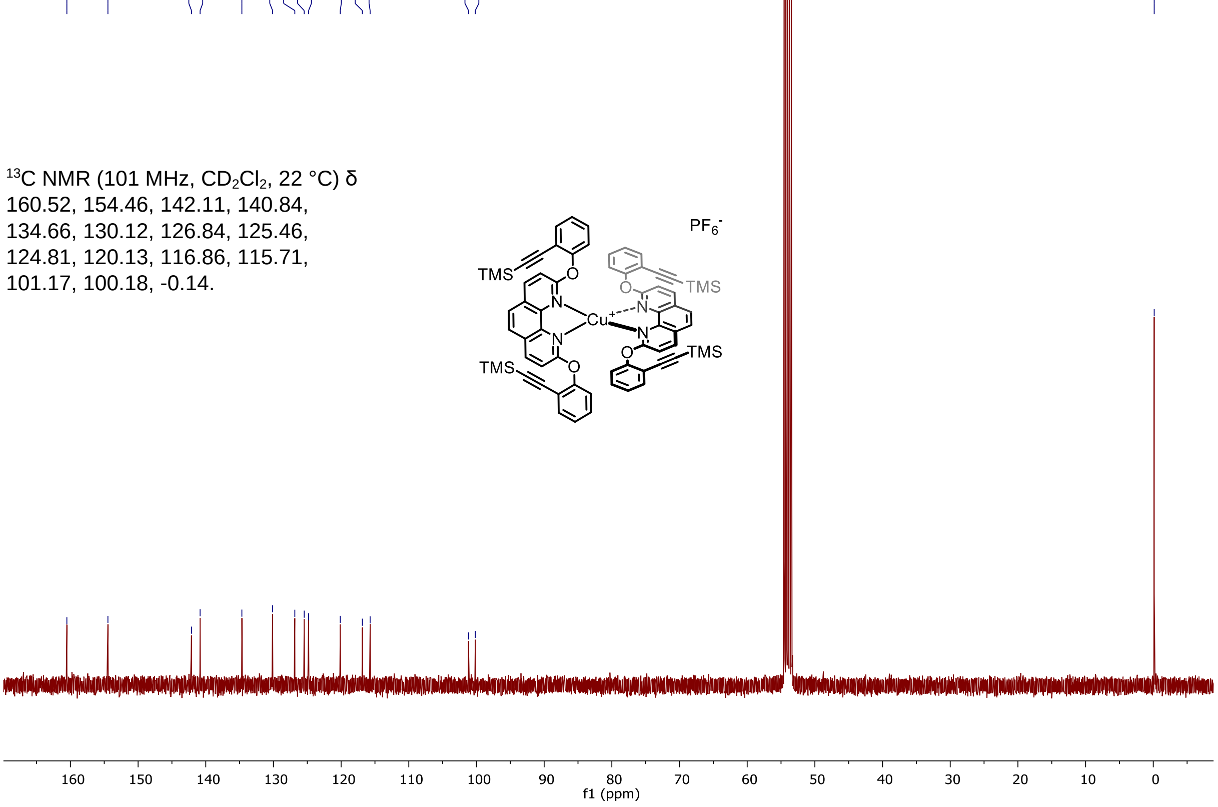

^{13}C NMR (101 MHz, CD_2Cl_2, 22 °C) δ 160.52, 154.46, 142.11, 140.84, 134.66, 130.12, 126.84, 125.46, 124.81, 120.13, 116.86, 115.71, 101.17, 100.18, -0.14.

High Resolution Mass Spectrometry Report

Sample Name	**Thomas Brandl / BRT563**	Instrument	maXis 4G
Comment	10 ug/mL in MeCN, analyzed in MeCN	Method	24 Direct_pos_high.m

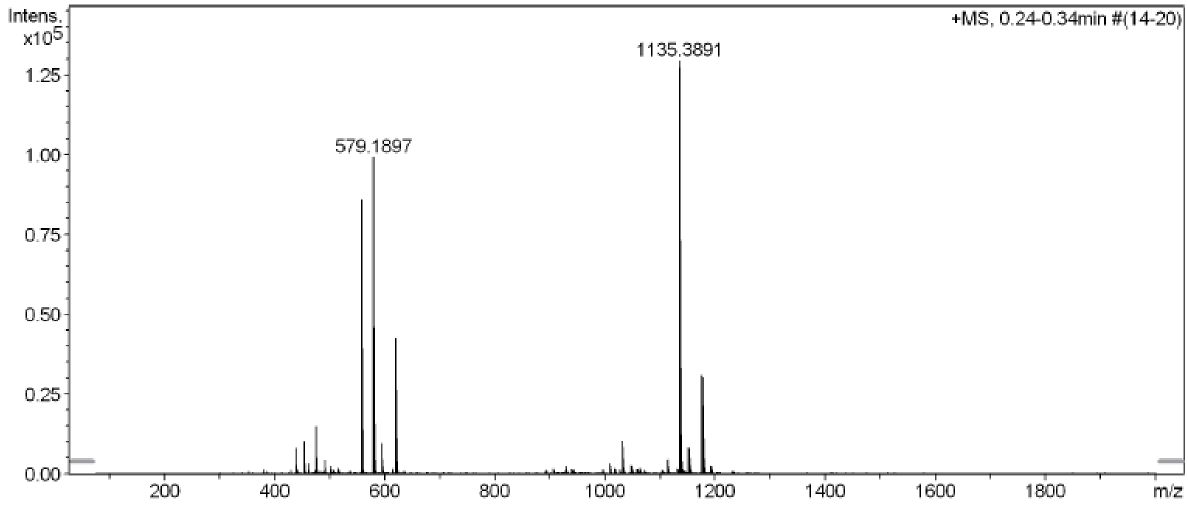

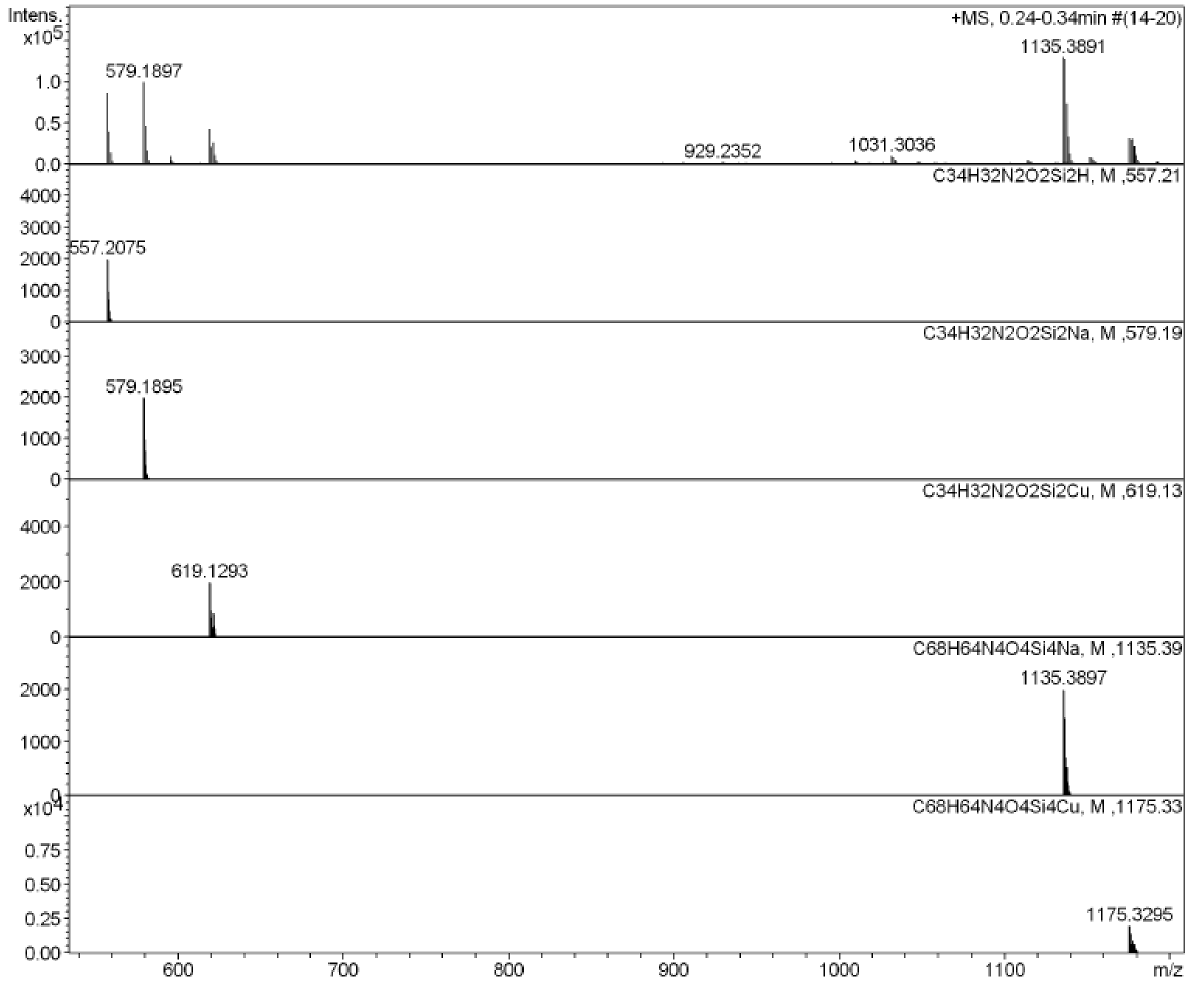

High Resolution Mass Spectrometry Report

Measured m/z vs. theoretical m/z

Meas. m/z	#	Formula	Score	m/z	err [mDa]	err [ppm]	mSigma	rdb	e⁻ Conf	z
557.2080	1	C 34 H 33 N 2 O 2 Si 2	100.00	557.2075	-0.5	-0.8	14.1	21.5	even	1+
579.1897	1	C 34 H 32 N 2 Na O 2 Si 2	100.00	579.1895	-0.2	-0.4	13.4	21.5	even	
619.1290	1	C 34 H 32 Cu N 2 O 2 Si 2	100.00	619.1293	0.3	0.5	5.7	21.5	even	
1135.3891	1	C 68 H 64 N 4 Na O 4 Si 4	100.00	1135.3897	0.5	0.5	15.7	42.5	even	
1175.3286	1	C 68 H 64 Cu N 4 O 4 Si 4	100.00	1175.3295	0.9	0.8	23.7	42.5	even	

Mass list

#	m/z	I %	I
1	381.2976	0.9	1214
2	429.3183	0.8	1088
3	439.1443	6.1	7939
4	440.1475	2.1	2662
5	443.2327	1.0	1242
6	453.1236	7.7	9936
7	454.1270	2.5	3246
8	461.1263	2.4	3151
9	462.1297	0.9	1123
10	475.1052	11.4	14718
11	476.1081	3.9	5077
12	477.1087	0.9	1140
13	491.0792	3.4	4341
14	492.0821	1.3	1731
15	501.0658	1.9	2465
16	503.0647	1.0	1275
17	515.0459	1.4	1767
18	517.0439	0.9	1112
19	557.2080	66.3	85739
20	558.2107	30.4	39299
21	559.2100	10.6	13724
22	560.2107	2.9	3711
23	579.1897	76.6	99061
24	580.1921	35.5	45884
25	581.1913	12.1	15607
26	582.1921	3.3	4232
27	595.1634	7.3	9382
28	596.1659	3.4	4386
29	597.1647	1.8	2284
30	613.2695	1.2	1538
31	619.1290	32.6	42191
32	620.1315	15.8	20471
33	621.1281	20.2	26158
34	622.1300	8.6	11129
35	623.1294	3.0	3920
36	624.1302	0.9	1152
37	893.2719	0.9	1116
38	905.2376	1.2	1581
39	929.2352	1.9	2500
40	930.2368	1.2	1602
41	939.2007	1.1	1424
42	943.2071	1.0	1267
43	995.3430	1.0	1269
44	1009.3239	2.5	3216
45	1010.3237	2.0	2575
46	1011.3252	1.1	1373
47	1017.3261	1.2	1524
48	1018.3276	1.1	1371
49	1026.3500	0.9	1200
50	1031.3036	7.9	10174
51	1032.3068	6.5	8413
52	1033.3078	3.6	4600
53	1034.3073	1.5	1877
54	1047.2785	2.1	2720
55	1048.2822	1.9	2485
56	1049.2823	1.3	1630
57	1057.2665	1.1	1412
58	1058.2674	1.0	1326

High Resolution Mass Spectrometry Report

#	m/z	I %	I
59	1059.2652	1.1	1428
60	1063.3477	1.3	1708
61	1064.3494	1.2	1598
62	1103.2866	0.9	1120
63	1113.4066	3.3	4309
64	1114.4087	3.4	4401
65	1115.4084	1.9	2492
66	1116.4108	0.9	1178
67	1130.4332	1.2	1561
68	1131.4380	1.1	1406
69	1132.4369	0.8	1061
70	1135.0618	1.2	1495
71	1135.1188	1.2	1568
72	1135.3891	100.0	129313
73	1135.7722	0.9	1141
74	1136.0442	0.8	1088
75	1136.1221	0.9	1114
76	1136.3916	98.2	126987
77	1136.8602	1.1	1378
78	1137.0542	1.0	1326
79	1137.2524	0.9	1152
80	1137.3925	56.6	73169
81	1138.0800	0.8	1069
82	1138.3928	25.5	32921
83	1139.3934	9.5	12296
84	1140.3933	3.0	3885
85	1141.3942	1.1	1389
86	1151.3618	6.3	8175
87	1152.3651	6.0	7784
88	1153.3644	3.8	4967
89	1154.3665	2.1	2755
90	1155.3625	1.0	1285
91	1175.3286	23.9	30889
92	1176.3309	23.0	29776
93	1177.3295	23.3	30072
94	1178.3299	16.4	21248
95	1179.3311	8.6	11074
96	1180.3311	3.6	4598
97	1181.3332	1.5	1878
98	1191.4497	1.9	2445
99	1192.4538	1.9	2500
100	1193.4542	1.2	1591

Acquisition Parameter

Source Type	ESI	Ion Polarity	Positive	Set Nebulizer	0.4 Bar
Focus	Not active	Set Capillary	3600 V	Set Dry Heater	180 °C
Scan Begin	75 m/z	Set End Plate Offset	-500 V	Set Dry Gas	4.0 l/min
Scan End	2000 m/z	Set Collision Cell RF	1000.0 Vpp	Set Ion Energy (MS only)	4.0 eV

^{1}H-, ^{13}C-NMR (CD_2Cl_2, 500/126 MHz, 25 °C) and HR-ESI-MS spectra of compound (3)

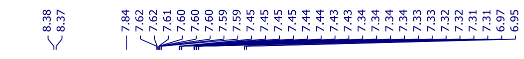

^{1}H NMR (500 MHz, CD_2Cl_2, 25 °C) δ
8.38 (d, J = 8.9 Hz, 4H),
7.84 (s, 4H),
7.60 (ddd, J = 8.2, 7.5, 1.7 Hz, 4H),
7.44 (ddd, J = 7.6, 1.7, 0.5 Hz, 4H),
7.35 – 7.30 (m, 8H),
6.96 (d, J = 8.9 Hz, 4H).

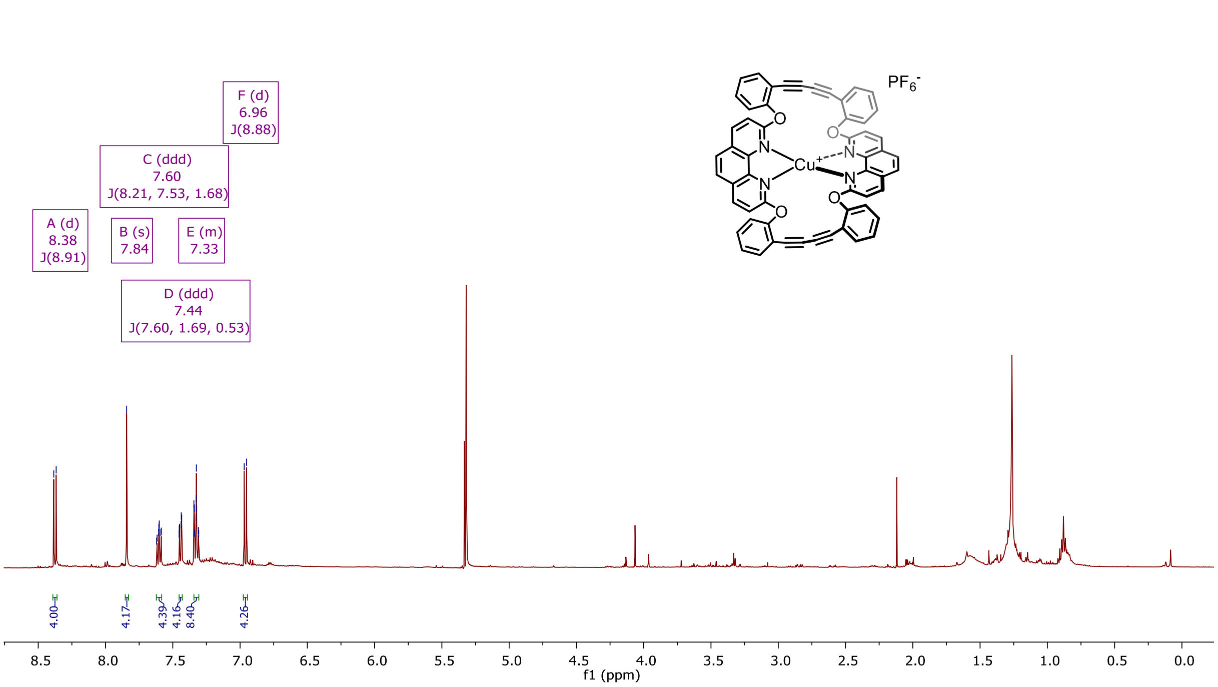

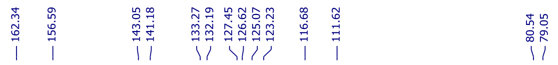

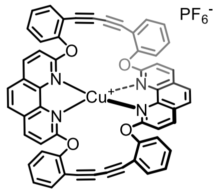

^{13}C NMR (126 MHz, CD_2Cl_2, 25 °C) δ
162.34, 156.59, 143.05, 141.18,
133.27, 132.19, 127.45, 126.62,
125.07, 123.23, 116.68, 111.62,
80.54, 79.05.

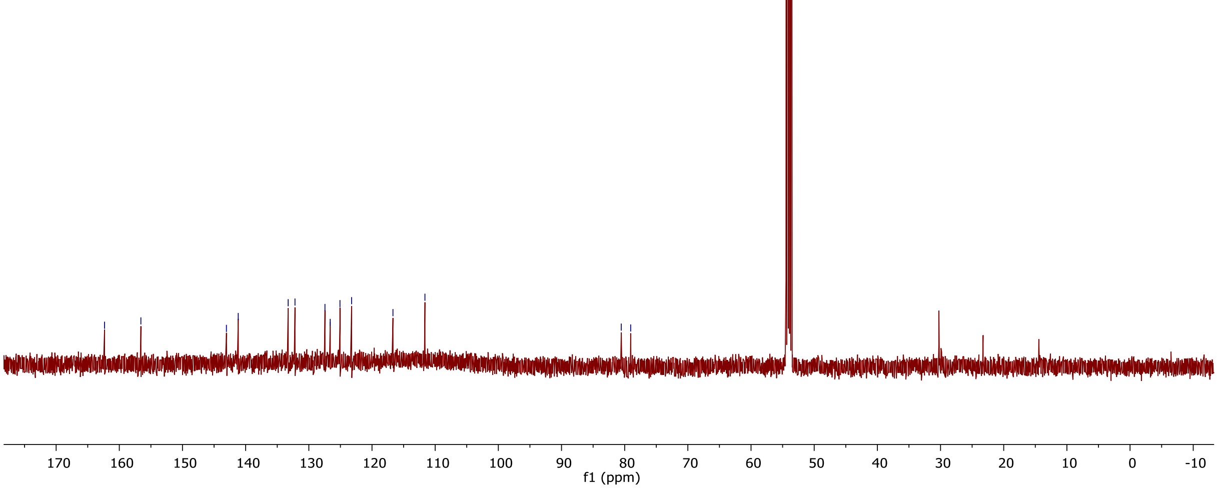

High Resolution Mass Spectrometry Report

Sample Name	**Thomas Brandl / BRT593**	Instrument	maXis 4G
Comment	10 ug/mL in MeCN, analyzed in MeCN	Method	24 Direct_pos_high.m

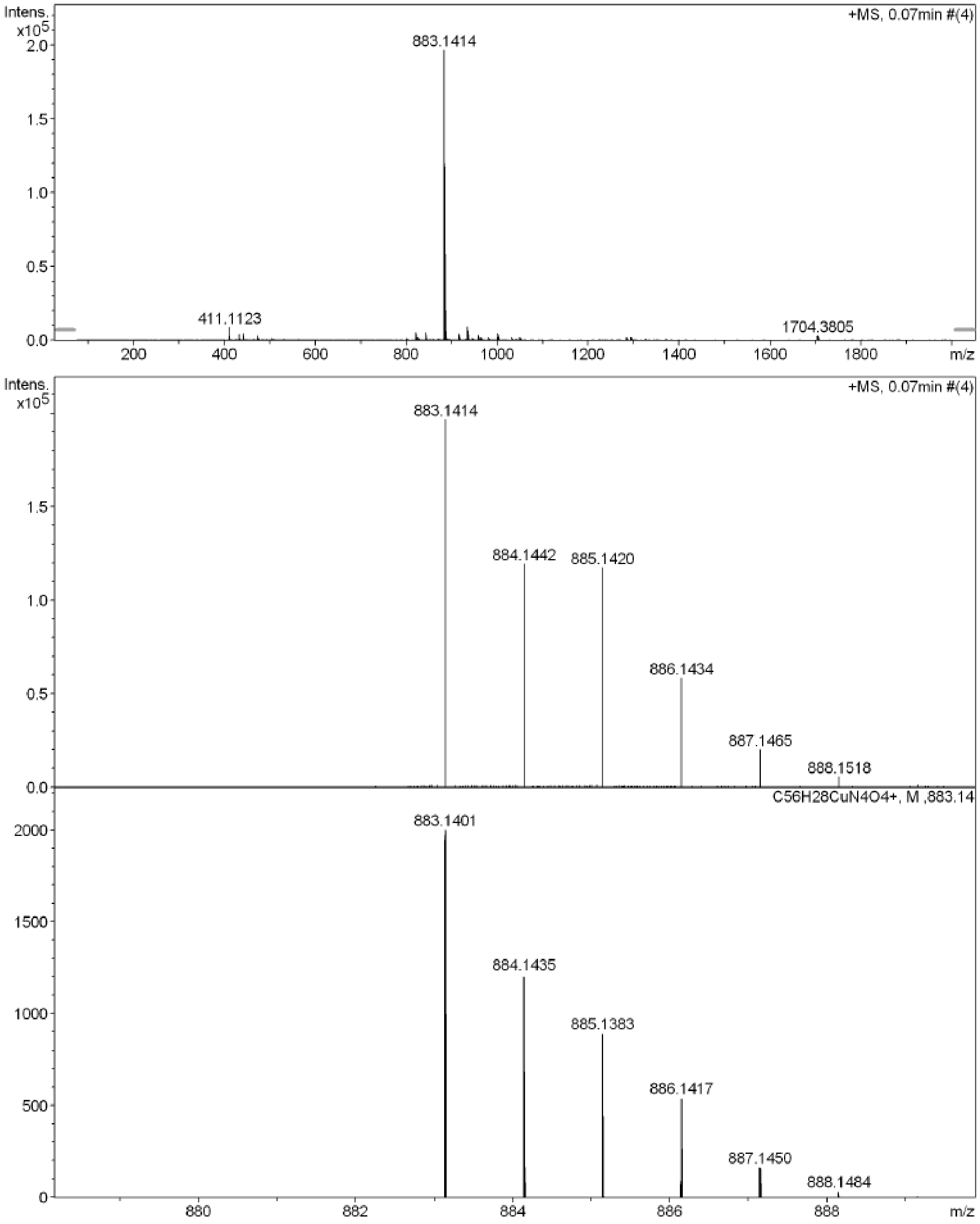

High Resolution Mass Spectrometry Report

Measured m/z vs. theoretical m/z

Meas. m/z	#	Formula	Score	m/z	err [mDa]	err [ppm]	mSigma	rdb	e⁻ Conf	z
883.1414	1	C 56 H 28 Cu N 4 O 4	100.00	883.1401	-1.3	-1.5	22.0	44.5	even	1+

Mass list

#	m/z	I %	I
1	411.1123	4.5	8741
2	412.1158	1.3	2542
3	433.0947	1.9	3726
4	434.0977	0.5	1000
5	441.5695	2.3	4458
6	442.0705	1.2	2312
7	442.5706	1.4	2758
8	443.0712	0.7	1360
9	443.1393	0.8	1520
10	473.0348	1.5	2912
11	474.0362	0.6	1150
12	475.0335	0.6	1192
13	505.0605	0.6	1160
14	531.1529	0.5	1006
15	799.1393	0.6	1144
16	821.2188	2.6	5022
17	822.2214	2.0	3856
18	823.2252	0.8	1502
19	825.1913	0.9	1817
20	826.1964	0.5	992
21	827.1898	0.6	1124
22	843.2005	2.6	5114
23	844.2026	1.7	3248
24	845.2102	0.7	1404
25	869.2203	0.6	1093
26	875.2214	0.4	762
27	882.9639	0.5	900
28	882.9698	0.5	915
29	883.0391	0.6	1088
30	883.1414	100.0	196422
31	883.2672	0.4	760
32	883.4329	0.4	779
33	883.5111	0.4	770
34	883.6006	0.4	765
35	883.6339	0.4	757
36	883.8780	0.4	782
37	883.9504	0.4	878
38	884.0308	0.5	984
39	884.0555	0.4	837
40	884.1442	60.7	119318
41	884.6768	0.4	864
42	884.7062	0.4	831
43	885.0040	0.5	885
44	885.0695	0.6	1224
45	885.1420	59.7	117262
46	886.1434	29.7	58382
47	887.1465	10.2	20037
48	888.1518	2.8	5574
49	889.1515	0.6	1172
50	915.1664	2.2	4242
51	916.1682	1.5	2859
52	917.1683	1.6	3184
53	918.1693	0.8	1512
54	919.1757	0.4	772
55	933.1777	4.5	8814
56	934.1809	3.2	6239
57	935.1787	3.0	5920
58	936.1802	1.8	3541
59	937.1871	0.4	856
60	945.1768	0.6	1120
61	946.1798	0.5	895
62	947.1861	0.5	1064

#	m/z	I %	I
63	957.1949	1.7	3250
64	958.1980	1.4	2829
65	959.1976	1.0	1987
66	960.1965	0.7	1306
67	961.2031	0.4	776
68	963.1873	0.8	1512
69	964.1933	0.6	1183
70	965.1892	0.6	1252
71	981.1990	1.0	1986
72	982.1988	0.5	918
73	983.2047	0.5	993
74	999.1664	0.9	1762
75	1000.1691	0.7	1343
76	1001.1819	2.3	4596
77	1002.1845	1.7	3346
78	1003.1865	1.5	2901
79	1004.1869	1.0	2025
80	1031.1964	0.9	1824
81	1032.1962	0.7	1313
82	1033.1945	0.6	1104
83	1034.2013	0.4	858
84	1041.2902	0.4	868
85	1049.2061	1.0	1966
86	1050.2078	0.7	1322
87	1051.2084	0.5	1054
88	1052.2059	0.5	958
89	1283.3240	0.9	1760
90	1284.3323	0.8	1531
91	1285.3300	0.7	1328
92	1293.2536	0.9	1812
93	1294.2558	0.9	1760
94	1295.2590	0.8	1628
95	1296.2613	0.6	1185
96	1703.3798	1.3	2501
97	1704.3805	1.5	2988
98	1705.3830	1.2	2450
99	1706.3865	1.1	2101
100	1707.3877	0.6	1262

Acquisition Parameter

**General**	Fore Vacuum<br>Scan Begin	2.68e+000 mBar<br>75 m/z	High Vacuum<br>Scan End	9.53e-008 mBar<br>2000 m/z	Source Type<br>Ion Polarity	ESI<br>Positive
**Source**	Set Nebulizer<br>Set Dry Heater	0.4 Bar<br>180 °C	Set Capillary<br>Set End Plate Offset	3600 V<br>-500 V	Set Dry Gas	4.0 l/min
**Quadrupole**	Set Ion Energy (MS only)	4.0 eV				
**Coll. Cell**	Collision Energy	10.0 eV	Set Collision Cell RF	1000.0 Vpp		
**Ion Cooler**	Set Ion Cooler Transfer Time	160.0 µs	Set Ion Cooler Pre Pulse Storage Time	18.0 µs		

^{1}H-, ^{13}C-NMR ($CDCl_3$, 400/101 MHz, 22 °C) and HR-ESI-MS spectra of compound (14)

^{1}H NMR (400 MHz, $CDCl_3$, 22 °C) δ
8.19 (d, J = 8.6 Hz, 2H),
7.68 (s, 2H),
7.40 – 7.29 (m, 8H),
7.24 – 7.17 (m, 4H).

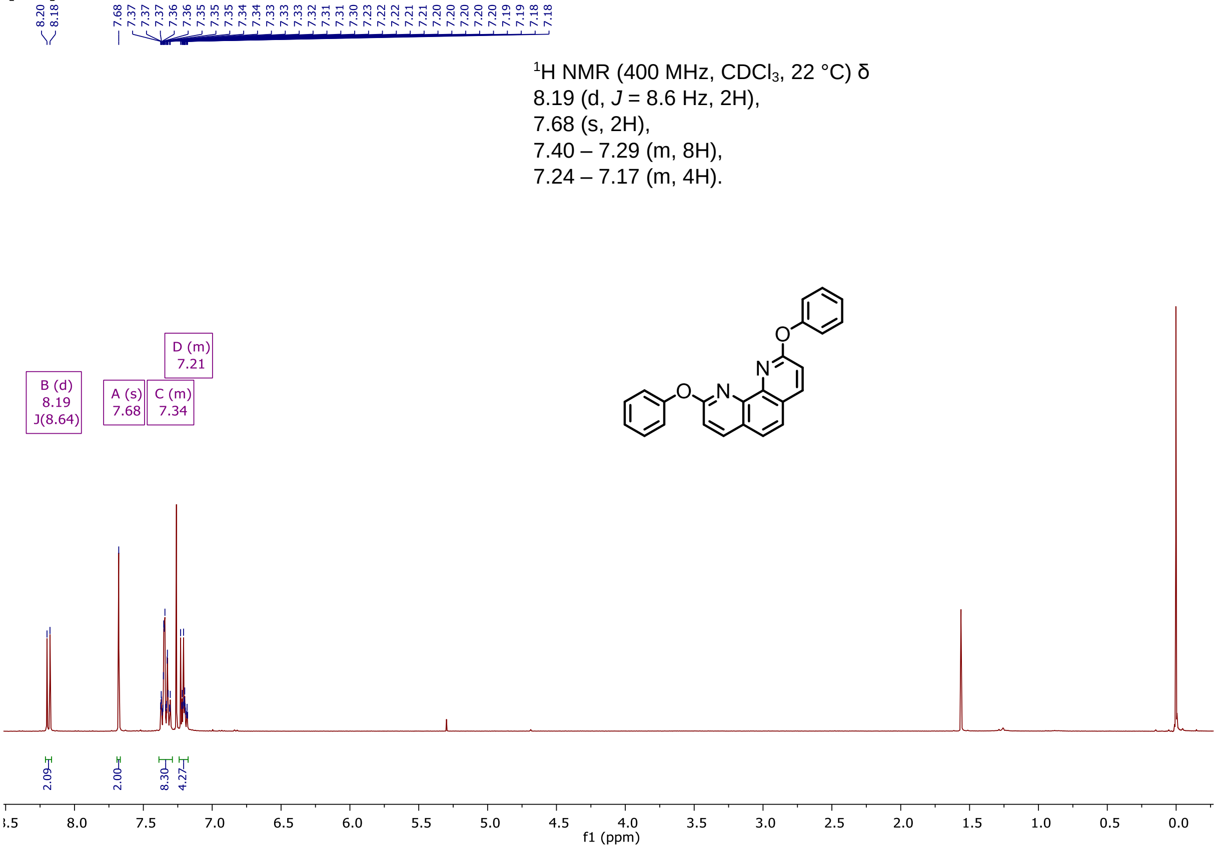

^{13}C NMR (101 MHz, $CDCl_3$, 22 °C) δ
162.06, 154.13, 143.69, 139.83,
129.64, 126.19, 124.55, 124.23,
121.17, 113.88.

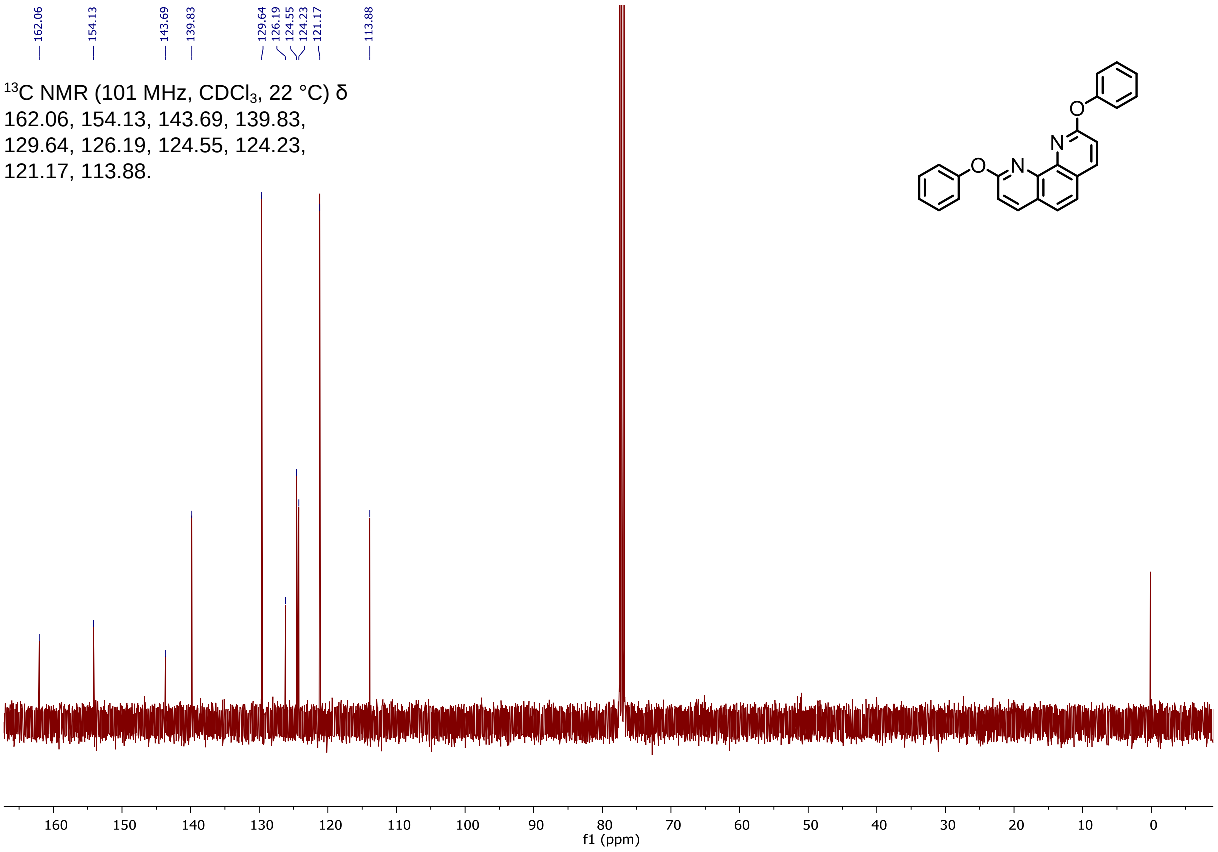

High Resolution Mass Spectrometry Report

Sample Name	**Thomas Brandl / BRT620**	Instrument	maXis 4G
Comment	10 ug/mL in DCM, analyzed in MeOH	Method	1 CALIB_702.m

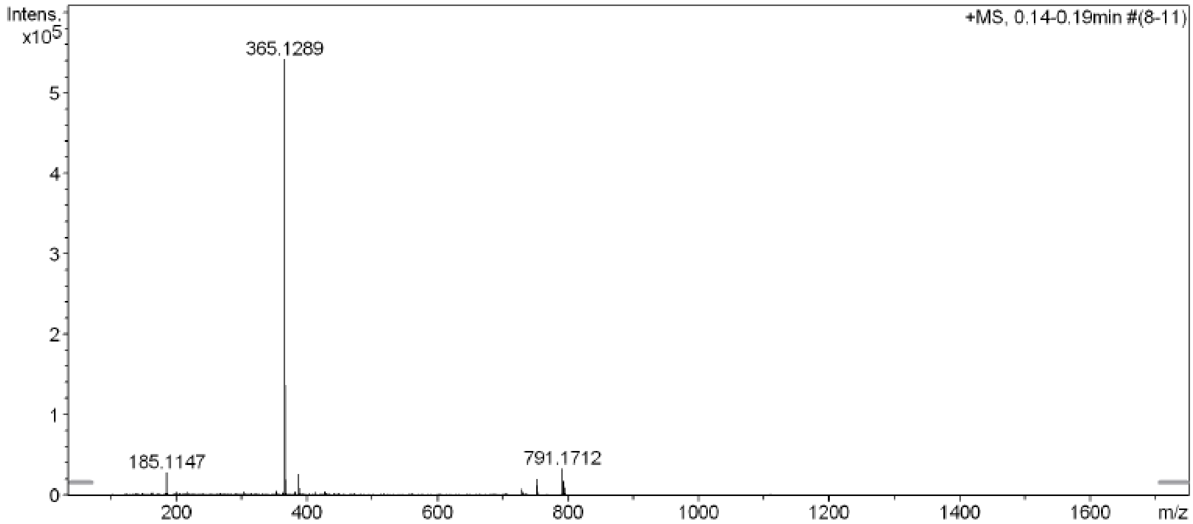

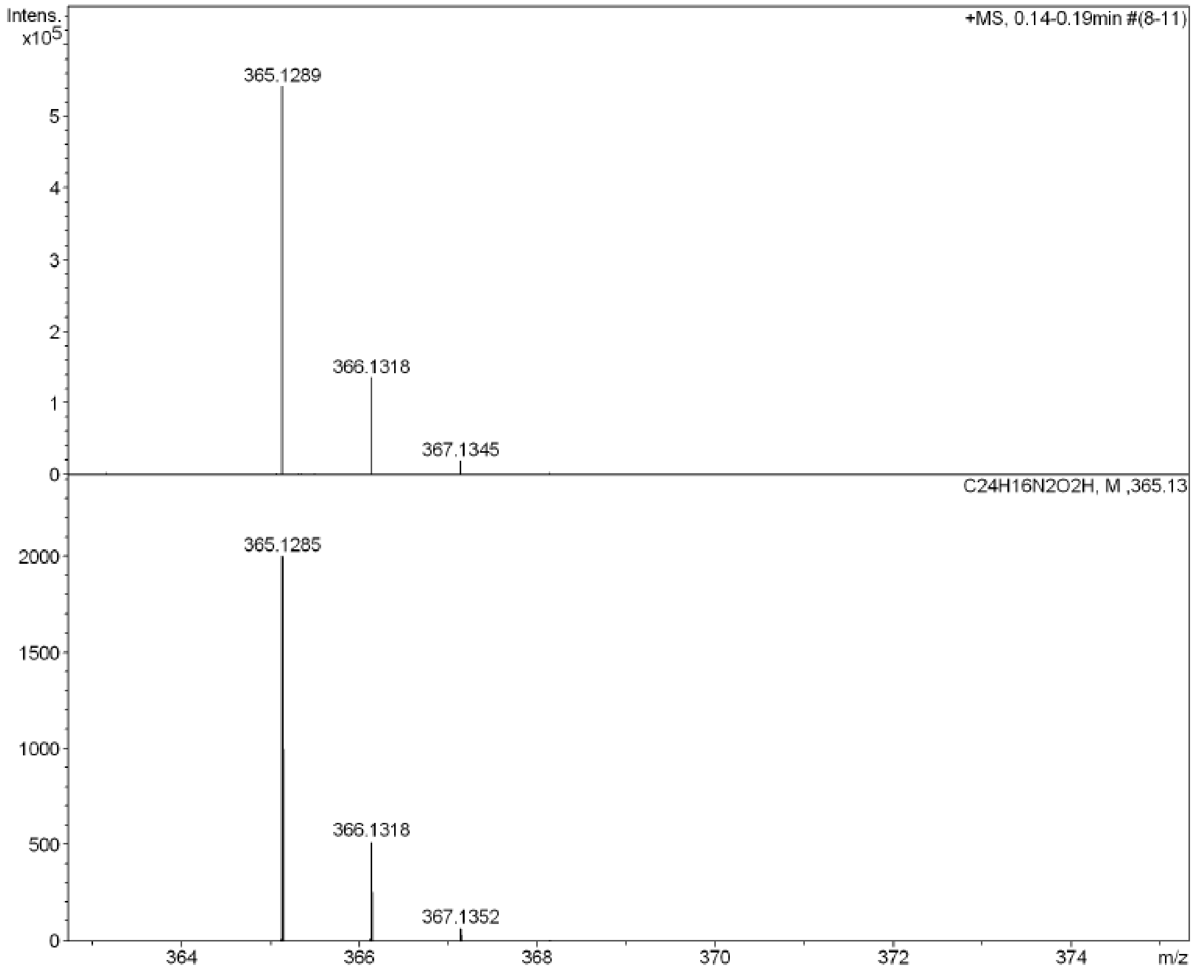

Measured m/z vs. theoretical m/z

Meas. m/z	#	Formula	Score	m/z	err [mDa]	err [ppm]	mSigma	rdb	e⁻ Conf	z
365.1289	1	C 24 H 17 N 2 O 2	100.00	365.1285	-0.5	-1.3	9.3	17.5	even	1+

Mass list

#	m/z	I %	I
1	123.0917	0.3	1504
2	137.1072	0.3	1575
3	138.0910	0.2	1351
4	139.0502	0.2	960
5	140.9616	0.3	1541
6	147.0914	0.4	1915
7	149.0232	0.3	1581
8	161.1065	0.2	1315
9	163.1327	0.6	3063
10	169.0467	0.2	1023
11	173.0784	0.4	2393
12	183.0777	0.4	2007
13	183.0998	0.2	1131
14	185.1147	5.2	27902
15	186.1182	0.4	2376
16	197.0782	0.2	1200
17	201.1023	0.7	3532
18	205.0598	0.4	1929
19	211.0937	0.2	1231
20	215.1254	0.2	1087
21	217.1044	0.7	3888
22	219.0476	0.2	1348
23	225.1094	0.2	1068
24	241.0294	0.2	1227
25	251.1613	0.3	1564
26	261.1299	0.3	1390
27	265.1763	0.2	1128
28	267.1569	0.4	1936
29	273.1671	0.3	1375
30	273.1915	0.3	1633
31	279.2284	0.2	1117
32	283.1512	0.2	1073
33	291.1932	0.2	1233
34	293.2085	0.3	1496
35	303.1779	0.8	4357
36	304.2997	0.2	1152
37	305.2086	0.3	1659
38	309.2048	0.2	1032
39	315.1925	0.3	1813
40	319.2244	0.3	1505
41	321.2035	0.2	1071
42	331.2086	0.3	1443
43	339.1784	0.2	988
44	353.1453	0.3	1788
45	353.2660	0.9	4674
46	354.2696	0.2	1078
47	363.1490	0.5	2722
48	365.0678	0.3	1594
49	365.1289	100.0	541132
50	365.2780	0.2	1202
51	365.3184	0.3	1513
52	365.3497	0.2	1224
53	365.3859	0.2	1183
54	365.4053	0.2	1141
55	365.4422	0.2	1102
56	365.4964	0.2	1288
57	366.1318	25.1	135939
58	367.1345	3.5	18989
59	368.1369	0.4	2280
60	381.2976	0.7	3964
61	382.3012	0.2	1328
62	385.2925	0.2	1025

#	m/z	I %	I
63	387.1102	4.7	25256
64	388.1132	1.4	7551
65	389.1160	0.2	1185
66	391.2846	0.2	1211
67	393.2962	0.2	1100
68	403.0837	0.3	1646
69	413.2656	0.7	3684
70	414.2691	0.2	969
71	427.0498	0.9	4613
72	427.2086	0.8	4227
73	428.0536	0.2	1228
74	428.2116	0.2	1164
75	429.0484	0.4	2302
76	429.3179	0.3	1568
77	433.1033	0.3	1647
78	441.3275	0.2	1315
79	447.3440	0.3	1807
80	449.3748	0.2	1327
81	473.3436	0.3	1781
82	487.3602	0.2	1043
83	512.4154	0.2	1003
84	517.3703	0.2	1265
85	556.4406	0.2	1047
86	561.3981	0.2	1318
87	700.6284	0.2	1098
88	705.5813	0.4	2082
89	729.2486	1.3	6950
90	730.2529	0.7	3793
91	731.2530	0.2	1153
92	735.2561	0.2	1220
93	751.2313	3.6	19695
94	752.2341	2.1	11427
95	753.2376	0.6	3224
96	791.1712	6.1	32904
97	792.1740	3.2	17222
98	793.1708	3.2	17290
99	794.1727	1.6	8607
100	795.1748	0.4	2187

Acquisition Parameter

**General**	Fore Vacuum	2.79e+000 mBar	High Vacuum	1.01e-007 mBar	Source Type	ESI
	Scan Begin	75 m/z	Scan End	1700 m/z	Ion Polarity	Positive
**Source**	Set Nebulizer	0.4 Bar	Set Capillary	3600 V	Set Dry Gas	3.0 l/min
	Set Dry Heater	180 °C	Set End Plate Offset	-500 V		
**Quadrupole**	Set Ion Energy (MS only)	4.0 eV				
**Coll. Cell**	Collision Energy	8.0 eV	Set Collision Cell RF	350.0 Vpp		
**Ion Cooler**	Set Ion Cooler Transfer Time	55.0 µs	Set Ion Cooler Pre Pulse Storage Time	7.0 µs		

^{1}H-, ^{13}C-NMR (CD_2Cl_2, 400/101 MHz, 22 °C) and HR-ESI-MS spectra of compound (15)

^{1}H NMR (400 MHz, CD_2Cl_2, 22 °C) δ
8.43 (d, J = 8.7 Hz, 4H),
7.91 (s, 4H),
7.29 (d, J = 8.7 Hz, 4H),
6.94 – 6.87 (m, 8H),
6.77 – 6.69 (m, 4H),
6.66 – 6.59 (m, 8H).

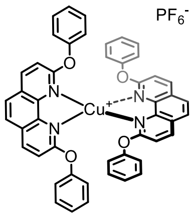

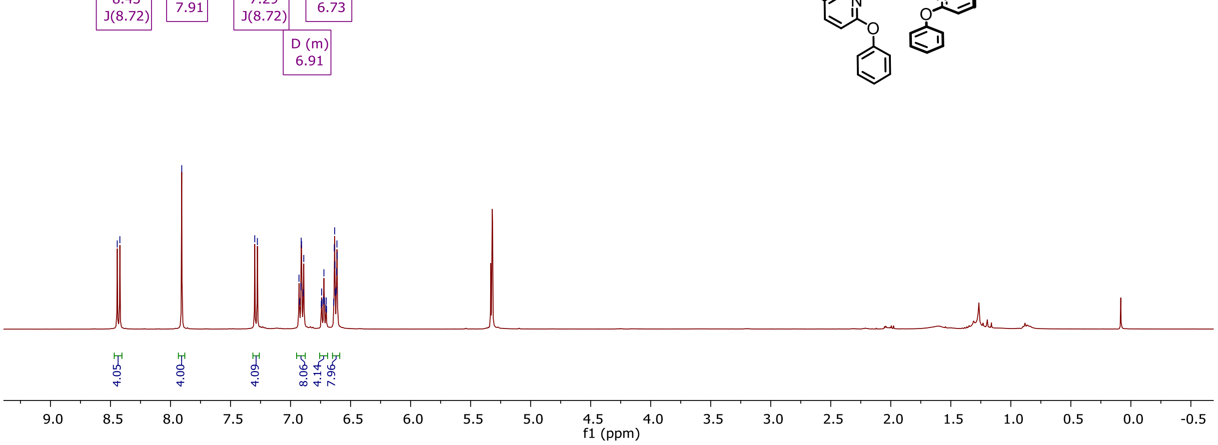

^{13}C NMR (101 MHz, CD_2Cl_2, 22 °C) δ
160.31, 153.69, 141.95, 140.47,
129.47, 126.20, 124.78, 124.55,
118.90, 115.06.

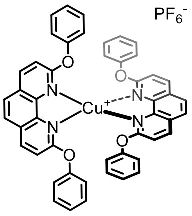

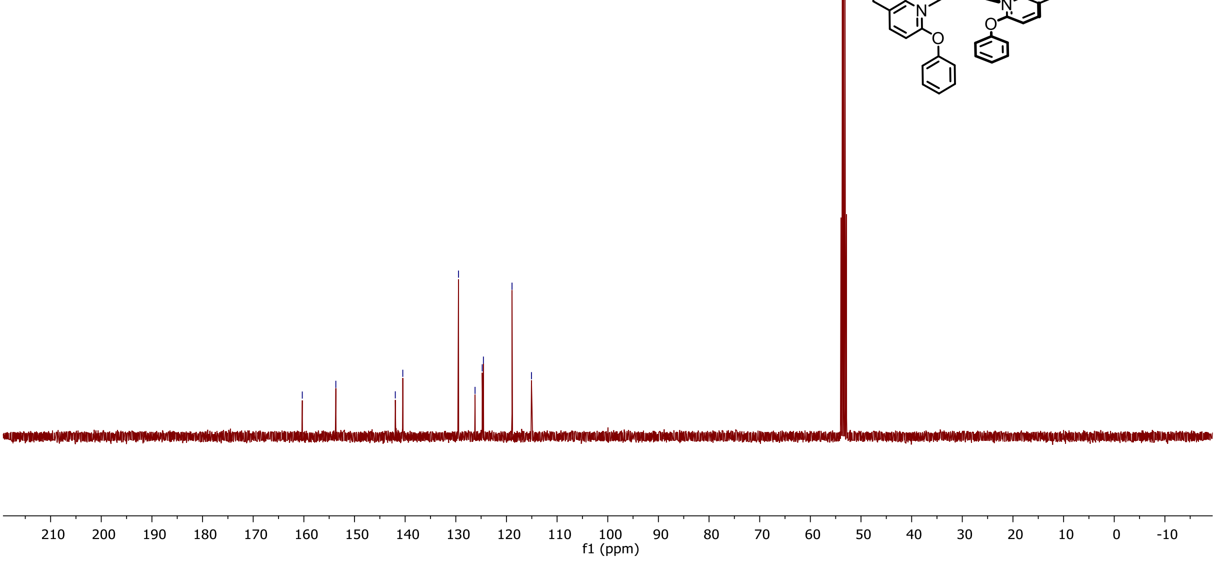

High Resolution Mass Spectrometry Report

Sample Name	**Thomas Brandl / BRT621**	Instrument	maXis 4G
Comment	10 ug/mL in DCM, analyzed in MeOH	Method	24 Direct_pos_high.m

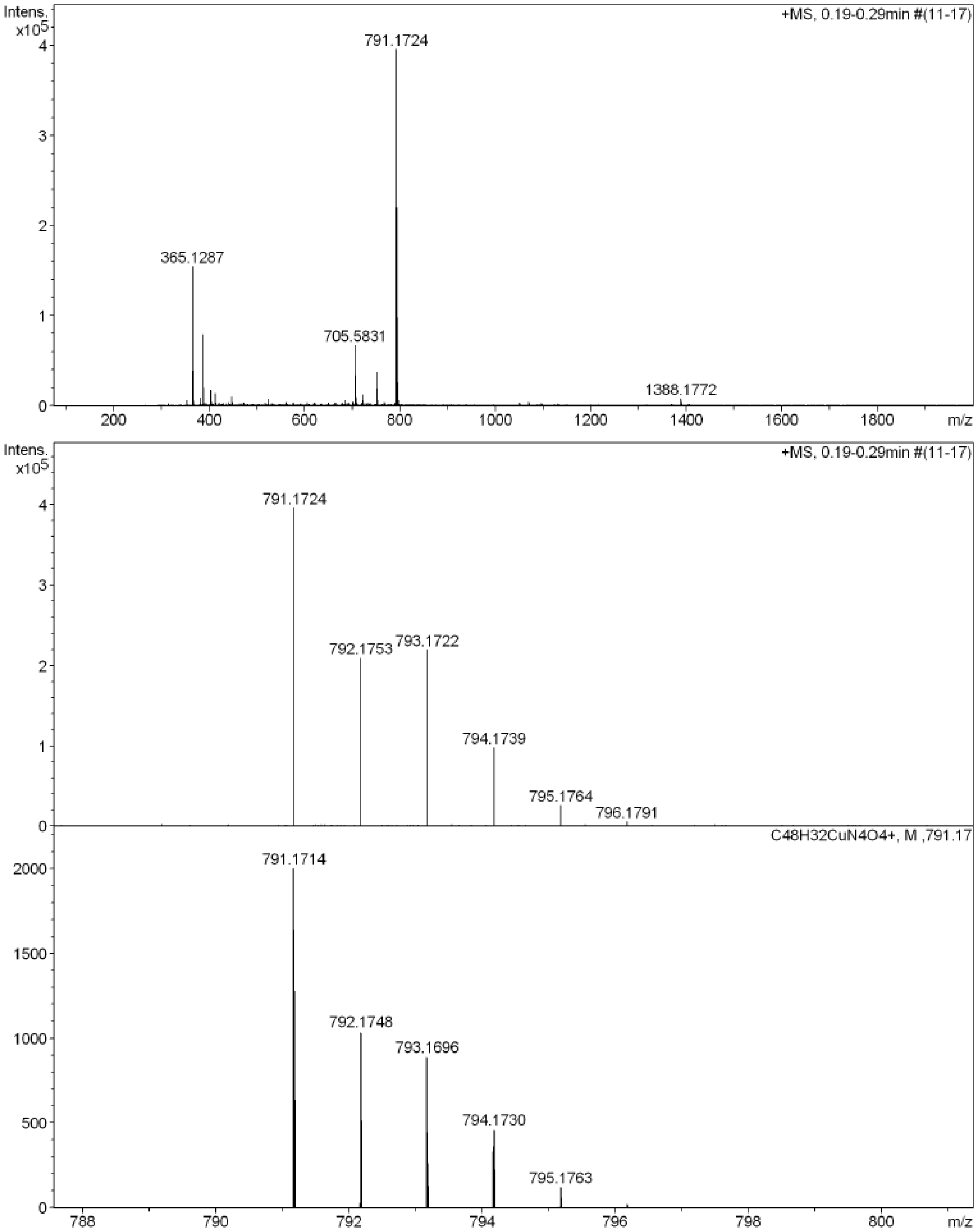

High Resolution Mass Spectrometry Report

Measured m/z vs. theoretical m/z

Meas. m/z	#	Formula	Score	m/z	err [mDa]	err [ppm]	mSigma	rdb	e⁻ Conf	z
791.1724	1	C 48 H 32 Cu N 4 O 4	100.00	791.1714	-1.0	-1.3	18.7	34.5	even	1+

Mass list

#	m/z	I %	I
1	315.1930	0.5	2049
2	353.2661	1.4	5707
3	365.1287	39.1	154740
4	366.1318	9.8	38636
5	367.1345	1.4	5405
6	381.2977	2.3	8988
7	382.3007	0.6	2232
8	385.2923	0.4	1746
9	387.1105	19.9	78798
10	388.1136	5.0	19678
11	389.1168	0.8	3106
12	393.2980	0.7	2753
13	403.0845	4.3	17186
14	404.0875	1.1	4445
15	407.3131	0.5	1953
16	413.2663	3.4	13633
17	414.2695	0.9	3365
18	421.3294	0.8	3086
19	429.2408	0.5	2057
20	429.3189	0.7	2788
21	435.3444	0.5	2111
22	441.2981	0.9	3541
23	447.3449	2.6	10301
24	448.3480	0.7	2704
25	449.3612	0.9	3716
26	463.3166	0.5	1972
27	463.3759	0.5	2086
28	469.3286	0.7	2612
29	473.3448	0.8	3038
30	487.3611	0.5	1790
31	517.3720	0.8	3123
32	523.3248	1.8	7239
33	524.3277	0.6	2324
34	531.3863	0.6	2247
35	533.3456	0.6	2186
36	561.3970	1.0	3893
37	575.4131	0.6	2479
38	577.3722	0.8	3080
39	577.4805	0.5	1910
40	591.3877	0.5	2028
41	591.4955	0.5	2032
42	605.4225	0.9	3493
43	619.4382	0.6	2532
44	621.3982	0.8	3046
45	635.4136	0.5	1919
46	649.4511	0.8	3201
47	663.4638	0.8	3284
48	665.4242	0.8	3187
49	677.4869	0.5	1826
50	679.4382	0.7	2847
51	685.4359	1.4	5647
52	686.4395	0.7	2738
53	691.5094	0.6	2525
54	693.4728	0.7	2742
55	700.6277	1.0	4012
56	701.6308	0.5	1978
57	705.5267	0.6	2427
58	705.5831	16.9	66977
59	706.5861	8.4	33333
60	707.4889	0.6	2480
61	707.5870	2.4	9530
62	708.5869	0.7	2783

#	m/z	I %	I
63	709.4492	0.5	2039
64	719.5428	0.7	2675
65	721.5014	0.5	2021
66	721.5609	3.1	12192
67	722.5650	1.5	5785
68	723.4669	0.4	1758
69	723.5628	0.7	2797
70	729.2487	0.7	2799
71	733.5567	0.6	2417
72	737.4995	0.6	2429
73	747.5721	0.4	1771
74	751.2312	9.3	36914
75	751.5163	0.5	1919
76	752.2346	4.8	18792
77	753.2374	1.4	5601
78	767.2048	0.9	3697
79	768.2073	0.5	2160
80	789.1914	0.7	2622
81	790.1943	0.5	2028
82	791.1724	100.0	395546
83	791.5837	0.5	1809
84	792.1753	53.1	210003
85	793.1722	55.5	219700
86	794.1739	24.8	97946
87	795.1764	6.6	26029
88	795.5418	0.5	2004
89	796.1791	1.4	5482
90	1049.5577	0.8	3333
91	1050.5595	0.7	2703
92	1069.7029	1.0	3938
93	1070.7061	0.8	3092
94	1095.8592	0.7	2586
95	1096.8612	0.4	1772
96	1129.9375	0.5	2171
97	1388.1772	2.0	7907
98	1389.1815	1.8	6925
99	1390.1827	1.0	3957
100	1391.1852	0.5	1965

Acquisition Parameter

**General**	Fore Vacuum<br>Scan Begin	2.69e+000 mBar<br>75 m/z	High Vacuum<br>Scan End	1.02e-007 mBar<br>2000 m/z	Source Type<br>Ion Polarity	ESI<br>Positive
**Source**	Set Nebulizer<br>Set Dry Heater	0.4 Bar<br>180 °C	Set Capillary<br>Set End Plate Offset	3600 V<br>-500 V	Set Dry Gas	4.0 l/min
**Quadrupole**	Set Ion Energy (MS only)	4.0 eV				
**Coll. Cell**	Collision Energy	10.0 eV	Set Collision Cell RF	1000.0 Vpp		
**Ion Cooler**	Set Ion Cooler Transfer Time	160.0 µs	Set Ion Cooler Pre Pulse Storage Time	18.0 µs		

^{1}H-, ^{13}C-, COSY-, NOESY-, HMQC-, HMBC-NMR (CD_2Cl_2, 500/126 MHz, 22 °C) and HR-ESI-MS spectra and full assignment of compound (1)

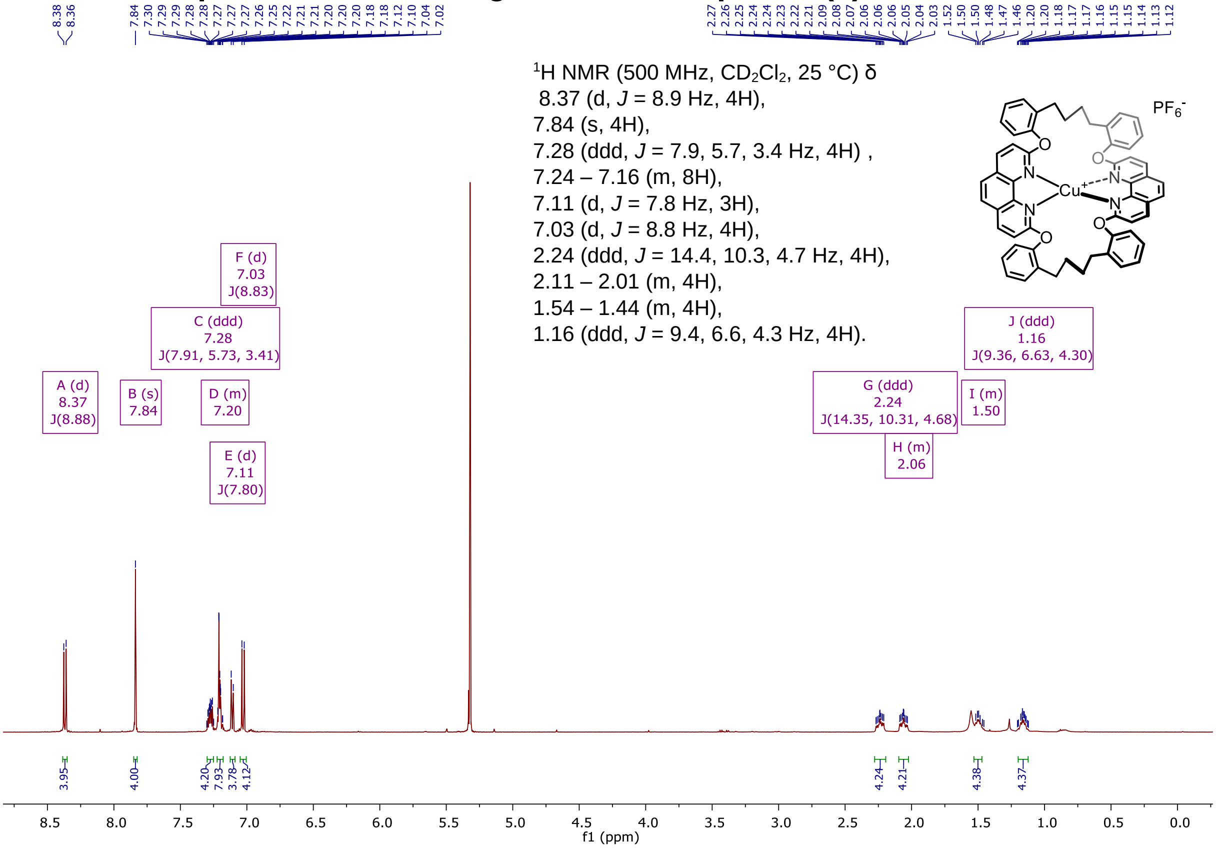

^{1}H NMR (500 MHz, CD_2Cl_2, 25 °C) δ 8.37 (d, *J* = 8.9 Hz, 4H), 7.84 (s, 4H), 7.28 (ddd, *J* = 7.9, 5.7, 3.4 Hz, 4H) , 7.24 – 7.16 (m, 8H), 7.11 (d, *J* = 7.8 Hz, 3H), 7.03 (d, *J* = 8.8 Hz, 4H), 2.24 (ddd, *J* = 14.4, 10.3, 4.7 Hz, 4H), 2.11 – 2.01 (m, 4H), 1.54 – 1.44 (m, 4H), 1.16 (ddd, *J* = 9.4, 6.6, 4.3 Hz, 4H).

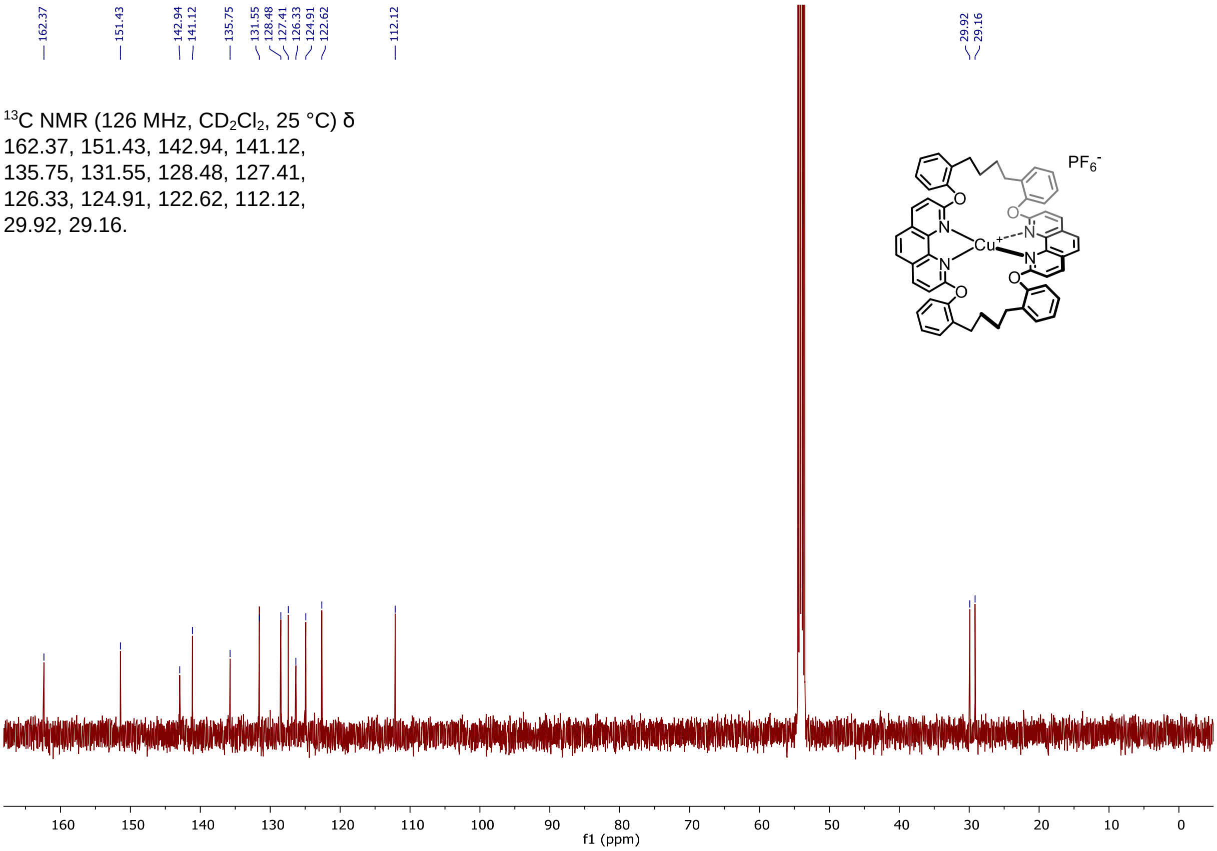

^{13}C NMR (126 MHz, CD_2Cl_2, 25 °C) δ 162.37, 151.43, 142.94, 141.12, 135.75, 131.55, 128.48, 127.41, 126.33, 124.91, 122.62, 112.12, 29.92, 29.16.

COSY

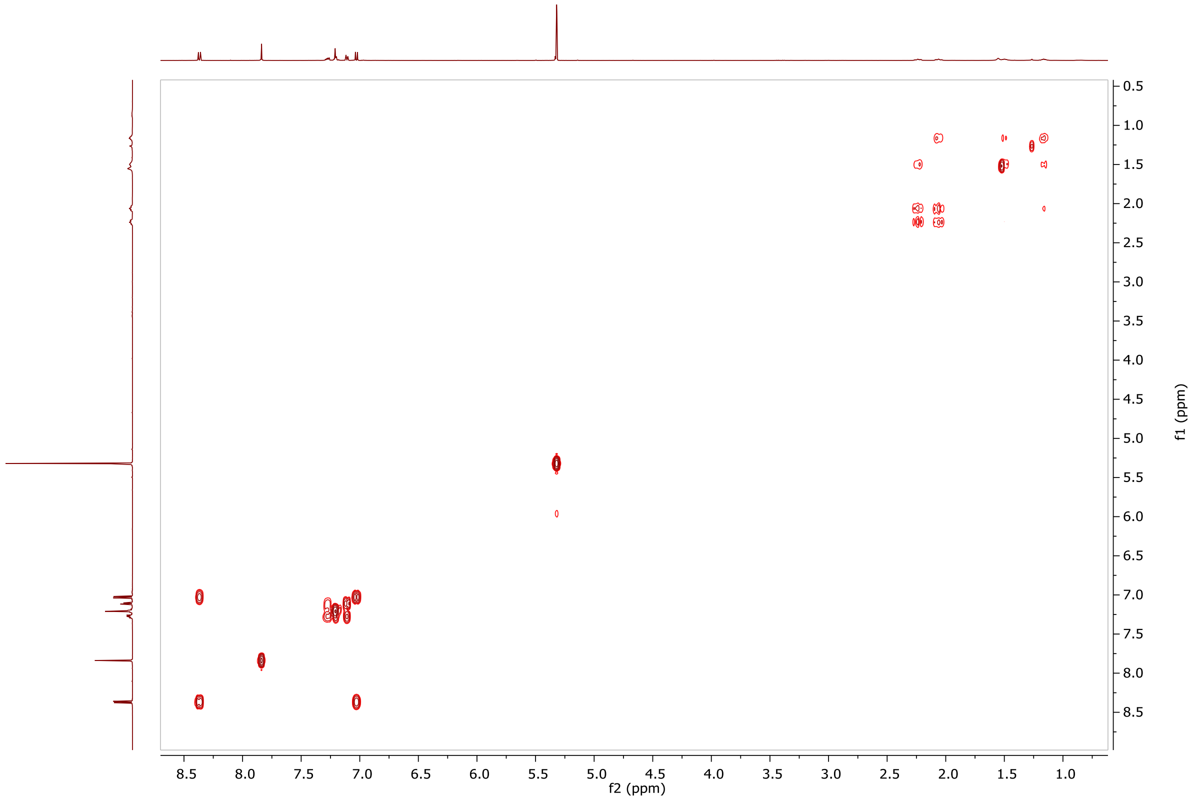

NOESY

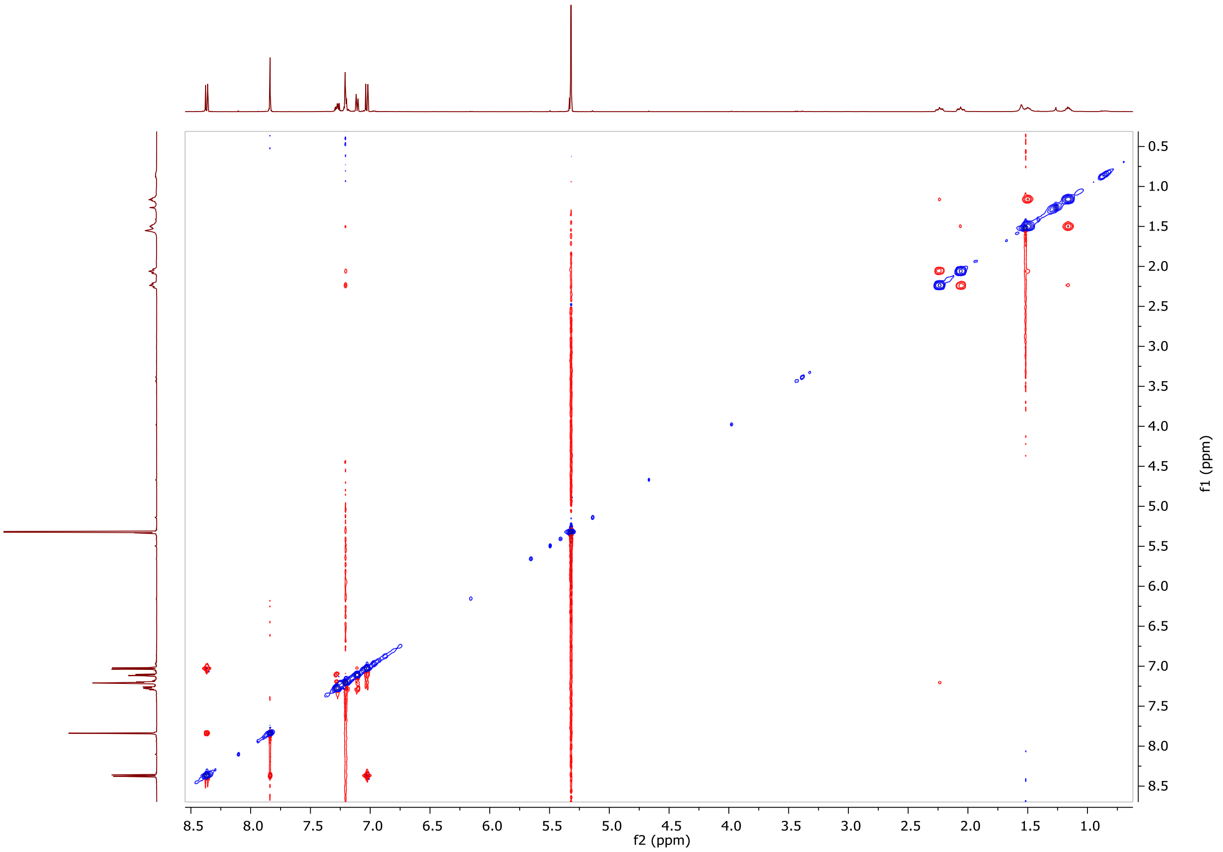

HMQC

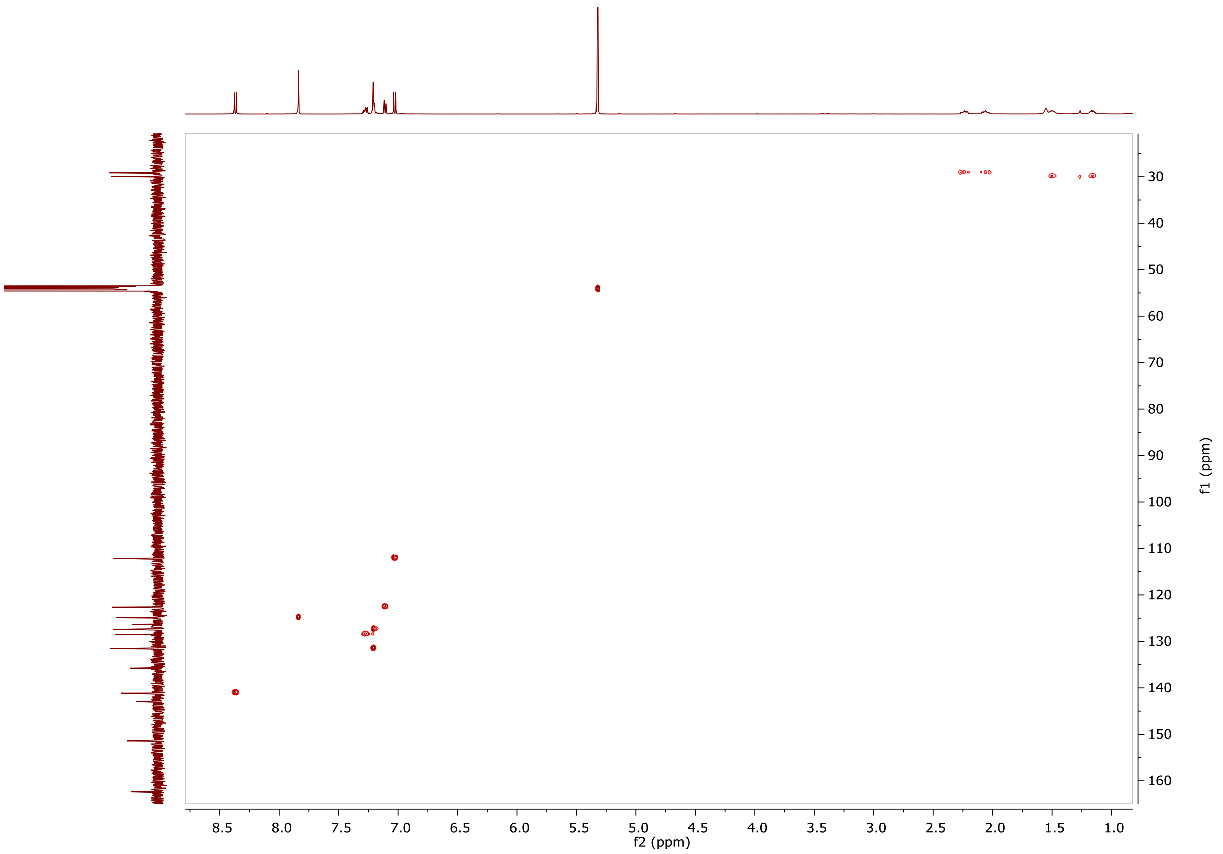

HMBC

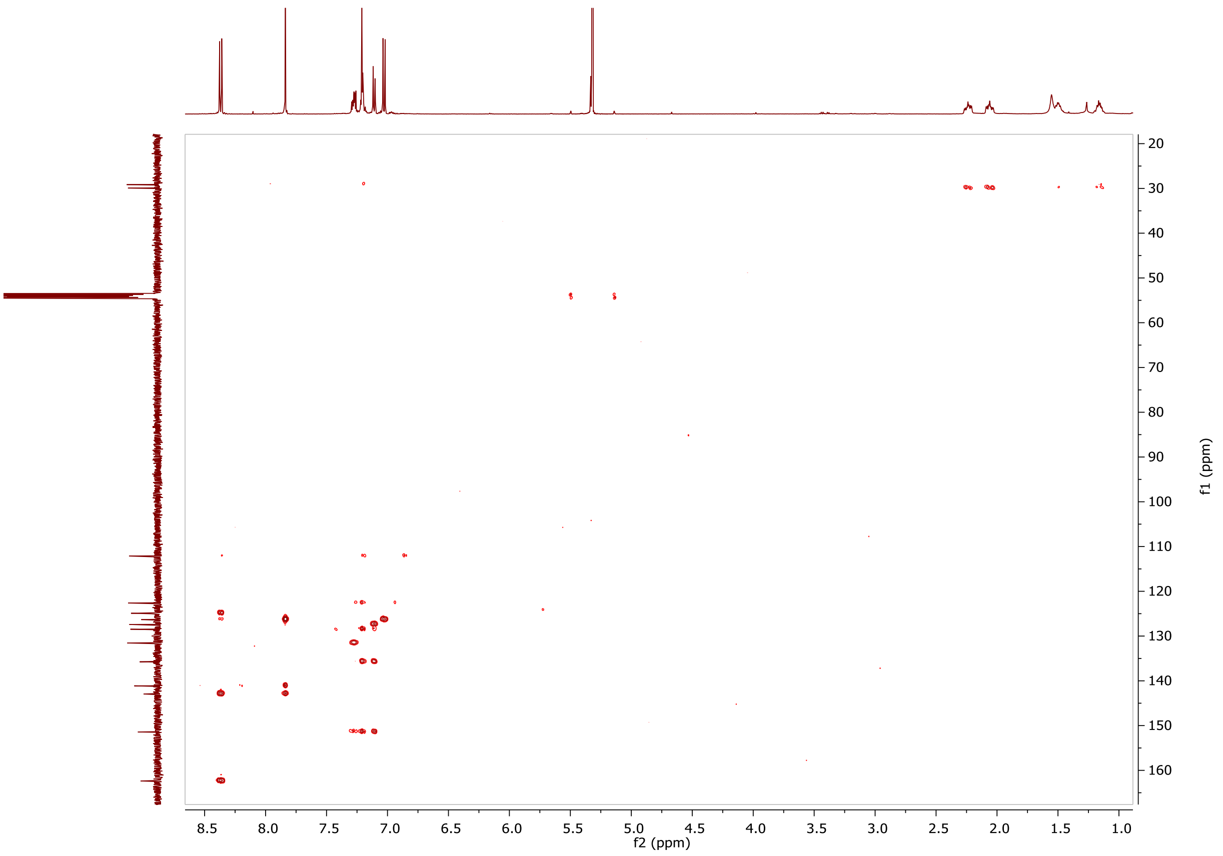

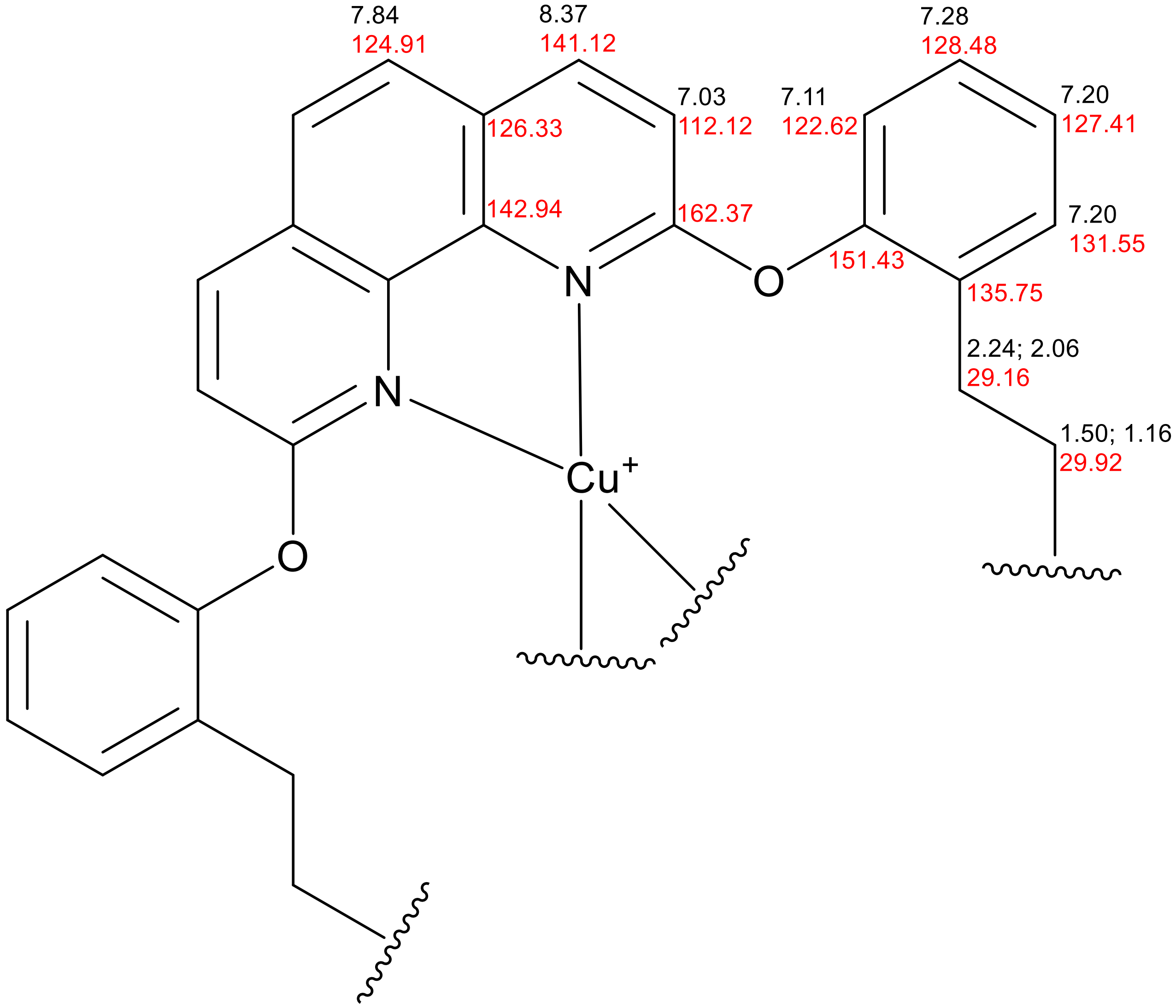

7.84
124.91
8.37
141.12
7.28
128.48
7.03
112.12
7.11
122.62
7.20
127.41
126.33
142.94
162.37
7.20
131.55
151.43
N
O
135.75
2.24; 2.06
29.16
N
1.50; 1.16
29.92
Cu+
O

High Resolution Mass Spectrometry Report

Sample Name	**Thomas Brandl / BRT610**	Instrument	maXis 4G
Comment	10 ug/mL in DCM, analyzed in MeOH	Method	23 Direct_pos_higher.m

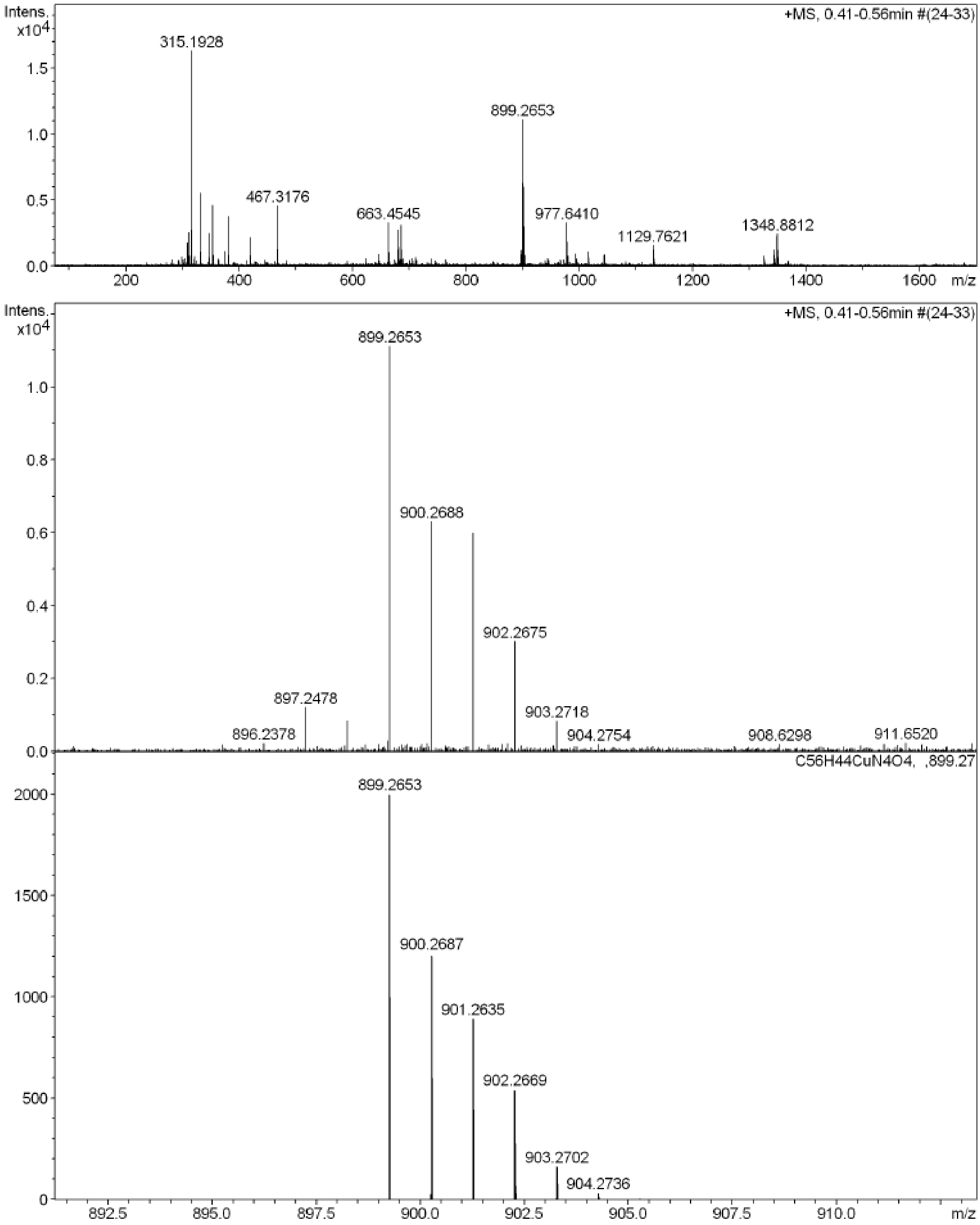

Bruker Compass DataAnalysis 4.0 | Acquisition Date 26.04.2019 09:52:39 | Page 1 of 3

High Resolution Mass Spectrometry Report

Measured m/z vs. theoretical m/z

Meas. m/z	#	Formula	Score	m/z	err [mDa]	err [ppm]	mSigma	rdb	e⁻ Conf	z
899.2653	1	C 56 H 44 Cu N 4 O 4	100.00	899.2653	-0.0	-0.0	54.7	36.5	even	1+

Mass list

#	m/z	I %	I
1	282.2775	3.0	489
2	293.2442	2.5	416
3	299.1614	4.1	662
4	301.1387	3.0	484
5	304.2602	3.8	623
6	309.2061	10.6	1735
7	310.2375	15.6	2540
8	312.1847	4.6	750
9	315.1928	100.0	16305
10	316.1958	16.9	2762
11	320.1810	3.2	520
12	321.2759	4.4	722
13	325.2002	2.4	390
14	331.1660	5.7	929
15	331.1876	33.8	5518
16	331.2162	2.3	371
17	332.1910	6.6	1074
18	347.1613	3.2	520
19	347.1833	15.1	2455
20	348.1860	3.9	631
21	353.2664	28.3	4607
22	354.2689	5.0	813
23	363.2152	3.6	587
24	364.2106	2.8	464
25	375.2145	6.8	1103
26	381.2978	23.2	3785
27	382.2997	5.0	822
28	413.2669	2.7	439
29	419.2416	13.4	2182
30	420.2445	4.9	795
31	427.2851	2.2	362
32	445.3329	2.9	471
33	467.3176	27.9	4552
34	468.3190	7.9	1293
35	483.3108	2.6	428
36	591.3660	2.3	382
37	623.3879	3.6	595
38	645.4000	5.5	896
39	645.4725	4.9	799
40	646.4730	2.4	391
41	647.4553	2.3	378
42	663.4545	20.2	3286
43	664.4563	6.3	1029
44	673.5031	3.0	497
45	674.4981	2.5	404
46	679.4187	7.8	1264
47	680.4215	3.3	541
48	680.4807	16.8	2740
49	681.4847	8.7	1420
50	682.4866	2.3	382
51	683.5414	3.3	546
52	685.4343	19.0	3104
53	686.4385	7.8	1266
54	687.4390	3.0	487
55	689.4990	3.4	557
56	701.4063	2.8	462
57	705.5811	3.7	608
58	706.5868	2.5	400
59	711.5730	4.0	649
60	712.5755	2.7	444
61	739.6013	3.5	564
62	745.5031	2.4	396

#	m/z	I %	I
63	764.5721	3.1	506
64	847.5583	2.2	362
65	897.2478	7.3	1196
66	898.2510	5.2	841
67	899.2653	68.1	11106
68	900.2688	38.7	6307
69	901.2660	36.7	5984
70	902.2675	18.5	3014
71	903.2718	5.1	830
72	939.5988	2.6	422
73	944.7277	3.7	600
74	945.7299	2.6	417
75	966.7093	3.2	517
76	967.7112	2.4	386
77	972.6818	3.0	488
78	977.6410	20.2	3290
79	978.6423	11.1	1803
80	979.6429	4.5	738
81	993.6344	5.8	941
82	994.6399	3.5	564
83	1015.7156	6.5	1062
84	1016.7143	4.0	657
85	1043.7433	5.1	825
86	1044.7455	5.3	857
87	1129.7621	9.7	1574
88	1130.7632	7.7	1250
89	1131.7732	3.4	562
90	1325.9013	4.6	753
91	1326.8993	3.3	543
92	1342.9248	7.6	1241
93	1343.9284	5.1	838
94	1344.9284	3.6	586
95	1347.8848	13.9	2274
96	1347.9418	2.4	392
97	1348.8812	14.8	2420
98	1349.8919	7.4	1200
99	1350.8915	3.9	634
100	1368.0272	2.3	379

Acquisition Parameter

**General**	Fore Vacuum	2.68e+000 mBar	High Vacuum	1.03e-007 mBar	Source Type	ESI
	Scan Begin	75 m/z	Scan End	1700 m/z	Ion Polarity	Positive
**Source**	Set Nebulizer	0.4 Bar	Set Capillary	3600 V	Set Dry Gas	4.0 l/min
	Set Dry Heater	180 °C	Set End Plate Offset	-500 V		
**Quadrupole**	Set Ion Energy (MS only)	4.0 eV				
**Coll. Cell**	Collision Energy	8.0 eV	Set Collision Cell RF	500.0 Vpp		
**Ion Cooler**	Set Ion Cooler Transfer Time	80.0 µs	Set Ion Cooler Pre Pulse Storage Time	18.0 µs		

Computational investigations

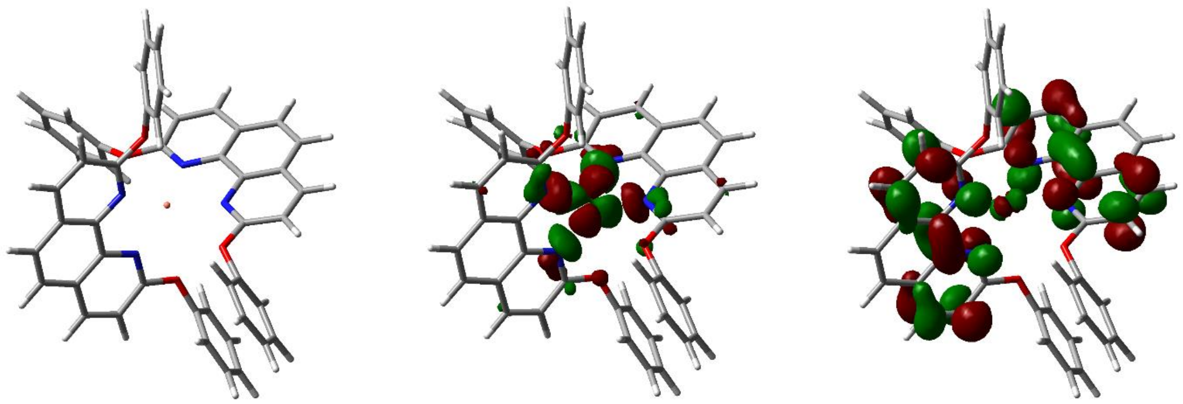

Figure SI1: DFT-optimized structure (left) and calculated frontier orbitals HOMO (middle) and LUMO (right) of reference complex **15**.

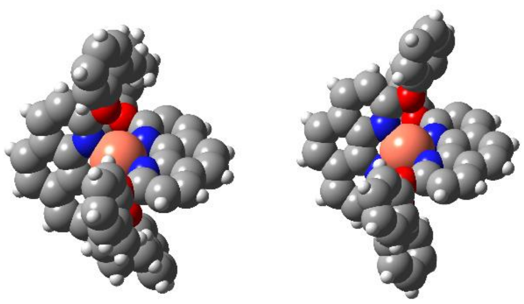

Figure SI2: Space-filling representations of the optimized excited state geometries of the macrocyclic complex **1** (left) and the reference complex **15** (right).

Crystal data for 11

Single crystals were grown by vapor diffusion technique using dichloromethane as solvent and diethyl ether as anti-solvent. Solid state structure in the manuscript are displayed with rotation ellipsoids at 50% probability. Hydrogen atoms, solvent moleclues and the PF_6^- counter ions were omitted for clarity. Color code: N: blue, Cu: yellow, C: gray for one and purple for the other ligand for clarity.

Formula $C_{56}H_{32}Cu_1F_6N_4P_1$, M = 969.40, F(000) = 988, brown block, size 0.23 x 0.28 x 0.31 mm^3, triclinic, space group P -1 , Z = 2, a = 8.8141(10) Å, b = 14.6956(16) Å, c = 16.8143(19) Å, α = 95.110(3)°, β = 95.863(3)°, γ = 90.880(3)°, V = 2157.3(4) Å^3, $D_{calc.}$ = 1.492 Mg*m^{-3}. The crystal was measured on a Bruker Kappa Apex2 diffractometer at 130(2)K using graphite-monochromated Cu K_α-radiation with λ = 1.54178 Å, Θ_{max} = 70.376°. Minimal/maximal transmission 0.91/1.00, μ = 1.677 mm^{-1}. The Apex2 suite has been used for datacollection and integration. From a total of 21167 reflections, 7807 were independent (merging r = 0.041). From these, 7806 were considered as observed (I>2.0σ(I)) and were used to refine 613 parameters. The structure was solved by other methods using the program Superflip. Least-squares refinement against Fsqd was carried out on all non-hydrogen atoms using the program CRYSTALS. R = 0.0371 (observed data), wR = 0.0910 (all data), GOF = 0.9995. Minimal/maximal residual electron density = -0.28/0.38 e Å^{-3}. Chebychev polynomial weights were used to complete the refinement. Plots were produced using CAMERON. Crystallographic data (excluding structure factors) for the structure in this paper have been deposited with the Cambridge Crystallographic Data Center, the deposition number is (1948428). Copies of the data can be obtained, free of charge, on application to the CCDC, 12 Union Road, Cambridge CB2 1EZ, UK [fax: +44-1223-336033 or e-mail: deposit@ccdc.cam.ac.uk].

Photostability investigations

A very similar strategy as in our recent investigation on acridinium dyes was used to compare the photostabilities of **1** and **15**.[1]

We irradiated diluted and deoxygenated solutions of both complexes, and monitored their UV-Vis spectra over two hours of photoirradiation. The cuvettes were irradiated in the sample chamber of the spectrophotometer (see Figure SI4) with a 455 nm LED from Thorlabs (M455L3-C1, 500 mW optical output). Our cuvette holder permits LED irradiation of the whole detection volume. For recording the absorption spectra after the desired irradiation times, the LED was blocked for 2 minutes.

The absorptions of both irradiated solutions at the LED peak wavelength (455 nm) were standardized to 0.10, which ensures that almost the same amount of light is absorbed while sample heating is avoided. Concentrations of ~36 μM for **1** and ~211 μM for **15** were required for these standardized conditions.

The UV-Vis data displayed in figure 6 of the main paper show good photostability for **1** under our test conditions. A significant decrease of the MLCT absorption band is expected to occur upon photodecomposition, but all spectra are virtually identical; the maximum relative absorption variation is with less than 0.5% within the accuracy of the analysis.

The situation is completely different for **15**. Our photostability assay revealed a constant decrease of the MLCT band, which amounts to 7% after 2 hours of photoirradiation (compare, lower part of figure 6a and figure 6b). Assuming that the decomposition products do not absorb at the detection wavelength allows us to set a lower limit for the concentration change caused by photodecomposition: **14.8 μM** (211 μM x 0.07). To be able to make a relative stability statement, we assume a maximum absolute concentration change for **1** under identical irradiation conditions of **0.4 μM**, corresponding to an experimental error of up to 1% hiding the alteration of the observed MLCT band (36 μM x 0.01). Taking the widely differing starting concentration of both complexes into account[2] the actual photodecomposition of **15** is thus significantly faster (compared to that of **1**) than the absorption spectra in figure 6 suggest. Hence, the analysis presented in this section revealed the photodegradation of **1** (macrocycle) to be slower than that of **15** by a factor of at least ~**37**.

In order to identify the photodecomposition product, we irradiated complex **15** in an NMR tube. The peaks in acetonitrile-d_3 are rather broad, which is the reason why we performed this experiment in deuterated dichloromethane. In the NMR spectrum recorded after irradiation with a blue high-power LED at 440 nm (from Kessil), which was very recently purchased and has a much higher output than the 455 nm LED used for the investigations presented in figure 3 of the main paper, we observed noticeable photodecomposition after 3h of irradiation, but we could not detect the release of the free ligand (Figure SI3). The photodecomposition products could not be identified.

---

[1] C. Fischer, C. Kerzig, B. Zilate, O. S. Wenger, C. Sparr, ACS Catal. 2019, DOI 10.1021/acscatal.9b03606.

[2] C. Kerzig, X. Guo, O. S. Wenger, *J. Am. Chem. Soc.*, **2019**, 141, 2122-2127.

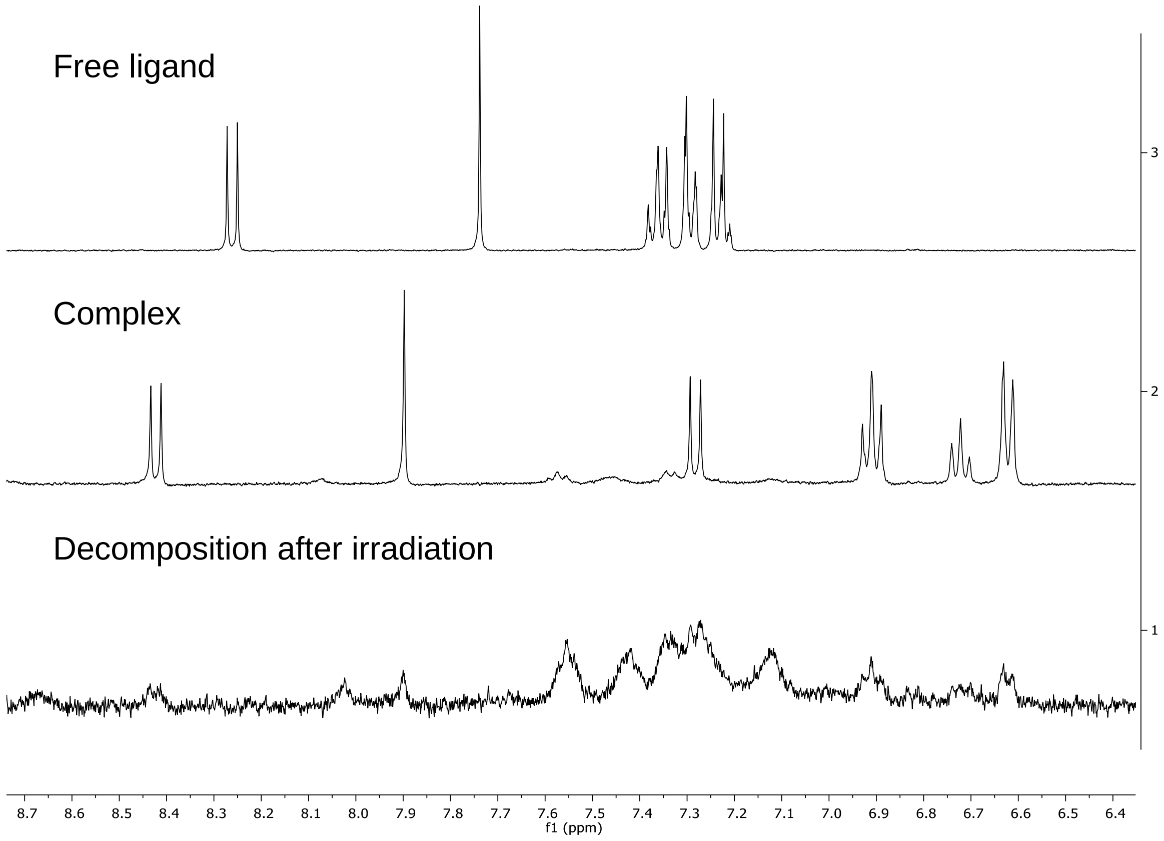

Figure SI3: NMR investigations of the photodecomposition of the reference complex **15** upon irradiation with an LED at 440 nm. The NMR spectra in deuterated dichloromethane of the free ligand (top), the complex (middle) and the decomposition product after irradiation for 3 hours (bottom) are illustrated. It can be seen that the decomposition product is not the free ligand.

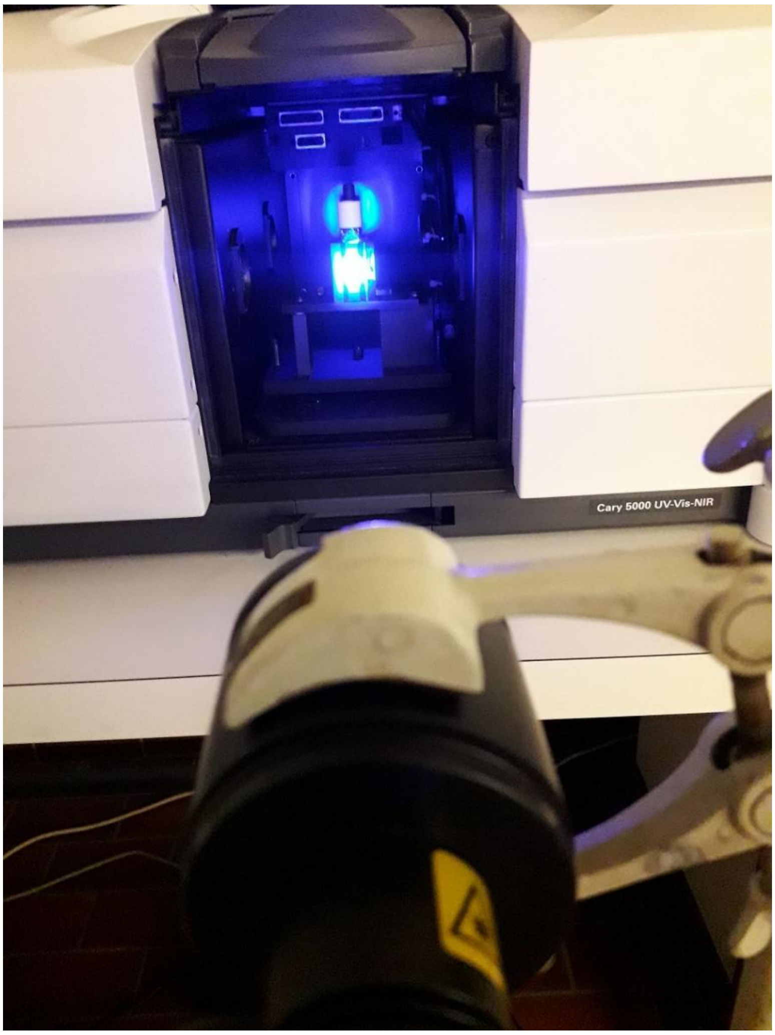

Figure SI4: Instrumental setup for the photostability investigations.

Table SI1: UV-Vis data of complex **1** after 0, 60 and 120 minutes photoirradiation.

Wavelength [nm]	Absorption before irradiation	Absorption after 60 min irradiation	Absorption after 120 min irradiation
750	6.33E-04	5.59E-04	6.62E-04
749	6.76E-04	5.29E-04	6.31E-04
748	7.24E-04	6.53E-04	7.36E-04
747	5.84E-04	5.49E-04	6.76E-04
746	6.35E-04	6.58E-04	7.66E-04
745	7.26E-04	7.03E-04	6.47E-04
744	5.78E-04	6.04E-04	6.13E-04
743	6.72E-04	6.15E-04	7.78E-04
742	6.47E-04	5.66E-04	7.55E-04
741	6.68E-04	5.83E-04	6.42E-04
740	6.54E-04	6.20E-04	6.64E-04
739	6.58E-04	6.30E-04	6.84E-04
738	7.14E-04	7.67E-04	7.11E-04
737	5.85E-04	7.25E-04	6.35E-04
736	6.58E-04	6.54E-04	6.13E-04
735	5.59E-04	5.11E-04	5.92E-04

734	6.16E-04	6.23E-04	6.62E-04
733	6.20E-04	6.77E-04	6.65E-04
732	5.75E-04	6.27E-04	6.60E-04
731	6.79E-04	6.74E-04	6.88E-04
730	6.21E-04	4.94E-04	5.71E-04
729	5.85E-04	7.07E-04	7.45E-04
728	5.72E-04	5.99E-04	6.07E-04
727	7.09E-04	6.27E-04	6.59E-04
726	5.00E-04	5.13E-04	5.03E-04
725	5.82E-04	5.72E-04	5.82E-04
724	5.93E-04	5.45E-04	4.75E-04
723	6.36E-04	5.57E-04	5.31E-04
722	6.61E-04	5.26E-04	5.59E-04
721	6.27E-04	5.44E-04	4.87E-04
720	5.71E-04	4.99E-04	4.84E-04
719	4.90E-04	5.00E-04	4.97E-04
718	5.25E-04	5.28E-04	5.37E-04
717	5.53E-04	5.02E-04	4.14E-04
716	4.85E-04	4.86E-04	5.26E-04
715	5.62E-04	5.41E-04	4.40E-04
714	5.30E-04	5.43E-04	4.96E-04
713	5.70E-04	4.52E-04	4.62E-04
712	4.49E-04	4.78E-04	4.05E-04
711	5.06E-04	3.82E-04	4.28E-04
710	4.61E-04	4.62E-04	4.56E-04
709	4.46E-04	3.90E-04	3.67E-04
708	5.10E-04	4.04E-04	3.89E-04
707	4.37E-04	4.50E-04	3.47E-04
706	4.35E-04	4.40E-04	3.42E-04
705	4.12E-04	3.92E-04	3.50E-04
704	4.70E-04	4.08E-04	3.78E-04
703	4.24E-04	3.42E-04	4.04E-04
702	4.98E-04	3.99E-04	3.85E-04
701	4.35E-04	4.06E-04	3.89E-04
700	3.83E-04	3.67E-04	2.96E-04
699	4.24E-04	4.11E-04	3.34E-04
698	4.03E-04	4.28E-04	4.14E-04
697	4.31E-04	3.85E-04	3.86E-04
696	4.35E-04	3.79E-04	3.14E-04
695	3.22E-04	3.25E-04	3.14E-04
694	3.78E-04	3.81E-04	3.02E-04
693	3.71E-04	3.53E-04	3.03E-04
692	4.12E-04	3.88E-04	3.37E-04
691	3.78E-04	3.49E-04	3.32E-04
690	4.33E-04	3.58E-04	3.90E-04
689	3.91E-04	3.64E-04	3.64E-04
688	3.27E-04	3.74E-04	3.08E-04

687	3.62E-04	3.11E-04	2.97E-04
686	4.00E-04	3.42E-04	3.43E-04
685	3.78E-04	3.11E-04	2.89E-04
684	3.96E-04	3.10E-04	2.89E-04
683	3.40E-04	3.45E-04	3.09E-04
682	3.55E-04	2.87E-04	3.23E-04
681	3.54E-04	3.30E-04	3.06E-04
680	3.18E-04	2.88E-04	2.42E-04
679	3.85E-04	3.47E-04	2.81E-04
678	4.30E-04	3.26E-04	2.74E-04
677	4.16E-04	2.76E-04	2.69E-04
676	3.68E-04	3.22E-04	2.63E-04
675	2.79E-04	2.84E-04	2.51E-04
674	3.45E-04	3.22E-04	2.59E-04
673	3.84E-04	3.50E-04	3.25E-04
672	3.81E-04	3.24E-04	3.10E-04
671	3.84E-04	3.24E-04	3.21E-04
670	3.28E-04	3.11E-04	2.60E-04
669	3.73E-04	3.64E-04	2.78E-04
668	3.48E-04	3.35E-04	2.73E-04
667	3.87E-04	3.44E-04	2.95E-04
666	3.19E-04	2.96E-04	3.39E-04
665	3.48E-04	3.42E-04	2.64E-04
664	3.57E-04	3.42E-04	3.34E-04
663	3.25E-04	2.79E-04	2.71E-04
662	4.09E-04	3.56E-04	3.38E-04
661	4.85E-04	4.87E-04	4.75E-04
660	3.23E-04	3.63E-04	2.90E-04
659	3.23E-04	2.45E-04	2.10E-04
658	4.07E-04	4.08E-04	3.51E-04
657	4.85E-04	4.37E-04	3.81E-04
656	3.51E-04	3.00E-04	2.53E-04
655	2.99E-04	2.76E-04	2.86E-04
654	3.95E-04	3.67E-04	3.32E-04
653	4.16E-04	3.94E-04	3.49E-04
652	3.84E-04	3.32E-04	3.11E-04
651	4.17E-04	3.95E-04	3.71E-04
650	3.92E-04	3.86E-04	3.64E-04
649	4.28E-04	3.86E-04	3.60E-04
648	4.42E-04	3.57E-04	3.54E-04
647	4.29E-04	3.82E-04	3.53E-04
646	4.43E-04	3.75E-04	3.82E-04
645	4.38E-04	3.58E-04	3.43E-04
644	4.70E-04	4.55E-04	3.95E-04
643	4.81E-04	4.40E-04	4.24E-04
642	4.72E-04	4.47E-04	4.13E-04
641	5.33E-04	4.85E-04	4.56E-04

640	5.23E-04	4.66E-04	4.76E-04
639	5.35E-04	4.80E-04	4.79E-04
638	5.45E-04	5.10E-04	4.59E-04
637	6.02E-04	5.40E-04	5.24E-04
636	5.97E-04	5.29E-04	5.28E-04
635	6.67E-04	5.84E-04	6.26E-04
634	6.37E-04	5.89E-04	5.98E-04
633	6.50E-04	6.13E-04	5.73E-04
632	6.92E-04	6.81E-04	6.49E-04
631	6.87E-04	6.49E-04	6.36E-04
630	7.36E-04	7.43E-04	7.14E-04
629	7.87E-04	7.31E-04	7.35E-04
628	8.85E-04	7.98E-04	7.99E-04
627	8.88E-04	8.73E-04	8.22E-04
626	8.81E-04	8.24E-04	8.17E-04
625	9.12E-04	8.83E-04	8.60E-04
624	9.66E-04	9.25E-04	8.84E-04
623	0.00103	0.00101	9.63E-04
622	0.00106	0.00103	0.00105
621	0.00111	0.00107	0.00106
620	0.00115	0.00111	0.00108
619	0.00123	0.00119	0.00119
618	0.00131	0.00125	0.00122
617	0.00142	0.00133	0.00129
616	0.00157	0.0014	0.00143
615	0.00159	0.00147	0.00148
614	0.0016	0.00149	0.0015
613	0.00163	0.00155	0.00149
612	0.00175	0.00167	0.00169
611	0.0019	0.00179	0.00176
610	0.00197	0.00187	0.00188
609	0.0021	0.00191	0.00188
608	0.0021	0.00204	0.00201
607	0.00216	0.00214	0.00212
606	0.00227	0.00224	0.00221
605	0.00236	0.00232	0.00231
604	0.00249	0.00246	0.00245
603	0.00263	0.00253	0.00258
602	0.00274	0.00265	0.00267
601	0.00282	0.00283	0.00277
600	0.00294	0.00289	0.00287
599	0.0031	0.00299	0.00305
598	0.00326	0.00322	0.00323
597	0.0034	0.00337	0.00337
596	0.00349	0.00342	0.00344
595	0.00366	0.00359	0.00364
594	0.00382	0.00376	0.0038

593	0.00399	0.00397	0.00396
592	0.00414	0.00408	0.00412
591	0.00432	0.00424	0.00427
590	0.00449	0.00446	0.00447
589	0.00465	0.00462	0.00466
588	0.00486	0.00482	0.00481
587	0.00506	0.00501	0.00503
586	0.00528	0.00519	0.0052
585	0.00543	0.00535	0.0054
584	0.00567	0.00558	0.00565
583	0.00592	0.00582	0.00586
582	0.00604	0.00604	0.00605
581	0.00627	0.00628	0.00622
580	0.00656	0.00651	0.00651
579	0.0068	0.00674	0.00678
578	0.00701	0.00695	0.00706
577	0.00729	0.0072	0.00728
576	0.00756	0.0075	0.00756
575	0.00778	0.00775	0.00777
574	0.00811	0.00803	0.00813
573	0.00835	0.00829	0.00837
572	0.00867	0.00853	0.00863
571	0.00886	0.00878	0.00886
570	0.0091	0.00907	0.00914
569	0.00943	0.00939	0.00945
568	0.00975	0.00969	0.00977
567	0.01008	0.01002	0.01008
566	0.01042	0.01028	0.01037
565	0.01063	0.01062	0.01072
564	0.01101	0.01094	0.011
563	0.01129	0.01121	0.01134
562	0.01168	0.01164	0.01175
561	0.01201	0.01193	0.01202
560	0.01233	0.01227	0.01234
559	0.01267	0.01258	0.01271
558	0.01298	0.01286	0.01302
557	0.01337	0.01331	0.01342
556	0.01364	0.01361	0.01373
555	0.01405	0.01397	0.01408
554	0.0144	0.01432	0.01441
553	0.01479	0.0147	0.01485
552	0.01512	0.01498	0.01519
551	0.01544	0.01537	0.01552
550	0.01583	0.01579	0.01595
549	0.01616	0.01606	0.01624
548	0.01651	0.01646	0.01662
547	0.01693	0.01683	0.01697

546	0.01727	0.01712	0.0173
545	0.01772	0.01761	0.01781
544	0.01802	0.01795	0.01814
543	0.01838	0.01829	0.0184
542	0.01879	0.01876	0.0189
541	0.01918	0.01912	0.01933
540	0.01952	0.01941	0.01959
539	0.01987	0.0198	0.02002
538	0.02026	0.02023	0.02042
537	0.0207	0.02062	0.02079
536	0.02107	0.02099	0.02117
535	0.02143	0.02133	0.0215
534	0.02186	0.02172	0.02195
533	0.02223	0.0221	0.02234
532	0.02255	0.02253	0.02275
531	0.02299	0.02289	0.02316
530	0.02342	0.02328	0.02352
529	0.02376	0.02369	0.02395
528	0.02425	0.02412	0.02437
527	0.02469	0.02448	0.02481
526	0.02504	0.02496	0.02518
525	0.02553	0.02536	0.02562
524	0.02591	0.02586	0.02613
523	0.02637	0.0263	0.02656
522	0.02684	0.02678	0.02703
521	0.02729	0.0272	0.02747
520	0.0278	0.02769	0.02802
519	0.02831	0.0282	0.02854
518	0.02885	0.02875	0.02905
517	0.02935	0.02936	0.02965
516	0.02999	0.02982	0.03019
515	0.03052	0.03038	0.03074
514	0.03116	0.03105	0.0314
513	0.0318	0.03168	0.03207
512	0.0325	0.03239	0.03275
511	0.03322	0.03311	0.03351
510	0.03404	0.0339	0.03428
509	0.03476	0.03463	0.03505
508	0.03559	0.03549	0.03588
507	0.03647	0.03641	0.03682
506	0.03744	0.03735	0.03774
505	0.03851	0.03838	0.03882
504	0.03953	0.03943	0.03983
503	0.04062	0.04048	0.04097
502	0.04181	0.04169	0.04217
501	0.04301	0.0429	0.04336
500	0.04432	0.04422	0.04474

499	0.04568	0.04555	0.04608
498	0.04715	0.04701	0.0475
497	0.04867	0.04845	0.04906
496	0.05009	0.04991	0.05055
495	0.0517	0.05152	0.05215
494	0.05334	0.0532	0.05384
493	0.05503	0.0549	0.0555
492	0.05675	0.05662	0.05723
491	0.05848	0.05831	0.05894
490	0.06024	0.06007	0.06077
489	0.06201	0.06182	0.06255
488	0.06383	0.06361	0.06438
487	0.06562	0.06547	0.06618
486	0.06738	0.06723	0.06794
485	0.06912	0.069	0.06973
484	0.07088	0.07065	0.07142
483	0.07259	0.07234	0.07319
482	0.07416	0.07395	0.07477
481	0.0758	0.0756	0.07639
480	0.07741	0.07717	0.07801
479	0.07895	0.07865	0.07951
478	0.08029	0.08003	0.08092
477	0.08165	0.08142	0.08232
476	0.08291	0.08266	0.08364
475	0.08419	0.08394	0.08487
474	0.0853	0.08512	0.086
473	0.08642	0.08617	0.08708
472	0.08746	0.08715	0.08808
471	0.08843	0.0881	0.08902
470	0.08931	0.08905	0.08995
469	0.09014	0.08987	0.09083
468	0.09096	0.09068	0.09165
467	0.09167	0.09137	0.09229
466	0.09227	0.09199	0.09299
465	0.09291	0.0926	0.09358
464	0.09346	0.09316	0.09415
463	0.09396	0.09374	0.09469
462	0.09447	0.09422	0.0952
461	0.09493	0.09465	0.09563
460	0.09532	0.09502	0.09595
459	0.09564	0.09533	0.09634
458	0.09596	0.09566	0.09666
457	0.09619	0.09591	0.09695
456	0.09639	0.09618	0.09719
455	0.09665	0.09634	0.09724
454	0.09671	0.09644	0.09736
453	0.09685	0.09655	0.09754

452	0.09687	0.09656	0.09761
451	0.09684	0.09652	0.09755
450	0.09682	0.09653	0.09745
449	0.09669	0.09644	0.09736
448	0.0965	0.0962	0.09724
447	0.0963	0.09591	0.09685
446	0.09594	0.09561	0.09658
445	0.09562	0.09536	0.09637
444	0.0953	0.09503	0.09602
443	0.09481	0.09454	0.09549
442	0.09437	0.09408	0.09499
441	0.09381	0.09351	0.09447
440	0.09327	0.09299	0.09391
439	0.09265	0.09235	0.0933
438	0.09203	0.09172	0.09261
437	0.09136	0.09108	0.09198
436	0.09056	0.09029	0.0912
435	0.08984	0.08954	0.0904
434	0.08903	0.08877	0.08965
433	0.08828	0.08795	0.08887
432	0.08743	0.08718	0.08806
431	0.08661	0.08635	0.08719
430	0.08576	0.08555	0.08635
429	0.08498	0.0847	0.08551
428	0.08414	0.08385	0.08473
427	0.08323	0.08292	0.08381
426	0.08236	0.08213	0.08292
425	0.08153	0.08126	0.08209
424	0.08073	0.08048	0.08126
423	0.07987	0.07958	0.08035
422	0.07896	0.07866	0.07948
421	0.07813	0.07785	0.07867
420	0.07727	0.07701	0.07778
419	0.07642	0.07615	0.07695
418	0.07554	0.07533	0.07599
417	0.07473	0.07449	0.07517
416	0.07379	0.07355	0.0743
415	0.07304	0.07276	0.07351
414	0.07217	0.07195	0.0727
413	0.07145	0.07117	0.0719
412	0.07056	0.07038	0.0711
411	0.06991	0.06966	0.07029
410	0.06912	0.06886	0.0696
409	0.06839	0.06813	0.06885
408	0.06776	0.0675	0.06816
407	0.06712	0.06687	0.0675
406	0.06654	0.06629	0.06693

405	0.06586	0.0657	0.06626
404	0.06533	0.06513	0.06573
403	0.06486	0.06457	0.06516
402	0.06436	0.06408	0.06471
401	0.06394	0.06366	0.0643
400	0.06347	0.06331	0.06387
399	0.06316	0.06291	0.06348
398	0.06281	0.06259	0.0632
397	0.06242	0.0622	0.06276
396	0.06221	0.06195	0.06261
395	0.06204	0.06177	0.06237
394	0.06181	0.06161	0.06216
393	0.06174	0.0615	0.06206
392	0.06156	0.06132	0.06192
391	0.06151	0.0612	0.06175
390	0.06152	0.06125	0.06182
389	0.06152	0.06128	0.06187
388	0.06158	0.0614	0.06194
387	0.0619	0.06159	0.06214
386	0.06196	0.06166	0.06223
385	0.0622	0.06189	0.06238
384	0.06247	0.06216	0.06271
383	0.06291	0.06263	0.06318
382	0.06331	0.06308	0.06358
381	0.0638	0.06359	0.06413
380	0.06441	0.06415	0.06458
379	0.06498	0.06467	0.06512
378	0.06564	0.06529	0.06586
377	0.06641	0.06611	0.06666
376	0.06724	0.06687	0.06744
375	0.06813	0.06785	0.06829
374	0.06911	0.06866	0.06911
373	0.07002	0.06948	0.06997
372	0.07102	0.07042	0.07093
371	0.07195	0.07148	0.07195
370	0.07302	0.07257	0.07298
369	0.07396	0.07346	0.07383
368	0.07498	0.07428	0.07474
367	0.07586	0.07524	0.07555
366	0.07651	0.07589	0.07621
365	0.07718	0.07645	0.07697
364	0.0781	0.07737	0.0777
363	0.0787	0.07811	0.07837
362	0.07941	0.07876	0.07893
361	0.08031	0.07956	0.07988
360	0.08122	0.08037	0.0807
359	0.08226	0.08144	0.08175

358	0.08347	0.08258	0.08287
357	0.08506	0.08417	0.08451
356	0.08674	0.08587	0.08594
355	0.08867	0.08764	0.0878
354	0.09083	0.08975	0.08984
353	0.09315	0.09197	0.09209
352	0.09559	0.09443	0.09447
351	0.09814	0.09689	0.09685
350	0.10071	0.09946	0.09936
349	0.10359	0.1012	0.10194
348	0.10744	0.1046	0.10426
347	0.10883	0.1067	0.10656
346	0.11021	0.10878	0.10931
345	0.11373	0.11188	0.11247
344	0.11738	0.11487	0.11531
343	0.11944	0.11729	0.11746
342	0.1218	0.12037	0.1201
341	0.12598	0.12467	0.12426
340	0.13084	0.12859	0.12858
339	0.13405	0.13207	0.13205
338	0.13919	0.13683	0.1367
337	0.14394	0.14173	0.1414
336	0.1492	0.14682	0.14667
335	0.15459	0.15224	0.15186
334	0.16028	0.15754	0.15701
333	0.16569	0.16265	0.16224
332	0.17182	0.16883	0.16836
331	0.17836	0.17502	0.17444
330	0.1849	0.18166	0.18096
329	0.19266	0.18944	0.18833
328	0.20173	0.19821	0.19715
327	0.21216	0.20819	0.20707
326	0.22395	0.2195	0.218
325	0.23741	0.23268	0.23074
324	0.25276	0.24746	0.24531
323	0.26925	0.26382	0.26167
322	0.28807	0.28255	0.27991
321	0.30916	0.30276	0.30019
320	0.33309	0.32647	0.32393
319	0.36031	0.35345	0.35132
318	0.39279	0.38589	0.38353
317	0.4316	0.42468	0.42288
316	0.47902	0.47175	0.47058
315	0.53697	0.52946	0.52851
314	0.6067	0.59838	0.59828
313	0.68603	0.67765	0.67827
312	0.77648	0.76732	0.76825

311	0.86148	0.86054	0.8617
310	0.95007	0.94943	0.95072
309	1.02648	1.02538	1.02726
308	1.08354	1.08141	1.08384
307	1.12394	1.11994	1.1221
306	1.15238	1.14587	1.14819
305	1.17554	1.16728	1.16979
304	1.19657	1.1898	1.19288
303	1.22313	1.21601	1.21863
302	1.24686	1.2421	1.2446
301	1.27096	1.26477	1.26785
300	1.29058	1.28439	1.28679
299	1.30673	1.30069	1.3046
298	1.32021	1.31574	1.3188
297	1.33493	1.32963	1.33246
296	1.3473	1.34104	1.34448
295	1.35634	1.3498	1.35315
294	1.36165	1.35486	1.35761
293	1.36179	1.35728	1.36022
292	1.36118	1.3578	1.3602
291	1.35954	1.35581	1.35719
290	1.3547	1.35245	1.35316
289	1.34628	1.34597	1.34728
288	1.3341	1.33421	1.33642
287	1.31783	1.31747	1.31928
286	1.29546	1.29562	1.29702
285	1.27041	1.26962	1.27091
284	1.2424	1.24063	1.24179
283	1.21205	1.21019	1.21039
282	1.18096	1.17957	1.18027
281	1.15168	1.15098	1.15091
280	1.12444	1.12346	1.12322
279	1.10147	1.09917	1.09844
278	1.0806	1.07811	1.07701
277	1.06222	1.05928	1.05827
276	1.04609	1.04332	1.04211
275	1.03157	1.02873	1.02705
274	1.01597	1.01328	1.01235
273	0.99903	0.99593	0.99489
272	0.97914	0.97618	0.97541
271	0.95839	0.95503	0.95383
270	0.93693	0.93407	0.93299
269	0.91772	0.91488	0.91381
268	0.89978	0.89685	0.89588
267	0.87974	0.87754	0.87679
266	0.85739	0.855	0.85452
265	0.83186	0.82973	0.82908

264	0.80335	0.80159	0.80102
263	0.77469	0.77269	0.77233
262	0.74579	0.74431	0.74425
261	0.71923	0.71755	0.71775
260	0.69569	0.6942	0.69418
259	0.67498	0.67381	0.67371
258	0.65779	0.6563	0.65642
257	0.64301	0.6418	0.64172
256	0.63125	0.63012	0.6305
255	0.62205	0.62136	0.62178
254	0.61546	0.61489	0.61517
253	0.61213	0.61141	0.61211
252	0.61298	0.61233	0.61295
251	0.61874	0.61823	0.61892
250	0.63086	0.63019	0.63126
249	0.64968	0.64961	0.65081
248	0.67707	0.67698	0.67808
247	0.71134	0.71123	0.71216
246	0.75339	0.75337	0.75449
245	0.80236	0.80188	0.80316
244	0.85707	0.85676	0.85813
243	0.91799	0.91749	0.91864
242	0.98516	0.98497	0.98585
241	1.05992	1.05911	1.06043
240	1.14299	1.14202	1.14322
239	1.23217	1.23129	1.23304
238	1.32933	1.32824	1.3304
237	1.42941	1.42755	1.43006
236	1.52687	1.52621	1.52861
235	1.61908	1.61839	1.62113
234	1.69758	1.69674	1.69948
233	1.763	1.76168	1.76307
232	1.8127	1.81108	1.81344
231	1.85413	1.85151	1.85323
230	1.88722	1.88527	1.88626
229	1.9132	1.90806	1.90978
228	1.92876	1.92429	1.92649
227	1.93466	1.93215	1.93214
226	1.92874	1.92382	1.92362
225	1.91333	1.91152	1.91068
224	1.89315	1.88967	1.88913
223	1.87229	1.86766	1.87079
222	1.85887	1.85374	1.85572
221	1.85661	1.85132	1.8505
220	1.86876	1.86386	1.86684

Table SI2: UV-Vis data of complex **15** after 0, 6, 25, 60 and 120 minutes photoirradiation.

Wavelength [nm]	Absorption before irradiation	Absorption after 6 min irradiation	Absorption after 25 min irradiation	Absorption after 60 min irradiation	Absorption after 120 min irradiation
750	1.81E-03	2.06E-03	2.41E-03	0.00246	0.00219
749	1.68E-03	2.03E-03	2.30E-03	0.00237	0.00207
748	1.76E-03	2.09E-03	2.35E-03	0.0023	0.00214
747	1.83E-03	2.17E-03	2.34E-03	0.00235	0.00215
746	1.82E-03	2.13E-03	2.37E-03	0.00232	0.00212
745	1.82E-03	2.18E-03	2.40E-03	0.00238	0.00209
744	1.78E-03	2.20E-03	2.37E-03	0.00238	0.00217
743	1.69E-03	2.12E-03	2.34E-03	0.00229	0.00212
742	1.71E-03	2.11E-03	2.26E-03	0.0023	0.00207
741	1.86E-03	2.16E-03	2.43E-03	0.00228	0.00207
740	1.77E-03	2.16E-03	2.34E-03	0.00227	0.00212
739	1.70E-03	2.00E-03	2.17E-03	0.00225	0.00195
738	1.61E-03	2.00E-03	2.28E-03	0.00226	0.00204
737	1.77E-03	2.17E-03	2.36E-03	0.00239	0.00216
736	1.73E-03	2.06E-03	2.38E-03	0.00229	0.00207
735	1.66E-03	2.10E-03	2.22E-03	0.00222	0.00199
734	1.64E-03	2.02E-03	2.24E-03	0.00226	0.00201
733	1.68E-03	2.08E-03	2.28E-03	0.00224	0.00205
732	1.74E-03	2.09E-03	2.32E-03	0.00234	0.00208
731	1.69E-03	2.12E-03	2.33E-03	0.00231	0.00212
730	1.79E-03	2.10E-03	2.30E-03	0.00228	0.00209
729	1.70E-03	2.07E-03	2.21E-03	0.00222	0.00205
728	1.76E-03	2.04E-03	2.19E-03	0.00228	0.00197
727	1.74E-03	1.97E-03	2.30E-03	0.00226	0.00194
726	1.64E-03	2.03E-03	2.25E-03	0.00227	0.00194
725	1.73E-03	2.07E-03	2.24E-03	0.00229	0.00208
724	1.62E-03	2.02E-03	2.25E-03	0.0022	0.002
723	1.65E-03	1.97E-03	2.17E-03	0.00221	0.00192
722	1.64E-03	2.00E-03	2.18E-03	0.00216	0.00199
721	1.67E-03	1.99E-03	2.18E-03	0.00228	0.00199
720	1.68E-03	2.05E-03	2.19E-03	0.00226	0.00204
719	1.65E-03	1.92E-03	2.24E-03	0.00214	0.00201
718	1.68E-03	1.98E-03	2.17E-03	0.00211	0.00191
717	1.70E-03	2.07E-03	2.28E-03	0.00227	0.00207
716	1.60E-03	1.99E-03	2.09E-03	0.00211	0.00191
715	1.68E-03	2.05E-03	2.16E-03	0.00218	0.00197
714	1.63E-03	1.91E-03	2.15E-03	0.00213	0.00189
713	1.60E-03	1.98E-03	2.18E-03	0.00223	0.00193
712	1.56E-03	1.98E-03	2.14E-03	0.00217	0.00193
711	1.62E-03	2.03E-03	2.17E-03	0.00217	0.00192
710	1.65E-03	1.98E-03	2.10E-03	0.00205	0.00186
709	1.60E-03	1.96E-03	2.10E-03	0.00224	0.00193
708	1.65E-03	1.93E-03	2.10E-03	0.00217	0.00184
707	1.51E-03	1.92E-03	2.12E-03	0.00205	0.00177

706	1.55E-03	1.92E-03	2.05E-03	0.00197	0.00181
705	1.56E-03	1.90E-03	2.13E-03	0.00213	0.00182
704	1.59E-03	1.87E-03	2.06E-03	0.0021	0.00176
703	1.48E-03	1.90E-03	2.01E-03	0.00205	0.00176
702	1.55E-03	1.90E-03	2.05E-03	0.00206	0.00177
701	1.55E-03	1.91E-03	2.14E-03	0.00209	0.00181
700	1.55E-03	1.93E-03	2.10E-03	0.00207	0.00177
699	1.51E-03	1.77E-03	2.02E-03	0.00197	0.00169
698	1.57E-03	1.91E-03	2.11E-03	0.00209	0.00172
697	1.47E-03	1.84E-03	2.07E-03	0.00201	0.00174
696	1.56E-03	1.88E-03	2.07E-03	0.00209	0.00179
695	1.51E-03	1.92E-03	2.12E-03	0.00202	0.00174
694	1.49E-03	1.88E-03	2.02E-03	0.00205	0.00177
693	1.56E-03	1.90E-03	2.10E-03	0.00209	0.00174
692	1.48E-03	1.79E-03	2.00E-03	0.002	0.00168
691	1.52E-03	1.91E-03	2.00E-03	0.002	0.00174
690	1.52E-03	1.86E-03	2.04E-03	0.00206	0.00171
689	1.52E-03	1.85E-03	2.01E-03	0.00203	0.00169
688	1.50E-03	1.84E-03	2.05E-03	0.00199	0.00173
687	1.47E-03	1.84E-03	2.06E-03	0.00203	0.00165
686	1.48E-03	1.90E-03	2.04E-03	0.002	0.0017
685	1.45E-03	1.80E-03	2.03E-03	0.00204	0.00164
684	1.55E-03	1.90E-03	2.02E-03	0.00203	0.00168
683	1.49E-03	1.77E-03	2.00E-03	0.00194	0.00161
682	1.46E-03	1.81E-03	2.00E-03	0.00193	0.00164
681	1.48E-03	1.84E-03	1.97E-03	0.00195	0.00165
680	1.51E-03	1.82E-03	2.00E-03	0.00191	0.00161
679	1.53E-03	1.84E-03	2.03E-03	0.002	0.00172
678	1.54E-03	1.85E-03	2.01E-03	0.002	0.00162
677	1.50E-03	1.82E-03	2.02E-03	0.00204	0.00165
676	1.50E-03	1.85E-03	2.03E-03	0.00195	0.00166
675	1.48E-03	1.84E-03	2.01E-03	0.00194	0.00165
674	1.53E-03	1.87E-03	2.02E-03	0.00197	0.00167
673	1.51E-03	1.83E-03	2.01E-03	0.002	0.00167
672	1.55E-03	1.88E-03	2.03E-03	0.00203	0.00167
671	1.51E-03	1.82E-03	2.03E-03	0.00197	0.00163
670	1.53E-03	1.86E-03	2.05E-03	0.002	0.00164
669	1.51E-03	1.83E-03	1.96E-03	0.00197	0.00165
668	1.51E-03	1.83E-03	1.98E-03	0.00195	0.00164
667	1.53E-03	1.86E-03	2.04E-03	0.00197	0.00166
666	1.57E-03	1.89E-03	2.08E-03	0.00204	0.00166
665	1.60E-03	1.94E-03	2.09E-03	0.00207	0.0017
664	1.64E-03	1.98E-03	2.12E-03	0.00211	0.00175
663	1.62E-03	1.98E-03	2.10E-03	0.00206	0.00176
662	1.60E-03	1.94E-03	2.12E-03	0.00202	0.00171
661	1.63E-03	1.97E-03	2.13E-03	0.00203	0.0017
660	1.71E-03	1.99E-03	2.16E-03	0.0021	0.00171

659	1.69E-03	1.99E-03	2.16E-03	0.00218	0.00172
658	1.65E-03	1.97E-03	2.16E-03	0.00211	0.00176
657	1.70E-03	1.98E-03	2.17E-03	0.00206	0.00178
656	1.71E-03	2.06E-03	2.21E-03	0.00214	0.0018
655	1.80E-03	2.05E-03	2.21E-03	0.00218	0.00179
654	1.76E-03	2.10E-03	2.24E-03	0.00217	0.00181
653	1.80E-03	2.10E-03	2.26E-03	0.0022	0.00186
652	1.82E-03	2.13E-03	2.25E-03	0.00222	0.00184
651	1.85E-03	2.18E-03	2.31E-03	0.00224	0.00185
650	1.91E-03	2.23E-03	2.38E-03	0.00232	0.00198
649	1.93E-03	2.25E-03	2.40E-03	0.00231	0.00192
648	1.97E-03	2.25E-03	2.39E-03	0.00235	0.00192
647	2.07E-03	2.37E-03	2.47E-03	0.00243	0.00203
646	2.06E-03	2.30E-03	2.47E-03	0.00244	0.00206
645	2.07E-03	2.40E-03	2.53E-03	0.00245	0.00213
644	2.09E-03	2.40E-03	2.57E-03	0.00251	0.00212
643	2.19E-03	2.47E-03	2.63E-03	0.0025	0.00214
642	2.23E-03	2.52E-03	2.65E-03	0.00259	0.00219
641	2.27E-03	2.54E-03	2.67E-03	0.00264	0.00222
640	2.33E-03	2.64E-03	2.79E-03	0.00271	0.00231
639	2.41E-03	2.69E-03	2.86E-03	0.00279	0.00237
638	2.44E-03	2.75E-03	2.89E-03	0.00276	0.00236
637	2.51E-03	2.82E-03	2.95E-03	0.00289	0.00245
636	2.63E-03	2.88E-03	2.99E-03	0.00293	0.00252
635	2.69E-03	2.92E-03	3.09E-03	0.00303	0.00263
634	2.76E-03	3.06E-03	3.20E-03	0.00312	0.0027
633	2.83E-03	3.10E-03	3.26E-03	0.00318	0.00277
632	2.86E-03	3.18E-03	3.32E-03	0.00318	0.00281
631	2.99E-03	3.26E-03	3.39E-03	0.0033	0.00287
630	3.10E-03	3.37E-03	3.47E-03	0.00339	0.00298
629	3.21E-03	3.48E-03	3.58E-03	0.00353	0.00304
628	3.27E-03	3.56E-03	3.60E-03	0.00356	0.00311
627	3.40E-03	3.65E-03	3.77E-03	0.00368	0.00316
626	3.50E-03	3.69E-03	3.85E-03	0.00378	0.00329
625	3.60E-03	3.89E-03	3.97E-03	0.00383	0.00345
624	3.78E-03	4.02E-03	4.10E-03	0.00405	0.00356
623	0.00384	0.00412	4.21E-03	0.00411	0.00369
622	0.004	0.0043	0.00432	0.00421	0.00374
621	0.00419	0.00436	0.0045	0.00429	0.00385
620	0.00419	0.00451	0.00461	0.00439	0.00391
619	0.00444	0.00464	0.00469	0.00459	0.00407
618	0.00457	0.0048	0.00488	0.00474	0.00417
617	0.0048	0.00498	0.00509	0.00497	0.0044
616	0.00498	0.00512	0.0052	0.00507	0.00452
615	0.00513	0.0053	0.00538	0.00524	0.00474
614	0.00523	0.00544	0.00554	0.00536	0.00482
613	0.00546	0.0057	0.00576	0.00558	0.00505

612	0.00567	0.00584	0.00591	0.00576	0.00523
611	0.00587	0.00607	0.0061	0.00595	0.00537
610	0.00613	0.00626	0.00635	0.00622	0.0056
609	0.00626	0.00641	0.00645	0.00631	0.00574
608	0.00653	0.00666	0.00674	0.00655	0.00598
607	0.00673	0.0069	0.00693	0.00677	0.00624
606	0.00698	0.00712	0.00714	0.00699	0.00647
605	0.00722	0.00735	0.00736	0.00719	0.00664
604	0.00751	0.00763	0.00764	0.00746	0.0069
603	0.00778	0.00786	0.00788	0.00772	0.00715
602	0.00801	0.00813	0.00813	0.00792	0.00739
601	0.00839	0.0085	0.00846	0.00825	0.00775
600	0.00865	0.00876	0.00872	0.00854	0.00793
599	0.00895	0.009	0.00896	0.00879	0.0082
598	0.00924	0.00931	0.00921	0.00904	0.0085
597	0.00954	0.00961	0.00957	0.00934	0.00879
596	0.00988	0.00993	0.00992	0.00966	0.00913
595	0.01018	0.01024	0.01019	0.00997	0.00943
594	0.01052	0.01057	0.01049	0.01026	0.00973
593	0.01094	0.01095	0.01084	0.01063	0.01005
592	0.01125	0.01129	0.01115	0.01095	0.01039
591	0.01163	0.01163	0.01154	0.01128	0.01069
590	0.01199	0.01195	0.01187	0.01164	0.01105
589	0.0123	0.01229	0.01216	0.01188	0.01132
588	0.01273	0.0127	0.01254	0.01229	0.01173
587	0.0131	0.01305	0.01291	0.01264	0.01206
586	0.01357	0.01345	0.01332	0.01304	0.01242
585	0.01393	0.01389	0.01372	0.01346	0.01283
584	0.01439	0.01426	0.01415	0.01383	0.01321
583	0.01479	0.01471	0.01445	0.01416	0.01354
582	0.01518	0.0151	0.0149	0.01455	0.01397
581	0.01568	0.01556	0.01529	0.01504	0.01442
580	0.01616	0.01592	0.01567	0.01542	0.01477
579	0.01656	0.01636	0.0162	0.01587	0.01519
578	0.01698	0.01685	0.01661	0.0162	0.01558
577	0.0175	0.01735	0.01711	0.01668	0.01605
576	0.01794	0.01775	0.01746	0.01706	0.01642
575	0.01843	0.01824	0.01795	0.01758	0.01695
574	0.01891	0.01872	0.0184	0.018	0.01734
573	0.01944	0.0192	0.01889	0.01853	0.01783
572	0.01986	0.0196	0.01924	0.01888	0.01819
571	0.0204	0.02012	0.01979	0.01937	0.01871
570	0.02083	0.02061	0.02024	0.0199	0.01919
569	0.02138	0.02108	0.02075	0.02037	0.01964
568	0.02185	0.0215	0.02116	0.02083	0.02008
567	0.0224	0.02211	0.02162	0.02126	0.02053
566	0.02286	0.02254	0.02201	0.02167	0.02094

565	0.02337	0.02302	0.02255	0.02213	0.02141
564	0.02381	0.02345	0.02297	0.02252	0.02178
563	0.02439	0.02401	0.02351	0.02304	0.02236
562	0.02489	0.02451	0.02396	0.02356	0.0228
561	0.02538	0.02497	0.02444	0.02401	0.02324
560	0.02587	0.02554	0.02491	0.02451	0.02374
559	0.02641	0.026	0.02544	0.02496	0.02419
558	0.02683	0.02647	0.02594	0.02541	0.02462
557	0.02739	0.02696	0.02635	0.02587	0.02516
556	0.02788	0.02742	0.02678	0.02632	0.02559
555	0.02837	0.02792	0.0273	0.02679	0.02608
554	0.02887	0.0284	0.02775	0.02727	0.0265
553	0.02941	0.02887	0.02825	0.02774	0.02697
552	0.02984	0.02934	0.02874	0.0282	0.02743
551	0.03032	0.02979	0.02917	0.02865	0.02785
550	0.03081	0.03032	0.02967	0.02914	0.02836
549	0.03128	0.03075	0.03005	0.02956	0.02874
548	0.03177	0.03121	0.03048	0.03003	0.02923
547	0.03222	0.03162	0.03095	0.03043	0.0296
546	0.03263	0.03202	0.03133	0.03082	0.03001
545	0.03308	0.03253	0.03181	0.03128	0.03046
544	0.03353	0.03291	0.0322	0.0317	0.03084
543	0.034	0.03342	0.03264	0.03218	0.03128
542	0.03441	0.03377	0.03301	0.03249	0.03168
541	0.03487	0.03424	0.03354	0.03298	0.03213
540	0.03524	0.03463	0.03388	0.03335	0.03247
539	0.03565	0.03508	0.03432	0.03376	0.03287
538	0.03603	0.03543	0.03462	0.03408	0.03326
537	0.03649	0.03586	0.03506	0.03448	0.03365
536	0.03679	0.03615	0.03541	0.03489	0.03398
535	0.03723	0.03658	0.03591	0.03531	0.03443
534	0.03761	0.03699	0.03631	0.03572	0.03487
533	0.03801	0.03733	0.03666	0.03609	0.03519
532	0.0384	0.03771	0.03703	0.03646	0.03555
531	0.0387	0.03804	0.03737	0.03678	0.03587
530	0.03919	0.03846	0.03772	0.03718	0.03625
529	0.03948	0.03879	0.03804	0.03749	0.0366
528	0.03983	0.03909	0.0384	0.03784	0.03689
527	0.04018	0.03946	0.03878	0.03817	0.03726
526	0.04048	0.03981	0.03911	0.03851	0.03759
525	0.04084	0.04017	0.03944	0.03882	0.03786
524	0.04123	0.04053	0.03975	0.0392	0.03813
523	0.04153	0.04081	0.04008	0.03947	0.03847
522	0.04191	0.04116	0.04045	0.03979	0.03879
521	0.04219	0.04146	0.04077	0.04003	0.03906
520	0.04243	0.04178	0.041	0.0403	0.03934
519	0.04288	0.04214	0.04133	0.04069	0.03974

518	0.04318	0.04249	0.04159	0.04101	0.04003
517	0.04351	0.04275	0.04187	0.04131	0.04035
516	0.04381	0.04308	0.0423	0.04164	0.04068
515	0.04419	0.04345	0.04274	0.04204	0.04109
514	0.04451	0.04378	0.04301	0.0424	0.04142
513	0.04491	0.04419	0.04344	0.04276	0.04179
512	0.04531	0.04453	0.04376	0.04314	0.04212
511	0.04569	0.04491	0.04412	0.04352	0.04249
510	0.04608	0.04534	0.04458	0.04391	0.04291
509	0.04648	0.0457	0.04492	0.04428	0.04326
508	0.04687	0.04614	0.04542	0.0447	0.0437
507	0.0473	0.04654	0.04576	0.04509	0.04408
506	0.04777	0.04704	0.0462	0.04556	0.04451
505	0.0483	0.04752	0.04671	0.0461	0.045
504	0.04879	0.04795	0.04721	0.04652	0.04546
503	0.04933	0.04852	0.04779	0.04705	0.04598
502	0.0498	0.04907	0.04828	0.04754	0.04648
501	0.05043	0.04965	0.04887	0.0481	0.04702
500	0.05101	0.05026	0.04943	0.0487	0.04761
499	0.05169	0.05092	0.05006	0.04931	0.0482
498	0.05244	0.0516	0.05076	0.04999	0.04887
497	0.05316	0.0523	0.05146	0.05077	0.04959
496	0.05393	0.05304	0.05221	0.05139	0.0503
495	0.05466	0.0538	0.05296	0.05216	0.05102
494	0.05549	0.05462	0.0537	0.0529	0.05183
493	0.05641	0.0555	0.05459	0.05381	0.05261
492	0.05735	0.05641	0.05549	0.05471	0.05348
491	0.05828	0.05733	0.0564	0.05555	0.05435
490	0.05926	0.05832	0.05736	0.05653	0.05528
489	0.06029	0.05936	0.05836	0.05748	0.05625
488	0.06133	0.06036	0.05932	0.05849	0.05722
487	0.06247	0.06145	0.06047	0.05958	0.05831
486	0.06361	0.06255	0.06154	0.06062	0.05931
485	0.06483	0.06378	0.06274	0.06176	0.06046
484	0.06601	0.06495	0.06384	0.0629	0.06161
483	0.06722	0.06613	0.06501	0.06409	0.06271
482	0.06848	0.06736	0.0662	0.06522	0.06387
481	0.0698	0.06865	0.06744	0.06643	0.06505
480	0.07103	0.06988	0.06861	0.06764	0.0662
479	0.07244	0.07124	0.06993	0.06891	0.06749
478	0.07372	0.07245	0.07124	0.07009	0.06864
477	0.075	0.07374	0.07244	0.0714	0.06988
476	0.07637	0.07512	0.07376	0.07264	0.07114
475	0.07765	0.07636	0.07492	0.07388	0.07238
474	0.0791	0.07773	0.07632	0.07517	0.0736
473	0.08041	0.07901	0.07756	0.07641	0.07483
472	0.0818	0.08036	0.07892	0.07771	0.07612

471	0.0831	0.08163	0.08017	0.07891	0.07729
470	0.08442	0.08293	0.08137	0.08015	0.07857
469	0.08583	0.08432	0.0827	0.08145	0.07977
468	0.08716	0.0856	0.08395	0.08275	0.08103
467	0.08849	0.08686	0.08527	0.08393	0.08226
466	0.08978	0.08816	0.08651	0.08517	0.08344
465	0.09104	0.08938	0.08773	0.08636	0.08465
464	0.09237	0.09068	0.08896	0.08759	0.0859
463	0.09364	0.09193	0.09014	0.08878	0.08702
462	0.09494	0.0932	0.09139	0.08999	0.08826
461	0.0963	0.09447	0.09269	0.09123	0.08942
460	0.09749	0.09566	0.09388	0.0924	0.09057
459	0.0987	0.09679	0.09495	0.09345	0.09167
458	0.09998	0.09807	0.09604	0.09457	0.09274
457	0.10124	0.09935	0.09735	0.09576	0.09392
456	0.1024	0.10048	0.09854	0.09695	0.09501
455	0.10364	0.10174	0.09978	0.09821	0.09627
454	0.10483	0.10283	0.10089	0.09928	0.09735
453	0.10596	0.10396	0.10196	0.10031	0.09842
452	0.10709	0.10508	0.10302	0.10137	0.0995
451	0.10826	0.10625	0.10416	0.1025	0.10057
450	0.10931	0.10714	0.10506	0.10344	0.1015
449	0.11029	0.1082	0.10603	0.10439	0.1024
448	0.11143	0.10936	0.10713	0.10536	0.10338
447	0.11242	0.11024	0.10817	0.10643	0.10431
446	0.11336	0.11122	0.10907	0.10738	0.10532
445	0.1143	0.1122	0.10995	0.10822	0.10624
444	0.11513	0.113	0.11081	0.10906	0.10704
443	0.11603	0.11385	0.11155	0.1099	0.10784
442	0.11682	0.11461	0.1123	0.11051	0.10848
441	0.11744	0.11525	0.11297	0.11119	0.10904
440	0.11815	0.11586	0.11366	0.11185	0.10978
439	0.11864	0.11644	0.1142	0.11239	0.11034
438	0.11912	0.11695	0.11461	0.11287	0.11076
437	0.11961	0.11732	0.11498	0.11323	0.11114
436	0.11992	0.11765	0.1154	0.11356	0.11136
435	0.12019	0.11793	0.11565	0.11386	0.11167
434	0.12034	0.11809	0.11584	0.11405	0.11199
433	0.12041	0.11819	0.11595	0.11406	0.11193
432	0.12044	0.11825	0.11594	0.1141	0.11204
431	0.12042	0.11819	0.11591	0.11415	0.11209
430	0.12025	0.11793	0.11573	0.11394	0.1118
429	0.12001	0.11782	0.11561	0.11374	0.11157
428	0.11969	0.11758	0.11531	0.11355	0.11144
427	0.11936	0.11726	0.11502	0.1133	0.11122
426	0.11899	0.11693	0.1147	0.11283	0.11079
425	0.11859	0.11646	0.11432	0.11252	0.11046

424	0.11809	0.11604	0.11391	0.11221	0.11007
423	0.11749	0.11547	0.11337	0.11157	0.10952
422	0.11699	0.115	0.11292	0.11112	0.10909
421	0.11647	0.11447	0.1124	0.11069	0.10868
420	0.11589	0.11392	0.11186	0.11026	0.10804
419	0.1154	0.1134	0.11141	0.10974	0.1077
418	0.1148	0.11294	0.11083	0.10916	0.10716
417	0.11413	0.11224	0.11032	0.10867	0.10664
416	0.1136	0.11179	0.10986	0.10822	0.10622
415	0.11304	0.11129	0.10939	0.10772	0.10565
414	0.11256	0.11083	0.10891	0.10731	0.10526
413	0.11207	0.11031	0.10853	0.1068	0.10483
412	0.11155	0.10981	0.10798	0.1064	0.10442
411	0.1111	0.10943	0.10756	0.10598	0.10401
410	0.11062	0.10898	0.10733	0.10561	0.10366
409	0.11024	0.10859	0.1068	0.10528	0.10331
408	0.10977	0.10822	0.10635	0.10498	0.10298
407	0.10942	0.10781	0.10609	0.10457	0.10265
406	0.10923	0.10759	0.10596	0.10444	0.10249
405	0.10893	0.10734	0.10566	0.10421	0.10227
404	0.10866	0.10716	0.10547	0.10403	0.102
403	0.10833	0.10683	0.10527	0.10377	0.10186
402	0.10822	0.10669	0.10512	0.10364	0.10168
401	0.10814	0.10666	0.10504	0.10362	0.10173
400	0.10814	0.10665	0.10502	0.10364	0.1017
399	0.10808	0.10661	0.10506	0.10361	0.10175
398	0.1081	0.10674	0.10521	0.10385	0.10192
397	0.10814	0.1068	0.10529	0.10386	0.10199
396	0.10827	0.10698	0.10543	0.10407	0.10224
395	0.1084	0.10716	0.10571	0.10432	0.10253
394	0.10871	0.10746	0.106	0.10472	0.10286
393	0.10899	0.10774	0.10636	0.10505	0.10324
392	0.10925	0.10801	0.10671	0.10536	0.10353
391	0.10961	0.10846	0.10699	0.10579	0.10392
390	0.11011	0.10891	0.10761	0.10628	0.10441
389	0.11053	0.10936	0.10806	0.10668	0.10498
388	0.11112	0.10993	0.10863	0.10729	0.10553
387	0.11167	0.11056	0.10919	0.10788	0.10615
386	0.11228	0.11113	0.10988	0.1086	0.10672
385	0.11292	0.11182	0.11049	0.10918	0.10741
384	0.11353	0.11246	0.1111	0.10985	0.10803
383	0.11439	0.11326	0.11197	0.11074	0.10894
382	0.11518	0.11408	0.11278	0.11161	0.1098
381	0.11612	0.11486	0.11363	0.11246	0.11073
380	0.117	0.11585	0.11463	0.1134	0.11158
379	0.11797	0.11681	0.11563	0.11443	0.11266
378	0.11898	0.11792	0.11663	0.11545	0.11382

377	0.12015	0.11897	0.11768	0.11652	0.11495
376	0.1214	0.1202	0.11904	0.11792	0.11623
375	0.12269	0.12154	0.12049	0.11932	0.11774
374	0.12399	0.12291	0.12187	0.12072	0.11921
373	0.12542	0.12445	0.1232	0.12219	0.12072
372	0.12696	0.1259	0.1248	0.12371	0.12229
371	0.12858	0.12764	0.12641	0.12546	0.12405
370	0.13021	0.12917	0.12807	0.12711	0.12584
369	0.13193	0.13087	0.12984	0.12899	0.12769
368	0.13369	0.13261	0.13156	0.13063	0.12949
367	0.13546	0.13454	0.13347	0.13261	0.13149
366	0.13743	0.13638	0.13542	0.13473	0.13358
365	0.13967	0.13861	0.13765	0.13686	0.13584
364	0.14214	0.14111	0.14014	0.13946	0.13852
363	0.14503	0.14403	0.14309	0.14246	0.14154
362	0.14818	0.14718	0.1464	0.14576	0.14495
361	0.15203	0.15104	0.1503	0.14955	0.14886
360	0.15628	0.15542	0.15461	0.1539	0.15334
359	0.16099	0.16012	0.15926	0.15886	0.15824
358	0.16589	0.16499	0.16403	0.16373	0.16323
357	0.17078	0.1698	0.16916	0.16866	0.16826
356	0.17543	0.17448	0.17383	0.17345	0.17321
355	0.17972	0.17876	0.17805	0.17776	0.17757
354	0.18418	0.18331	0.1825	0.18242	0.18232
353	0.18875	0.18789	0.18724	0.18717	0.18727
352	0.19397	0.19312	0.19249	0.1924	0.19257
351	0.19989	0.19905	0.19838	0.19837	0.19867
350	0.20635	0.20544	0.20488	0.20488	0.20534
349	0.21243	0.21143	0.21038	0.21121	0.21065
348	0.22289	0.22206	0.22172	0.22125	0.2212
347	0.23163	0.23085	0.23145	0.23024	0.23081
346	0.2408	0.24003	0.23954	0.23918	0.23981
345	0.25233	0.25155	0.25026	0.24994	0.2507
344	0.26572	0.26447	0.26405	0.26339	0.26412
343	0.27934	0.27839	0.27827	0.27811	0.27844
342	0.29231	0.29121	0.29094	0.29132	0.29174
341	0.30596	0.30452	0.30449	0.30427	0.30478
340	0.31792	0.31683	0.3162	0.3154	0.3166
339	0.32761	0.32693	0.32609	0.3262	0.32686
338	0.33604	0.33519	0.33474	0.33434	0.33557
337	0.3436	0.3428	0.34189	0.34204	0.34366
336	0.35214	0.351	0.35037	0.35088	0.35266
335	0.36153	0.3603	0.35996	0.36049	0.36257
334	0.37235	0.3714	0.37127	0.37175	0.37417
333	0.385	0.38369	0.38378	0.38462	0.38703
332	0.39982	0.39874	0.39869	0.39936	0.40276
331	0.41673	0.41574	0.41546	0.41655	0.42031

330	0.43737	0.43582	0.43586	0.43751	0.44152
329	0.46032	0.45898	0.4596	0.46151	0.46601
328	0.48837	0.48732	0.48775	0.4897	0.49514
327	0.51967	0.51841	0.51924	0.52104	0.52741
326	0.55335	0.55154	0.55265	0.55538	0.56248
325	0.58926	0.5876	0.58881	0.59146	0.59995
324	0.62565	0.62405	0.62514	0.62857	0.63744
323	0.66319	0.66156	0.66255	0.66653	0.67712
322	0.70313	0.70072	0.70236	0.70647	0.71785
321	0.74602	0.74365	0.74462	0.74919	0.76143
320	0.79398	0.79141	0.79214	0.79645	0.80961
319	0.84946	0.84629	0.8463	0.8514	0.86444
318	0.91309	0.90928	0.90927	0.91364	0.92661
317	0.98757	0.98365	0.98201	0.98693	0.99936
316	1.07609	1.07144	1.0696	1.07249	1.08541
315	1.17975	1.17414	1.1702	1.17422	1.18591
314	1.29995	1.29227	1.28836	1.29049	1.30247
313	1.43373	1.42489	1.41976	1.42206	1.43417
312	1.5827	1.57071	1.56378	1.56637	1.57971
311	1.73744	1.7273	1.71635	1.71668	1.72724
310	1.89905	1.88121	1.87578	1.87334	1.8895
309	2.0535	2.03759	2.02746	2.02897	2.04127
308	2.21389	2.20209	2.18667	2.185	2.19527
307	2.37129	2.35535	2.34051	2.3317	2.35284
306	2.52929	2.5264	2.50353	2.49823	2.5056
305	2.68721	2.68288	2.64673	2.62738	2.65
304	2.8505	2.81505	2.79199	2.76109	2.799
303	2.97916	2.93146	2.93625	2.8622	2.88129
302	3.03997	3.02976	3.01712	2.92828	2.91943
301	3.12881	3.05553	3.07882	2.98752	2.93646
300	3.14512	3.12629	3.07804	3.03773	3.02039
299	3.12222	3.14133	3.11773	3.05718	3.04773
298	3.17349	3.19632	3.13876	3.08438	3.05854
297	3.20052	3.17376	3.13311	3.02062	3.04721
296	3.21625	3.22382	3.18469	3.11392	3.07324
295	3.21179	3.21788	3.19007	3.10458	3.10956
294	3.30109	3.31584	3.19788	3.07795	3.15469
293	3.23109	3.27259	3.22791	3.14459	3.14165
292	3.25181	3.20104	3.23438	3.18793	3.16276
291	3.334	3.26699	3.21558	3.19881	3.14092
290	3.35061	3.2814	3.29038	3.17687	3.16709
289	3.34431	3.31882	3.26682	3.18641	3.20726
288	3.29072	3.30237	3.29689	3.21367	3.21452
287	3.3442	3.36022	3.3705	3.21635	3.20981
286	3.3833	3.45496	3.35672	3.21393	3.25933
285	3.33541	3.57932	3.31657	3.24959	3.21796
284	3.36973	3.50518	3.30564	3.22345	3.20144

283	3.30735	4.6392	3.24665	3.194	3.21559
282	3.27843	3.62403	3.29523	3.19789	3.19746
281	3.31897	3.41942	3.24833	3.19554	3.19718
280	3.29248	3.52668	3.20829	3.18674	3.17512
279	3.26373	3.51869	3.2423	3.1659	3.17905
278	3.21195	3.37145	3.24151	3.17219	3.17199
277	3.21475	3.2774	3.18377	3.12686	3.13734
276	3.25324	3.22804	3.20214	3.13211	3.10621
275	3.21014	3.22419	3.16077	3.13909	3.09292
274	3.1709	3.18502	3.139	3.11458	3.13964
273	3.14035	3.21491	3.124	3.10622	3.06319
272	3.1112	3.1647	3.11233	3.11803	3.05723
271	3.08902	3.05044	3.07128	3.05061	3.08372
270	3.11185	3.04282	3.06208	3.04337	3.03305
269	3.05613	3.10975	3.02599	3.01792	2.99257
268	3.02012	3.00604	2.99591	2.98297	2.97626
267	2.95774	2.98152	2.93627	2.94316	2.94607
266	2.91829	2.92294	2.90131	2.88947	2.89201
265	2.87837	2.89838	2.87096	2.84674	2.86854
264	2.82836	2.81608	2.82417	2.81802	2.81458
263	2.77554	2.7313	2.78867	2.77767	2.77313
262	2.73918	2.69563	2.71683	2.71627	2.71602
261	2.6807	2.65089	2.66844	2.65561	2.66594
260	2.61891	2.59303	2.61352	2.59517	2.60483
259	2.55633	2.54233	2.5552	2.55702	2.55216
258	2.50674	2.50384	2.50266	2.49665	2.50299
257	2.46088	2.4659	2.45748	2.45527	2.45332
256	2.43245	2.4269	2.43009	2.42318	2.42645
255	2.42224	2.41159	2.41342	2.40725	2.40506
254	2.42142	2.41955	2.42048	2.41395	2.41057
253	2.43765	2.44104	2.44133	2.43151	2.42815
252	2.48725	2.48388	2.4793	2.47302	2.46924
251	2.53968	2.55175	2.53638	2.52709	2.5277
250	2.61999	2.63545	2.61655	2.60284	2.60815
249	2.71655	2.73331	2.70779	2.70355	2.70522
248	2.83606	2.8537	2.83391	2.8187	2.82858
247	2.98292	3.00913	2.96047	2.94712	2.94083
246	3.13577	3.21536	3.13552	3.09126	3.08975
245	3.2817	3.4343	3.27616	3.21405	3.19352
244	3.43073	3.59488	3.35661	3.32828	3.32566
243	3.48731	3.72126	3.48735	3.37472	3.40942
242	3.56927	3.73018	3.56101	3.46279	3.48785
241	3.63481	4.17702	3.57772	3.47142	3.47164
240	3.65077	3.95821	3.64564	3.49378	3.55197
239	3.72959	3.95401	3.60581	3.54487	3.49559
238	3.69096	3.67707	3.554	3.55191	3.50053
237	3.6727	3.63635	3.67595	3.562	3.53144

236	3.68275	3.66374	3.57358	3.55622	3.52526
235	3.6392	3.76789	3.57046	3.5416	3.49835
234	3.64212	3.65816	3.55262	3.53397	3.50245
233	3.62529	3.66562	3.5918	3.48042	3.46854
232	3.65775	3.61407	3.57447	3.51077	3.53543
231	3.59879	3.46724	3.55259	3.49939	3.45765
230	3.67739	3.52781	3.60564	3.49652	3.53628
229	3.63603	3.52275	3.59006	3.52117	3.47702
228	3.60925	3.66613	3.5603	3.41461	3.47867
227	3.55174	3.65488	3.5788	3.50431	3.49178
226	3.64724	3.68299	3.53808	3.53167	3.4533
225	3.64914	3.50055	3.52065	3.43466	3.50253
224	3.64229	3.49292	3.55342	3.43767	3.44659
223	3.54244	3.63704	3.53928	3.49207	3.45375
222	3.54817	3.59049	3.47952	3.42542	3.44556
221	3.58572	3.65803	3.48654	3.44533	3.36517
220	3.54737	3.62303	3.48656	3.45621	3.38905

Electrochemical investigations

Cyclic voltammetry (CV) and square wave voltammetry (SWV) experiments were performed with an AutoLab PGSTAT302 potentiostat-galvanostat controlled by resident NOVA 2.1.3 software.

A spectroelectrochemical cuvette from ALS Japan was used as electrochemical cell to scope with the very limited amount of available material. Two platinum wires served as working and auxiliary electrode respectively, and a silver/silver chloride electrode served as reference.

The measurements were conducted in HPLC grade acetonitrile (CH_3CN) and tetrabutyl-ammonium hexafluorophosphate (Bu_4NPF_6) was used as supporting electrolyte at a concentration of 0.1 M. All recorded potentials are given relative to Ag/AgCl/3M KCl. In all the experiments, the scan rate was 100 mV/s for CV and the pulse frequency was 15 Hz for SWV.

For all complexes (**1**, **2**, **3**, **4**, **5** and **15**) no redox signals at negative potentials were recorded, indicating that the ligand-centered reduction steps occur outside of the potential window accessible with our set-up and skills (-1.5 to 1.5 V). And indeed, the reduction of copper(I) diimine complexes is reported in literature between -1.5 and -1.7 V vs. SCE,[3] corresponding to values between -1.532 and -1.732 V vs. Ag/AgCl/3M KCl.

The recorded voltagramms of the complexes **1**, **2**, **3**, **4**, **5** and **15** are displayed in figure SI5 and the extracted redox values are listed in table SI3. Due to the limited stability and/or isolation properties of the complexes **2**-**5**, the redox studies were performed with the best available sample quality. However, the appearance of additional oxidation waves in **5** and even more pronounced in **2**, is most likely rather due to impurities than due to intrinsic redox features of the parent complexes.

[3]Armaroli, *Chem. Soc. Rev.*, **2001**, 30, 113-124.

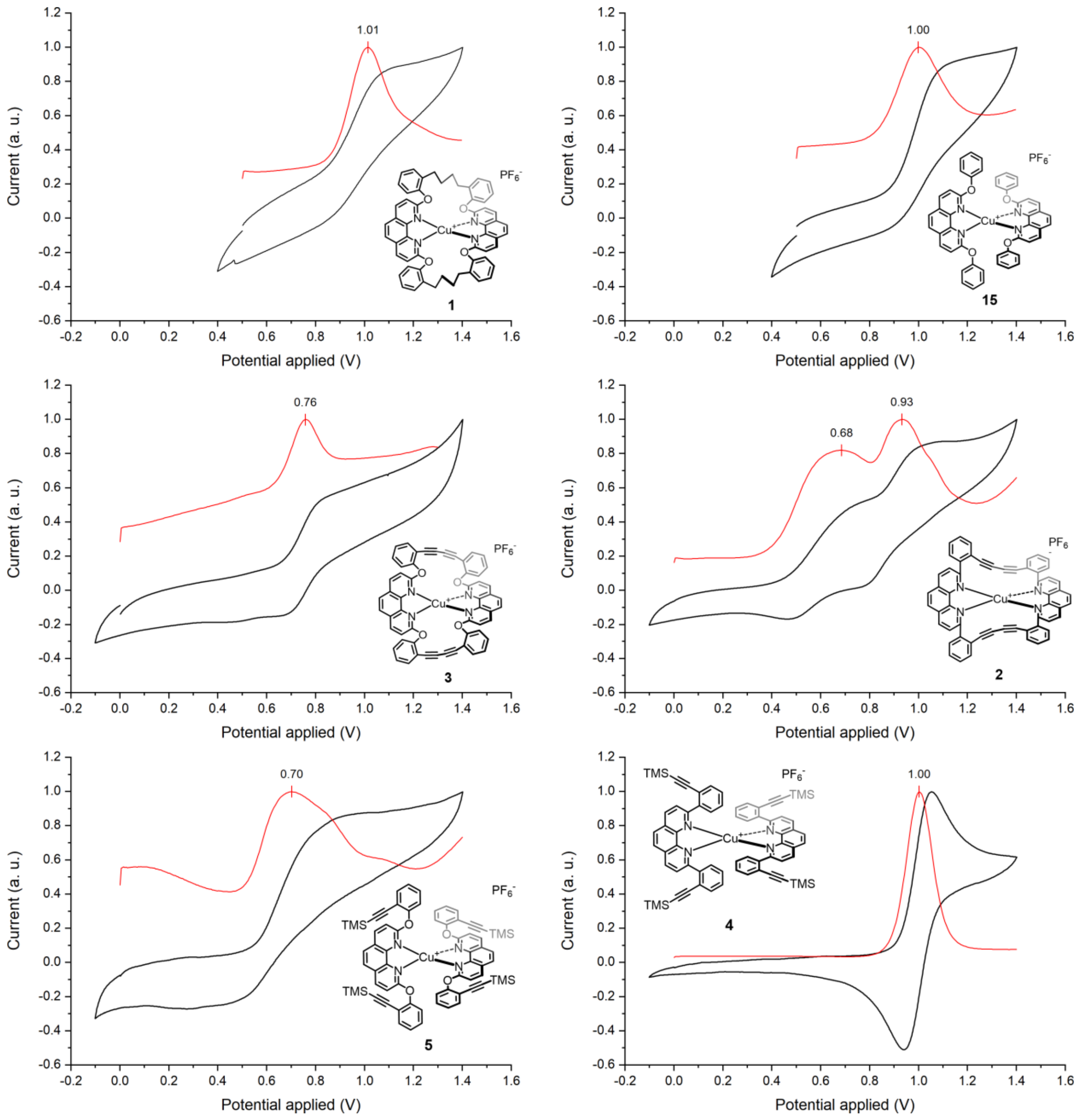

Figure SI5: CV (black lines) and SWV (red lines) voltamogramms recorded in 0.1M n-Bu_4NPF_6/CH_3CN at 25 °C. Potentials are given relative to Ag/AgCl/3M KCl.

Table SI3: Electrochemical data for complexes **1-5** and **15**. The measurements are performed in 0.1M n-Bu_4NPF_6/CH_3CN at 25 °C (WE: Pt wire; RE: Ag/AgCl/3M KCl; CE: Pt wire). a) The appearance of two oxidation signals is most likely due to the quick decomposition of the complex and not a redox feature of the parent macrocyclized complex **2**).

**Complex**	$E_{1/2}$ (V vs. Ag/AgCl/3M KCl)
**15**	1.00
**4**	1.00
**2**	0.68, 0.93[a)]
**5**	0.70
**3**	0.76
**1**	1.01
